# A Cardiac Mitochondrial FGFR1 Mediates the Antithetical Effects of FGF2 Isoforms on Permeability Transition

**DOI:** 10.3390/cells10102735

**Published:** 2021-10-13

**Authors:** Wattamon Srisakuldee, Barbara E. Nickel, Robert R. Fandrich, Feixong Zhang, Kishore B. S. Pasumarthi, Elissavet Kardami

**Affiliations:** 1 Department of Physiology & Pathophysiology, University of Manitoba, Winnipeg, MB R3E 0J9, Canada; wattamon.srisakuldee@umanitoba.ca; 2 St. Boniface Research Centre, Institute of Cardiovascular Sciences, Winnipeg, MB R2H 2A6, Canada; bnickel@sbrc.ca (B.E.N.); RFandrich@sbrc.ca (R.R.F.); 3 Department of Human Anatomy and Cell Sciences, University of Manitoba, Winnipeg, MB R3E 0J9, Canada; 4 Department of Pharmacology, Faculty of Medicine, Dalhousie University, Halifax, NS B3H 4R2, Canada; fxzhang@cnu.edu.cn (F.Z.); kpasumar@dal.ca (K.B.S.P.)

**Keywords:** FGF2 isoforms, permeability transition, mitochondria, FGFR1, intramitochondrial signalling

## Abstract

Mitochondria, abundant organelles in high energy demand cells such as cardiomyocytes, can determine cell death or survival by regulating the opening of mitochondrial permeability transition pore, mPTP. We addressed the hypothesis that the growth factor FGF2, known to reside in intracellular locations, can directly influence mitochondrial susceptibility to mPTP opening. Rat cardiac subsarcolemmal (SSM) or interfibrillar (IFM) mitochondrial suspensions exposed directly to rat 18 kDa low molecular weight (Lo-) FGF2 isoform displayed increased resistance to calcium overload-induced mPTP, measured spectrophotometrically as “swelling”, or as cytochrome c release from mitochondria. Inhibition of mitochondrial protein kinase C epsilon abrogated direct Lo-FGF2 mito-protection. Exposure to the rat 23 kDa high molecular weight (Hi) FGF2 isoform promoted cytochrome c release from SSM and IFM under nonstressed conditions. The effect of Hi-FGF2 was prevented by mPTP inhibitors, pre-exposure to Lo-FGF2, and okadaic acid, a serine/threonine phosphatase inhibitor. Western blotting and immunoelectron microscopy pointed to the presence of immunoreactive FGFR1 in cardiac mitochondria in situ. The direct mito-protective effect of Lo-FGF2, as well as the deleterious effect of Hi-FGF2, were prevented by FGFR1 inhibitors and FGFR1 neutralizing antibodies. We propose that intracellular FGF2 isoforms can modulate mPTP opening by interacting with mito-FGFR1 and relaying isoform-specific intramitochondrial signal transduction.

## 1. Introduction

Many cardiac pathologies leading to heart failure are intimately linked to mitochondrial dysfunction [1,2]. To maintain their contractile function, cardiomyocytes have high energy demands that are met by their high mitochondrial content. Stresses associated with increased reactive oxygen species production and calcium overload, as in ischemic heart disease, ischemia–reperfusion injury, and cancer drug cardiotoxicity, can promote mitochondrial dysregulation and lead to the formation and opening of mitochondrial permeability transition pore (mPTP), which, in turn, results in cardiomyocyte cell death. Since cardiac repair after cell loss relies mainly on remodelling (hypertrophy, fibrosis) without adequate regeneration, extensive cardiomyocyte death contributes, eventually, to heart malfunction and failure [3]. Understanding mechanisms of mPTP modulation is, therefore, important, in order to induce mito- and cardio-protection by preventing or reducing the extent of cardiomyocyte cell death during a given stress condition.

Fibroblast growth factor 2, FGF2, is a member of the larger family of heparin-binding growth factors and is produced as high molecular weight (>20 kDa), Hi-FGF2, or 18 kDa low molecular weight, Lo-FGF2 isoforms, products, respectively, of CUG- or AUG-initiated translation [4,5]. FGF2 isoforms lack a conventional secretion sequence but can nevertheless be exported to the extracellular space to exert paracrine/autocrine activities by activating cell surface tyrosine kinase receptors (FGFR1, 2, 3, 4) [6,7,8]; FGFR1 is the predominant receptor in the heart and cardiomyocytes [9,10]. It is important to note that both types of FGF2 isoforms, as well as FGFR1, have been localized to cytoplasmic and nuclear sites [11,12]. Thus, in addition to signal transduction initiated at the plasma membrane level, it is plausible that FGF2 and FGFR1 are engaged in intracellular signalling. Both Lo- and Hi-FGF2 are capable of activating signaling via FGFR1 [13]

Previous studies by us and others have demonstrated that both endogenous and administered Lo-FGF2 is a potent agent of acute and sustained cardio-protection in vitro and in vivo [14,15,16]. The protective effects of exogenously administered Lo-FGF2 require binding to sarcolemmal FGFR1 and downstream activation of a signal transduction pathway leading to protection of cardiac mitochondria from calcium-overload-induced mPTP opening and cytochrome c release [17,18].

The effects of Hi-FGF2 are less well defined. Administered, extracellular-acting Hi-FGF2, while acutely cardioprotective, is not protective in a chronic setting [15,19]. Genetic studies have suggested that Hi- and Lo-FGF2 can have different effects on cardiac remodelling after injury [20]. Endogenous Hi-FGF2 exacerbates cardiac damage and dysfunction induced by doxorubicin administration or pressure overload [21,22]. In addition, ectopic expression of Hi-FGF2, but not Lo-FGF2, in neonatal rat cardiac myocytes or HEK293 cells promoted an apoptotic nuclear phenotype and mitochondria-mediated cell death [23,24,25]. The differential effects of FGF2 isoforms on cell death pointed to an intracellular mode of action since extracellular FGF2 activity was neutralized in the above-mentioned studies. To our knowledge, there is no information on the potential direct effects of FGF2 isoforms on cardiac mitochondria. As a first step in addressing this issue, we investigated the effect of direct exposure of isolated cardiac mitochondrial suspensions to the different FGF2 isoforms.

This work will show that Hi-and Lo- FGF2 exert direct and antithetical effects on cardiac mitochondria: Lo-FGF2 is mito-protective, raising resistance to calcium overload-induced mPTP opening. In direct contrast, Hi-FGF2 promotes mPTP opening under normal, nonstressed conditions. A mitochondrial FGFR1-like entity relays the effects of both types of isoforms, suggesting that conditions that control the relative levels of Hi- or Lo-FGF2 in the cytoplasm could determine cell death or survival.

## 2. Materials and Methods

### 2.1. Animals

Adult male Sprague Dawley rats (220–250 g) were provided by the Central Animal Care Facility at the University of Manitoba. Animals were used according to guidelines of the Canadian Council of Animal Care, which is in agreement with the Guide for the Care and Use of Laboratory Animals of the US National Institutes of Health. The experimental protocols used were approved by the local Animal Care Committee of the University of Manitoba.

### 2.2. Materials

Both rat low molecular weight, 18 kDa, Lo-FGF2 (or FGF2), and high molecular weight, 23 kDa, Hi-FGF2, were produced in *Escherichia coli* and purified as published by us previously [7,8]. FGF2 isoforms were used within one month of preparation. The activity was assessed by examining the potency of the isoforms towards activating extracellular-signal-regulated kinase (ERK) by increasing relative levels of phospho-(p)-ERK in cardiac fibroblasts; both types of isoforms routinely exhibited similar potency in increasing p-ERK. Protein kinase C (PKC)ε—specific inhibitor peptide (PKCεV_1-2_, EAVSLKPT) and the control (PKCε scrambled peptide, LSETKPAV) (# 539522 and 539542, respectively), were purchased from Calbiochem, San Diego, CA, USA. The peptides were used at 0.5 µM, as previously published [26]. FGFR1 inhibitors SU-5402, PD-173074, protease inhibitor cocktail (PIC), cyclosporin A, and alamethicin were from Sigma, Oakville, ON, Canada. Phosphatase inhibitor cocktails (PPICs) set 2 and set 4 were from Calbiochem, San Diego, CA, USA. ECL Plus Western Blotting substrate was from Pierce, Rockyford, IL, USA.

### 2.3. Antibodies

Goat antibodies against adenine nucleotide translocase (ANT, #sc-929) were from Santa Cruz Biotechnology, Dallas, TX, USA. Mouse monoclonal antibodies against cyclophilin (#MSA04) D and cytochrome c (#556433), were, respectively, from MitoSciences, Eugene, OR, USA, and BD Biosciences Pharmingen, Mississauga, ON, Canada. Rabbit anti-FGFR1 (#sc-121), anti-pY766 (#16309-R), anti-pY653/654 (#30262-R), all recognizing sites at the catalytic C-terminal domain, were from Santa Cruz Biotechnology. Neutralizing anti-FGFR1 antibody (#MAB125, ligand-binding domain) was from Millipore Sigma, Oakville, ON, Canada. Rabbit-affinity-purified anti-FGFR1 (#F5421) was from Sigma, and mouse monoclonal anti-FGFR1 (#30101; M19B2; QED A/B, ligand-binding domain) was from QED Bioscience Inc. (San Diego, CA, USA). Mouse anti-Shc (#610878) was from BD Transduction, while anti-pY239/240-Shc (sc-18074-R) from Santa Cruz Biotechnology. Donkey anti-rabbit horseradish peroxidase (HRP), #711-035-152, and anti-mouse HRP, #715-035-150, as well as anti-goat HRP, #705-035-147, were from Jackson Immuno Res. Lab. Secondary anti-rabbit antibodies used in immunoelectron microscopy were coupled to 10 nm gold particles (Sigma, Oakville, ON, Canada).

### 2.4. Mitochondrial Isolation

Rat cardiac subsarcolemmal (SSM), or interfibrillar (IFM) mitochondria were prepared exactly as described by us previously. These preparations are devoid of any detectable contamination from other cellular components [17]. Liver mitochondria were obtained as described in [27].

### 2.5. Mitochondrial Matrix Swelling by Calcium Overload

The mPTP opening was examined by Ca^2+^ -induced matrix swelling, measured as a reduction of optical density at 545 nM (OD)-545 as described in [17]. Briefly, isolated mitochondria were suspended in “swelling buffer” at a final concentration of 0.5 mg/mL and the absorbance measured spectrophotometrically at 545 nm. Small increments of 125 nM CaCl^2+^ were added until there was no further change in absorbance. At the end of the experiment, 100% swelling was determined by the addition of alamethicin (15 µg/mL), an antibiotic that forms a large pore. The experiments were conducted in the presence or absence of cyclosporine A, CsA, a potent mPTP inhibitor to ensure that our measurements were mediated by mPTP opening.

### 2.6. Release of Cytochrome c from Mitochondrial Suspensions

This procedure was carried out as described in [27]. Briefly, mitochondria were suspended at 1 mg /mL in assay buffer (120 mM KCl, 10 mM HEPES pH 7.4, 10 mM succinate, 5 mM KH_2_PO_4_, 0.5 mM MgCl_2_). Mitochondria suspensions (100 μL) were incubated in uncapped tubes at 30 °C. Inhibitors, neutralizing antibodies, or vehicle solution were added to mitochondria for 15 min, followed by exposure to FGF2 isoforms for a further 15 min. Samples were then centrifuged at 21,000× *g* for 5 min at 4 °C. To determine relative cytochrome c release, an equal volume of supernatant (80 μL) was carefully removed from each sample and immediately added to 320 μL of sterile distilled H_2_O. A fraction (20 μL/sample) of the diluted supernatant was added to 20 μL of 2-fold concentrated SDS sample buffer. These dilutions were necessary to reduce the KCl concentration to allow analysis by SDS–PAGE. Any liquid remaining with the pellets was carefully removed using a gel loading tip, and pellets were washed with 150 μL of assay buffer. After all liquid was removed, 40 μL of 1 × SDS sample buffer supplemented with a mixture of protease and phosphatase inhibitors was added to the pellets and samples sonicated before analysis by Western blotting. Cytochrome c release was analyzed using 15% polyacrylamide gels and Western blotting.

### 2.7. Mitochondrial Respiration Assay

Mitochondrial respiration was measured polarographically with a Clark-type electrode at 30 °C in a 1 mL sealed chamber (Quibit Systems Inc, Kingston, ON, Canada) with magnetic stirring as described.

### 2.8. SDS–PAGE and Western Blotting

Cardiac mitochondrial particulates (pellet, 25–35 µg) and supernatant fractions were resolved by SDS–PAGE, mostly on 10% polyacrylamide gels, and transferred onto polyvinylidene difluoride membranes (Roche).

### 2.9. Immunoelectron Microscopy (EM)

Small pieces (2 × 2 mm) of cardiac ventricles, fixed overnight in 4% paraformaldehyde and 0.5% glutaraldehyde in 0.1 M sodium cacodylate buffer and processed for immuno-EM as described in [28]. Primary antibodies used were specific for total FGFR1 (F5421, Sigma; sc-121) or pY653/654-FGFR1 #30262-R, from Santa Cruz. Secondary anti-rabbit immunoglobulin (IgG) antibodies used were coupled to 10 nm gold particles (Sigma, Oakville, ON, Canada). Samples were viewed under a JEOL JEM 1230 Transmission Electron Microscope at 80 kV. Images were captured using a Hamamatsu ORCA-HR digital camera.

### 2.10. Statistical Analysis

Differences between groups were compared using Student’s *t*-test (unpaired), and one or two-way analysis of variance (ANOVA) as required; *p* < 0.05 was considered significant (GraphPad PRISM 7.0, San Diego, California, USA). Data are presented as means ± SEM.

## 3. Results

### 3.1. The Effect of Lo-FGF2 on Mitochondrial Resistance to Calcium-Overload-Induced Formation of mPTP—The Role of Mitochondrial Protein Kinase C (PKC) ε

Freshly isolated cardiac SSM suspensions were exposed to a stressor, calcium overload, in the absence or presence of added Lo-FGF2. Mitochondrial swelling, indicative of mPTP opening, was measured by the decrease in light scattering, optical density (OD 545), of the suspensions. We have previously shown that calcium-induced mitochondrial swelling, as measured in our experiments, is fully prevented in the presence of the mPTP inhibitor cyclosporine A, CsA [17].

**Figure 1:**
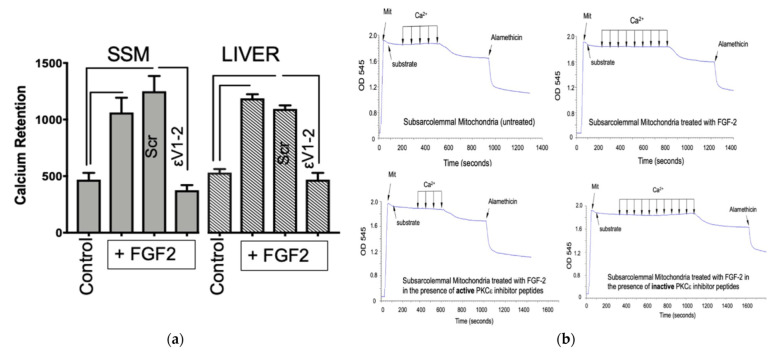
Direct exposure to Lo-FGF2 increases mitochondrial calcium retention capacity by a mito-PKCε mechanism: (**a**) Lo-FGF2 significantly (*n* = 4, *p* < 0.5) increases calcium retention capacity, expressed in μm Ca^2+^/mg protein, in both cardiac (SSM) and liver mitochondrial suspensions, as indicated. The PKCε inhibitory peptide εV_1-2_, but not the inactive scrambled (Scr) peptide, abolished the protective effect of FGF2. Brackets point to statistically significant differences between groups (*p* < 0.05, *n* = 4); (**b**) representative tracings showing the changes in optical density, OD 545, values, (indicative of swelling and calcium capacity) in the different SSM groups as a function of time (seconds) and calcium increment. Arrows indicate each calcium increment (by 125 μM).

As shown in Figure 1a, Lo-FGF2 increased calcium retention capacity, indicating increased resistance to mPTP. SSM contain PKCε, which has been shown to regulate vulnerability to mPTP [29]. Inclusion of the PKCε-inhibitory peptide εV_1-2_, but not the inactive scrambled peptide, completely blocked the mito-protective ability of Lo-FGF2. The same experiment was repeated using rat liver mitochondrial suspensions. Lo-FGF2 was similarly protective for liver mitochondria, by a mito-PKCε-activity-dependent pathway, as indicated (Figure 1a). The ability of Lo-FGF2 to directly exert mito-protection is not, therefore, exclusive to heart mitochondria. Representative tracings show SSM changes in OD 545 with calcium increments over time, in the presence of Lo-FGF2 and PKCε inhibition (Figure 1b).

Numerical values for calcium retention capacity, rate of swelling, and magnitude of swelling, for the SSM experiment, shown in Figure 1, can be found in Table S1. No significant changes were observed between groups regarding the rate of swelling or magnitude of swelling (Table S2). Lo-FGF2 did not influence SSM respiration, assessed polarographically using glutamine and malate as oxidative substrates, as shown in Table S2. The respiratory control index (RCI) represented the ratio of state 3/state 4 respirations and was not affected by the presence of Lo-FGF2. The RCI of cardiac mitochondria in the current experiments was higher than 7.0. FGF2 did not significantly affect the rate of oxidative phosphorylation. Mitochondrial preparations were considered well coupled based on their RCI values being higher than 4.0 [30].

### 3.2. Probing for a Mitochondrial FGF2 Receptor, Mito-FGFR1

**Figure 2:**
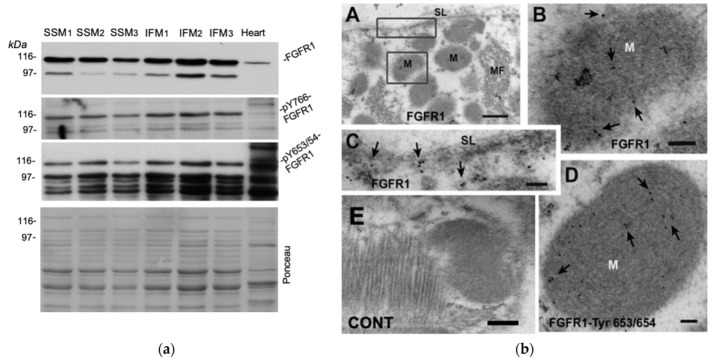
Immunoreactive FGFR1 is present in cardiac mitochondria: (**a**) Western blots of subsarcolemmal (SSM; *n* = 3) and interfibrillar (IFM; *n* = 3) preparations probed for total FGFR1 (QED A/B), pY766-FGFR1 (sc-16309-R), and pY653/654-FGFR1 (sc-30262), as indicated, respectively by -FGFR1, -pY766-FGFR1, -pY653/654-FGFR1. Immunoreactive FGFR1 is detected in SSM and IFM preparations; (**b**) immunogold labelling for FGFR1 in cardiomyocytes in situ. Picture A represents a lower magnification of an ultrathin section processed for anti-FGFR1 staining; affinity-purified rabbit anti-FGFR1, #F5421. Boxed areas corresponding to mitochondria (M) and sarcolemma (SL) are shown as higher magnifications in pictures B and C, respectively. Picture D shows cardiac mitochondrial staining by anti-pY653/54-FGFR1, #sc-30262-R. Picture E represents a control (CONT) section where primary anti-FGFR1antibody was omitted but was incubated with gold-labelled secondary antibodies. MF = myofilaments. Scale bars: A, 500 nm: B–E = 100 nm.

The ability of Lo-FGF2 to directly affect mitochondria by exerting mito-protection and, presumably, to activate mito-PKCε, suggested the possibility that an FGF2-responsive receptor, such as an FGFR1-like entity, may be located and operating at the mitochondrial level, relaying the protective effect of Lo-FGF2. Detection of FGFR1 in mitochondria has been reported previously for cancer cell lines [31]. To address this question, three different preparations of SSM and IFM were probed with antibodies for total FGFR1, and tyrosine (Y) phosphorylated, presumably activated, versions, pY653/654- and pY766-FGFR1 by Western blotting. The FGFR1 antibodies used here have been previously validated: they were capable of detecting mouse FGFR1 overexpressed in the human embryonic kidney (HEK)293 cells, at the expected molecular size [32]; see also Figure S1. Western blotting results are shown in Figure 2a. All SSM and IFM preparations displayed immunoreactive bands in the 80–110 kDa range detected by all antibodies consistent with the presence of FGFR1 in cardiac mitochondria; reactivity against phospho-FGFR1 indicated that the mitochondrial receptor was present, at least in part, in an activated state (Figure 2a). In parallel experiments, mitochondria isolated from hearts stimulated briefly with Lo-FGF2 displayed stronger signals for pY766- and pY653/654 -FGFR1 (at 80–110 kDa), compared with those from unstimulated hearts, as shown and described in more detail in Figure S2. We have previously shown that rodent hearts express short (~80 kDa) and long (~102 kDa) FGFR1 isoforms, products of differential splicing [9]. FGFR1 is subject to multiple posttranslational modifications, including phosphorylation and glycosylation, explaining the appearance of multiple closely spaced bands in Western blots; an example of multiple anti-FGFR1 bands in a different context can be found in reference [33]. Immuno-EM of cardiac sections was also used to detect mito-FGFR1 in situ. Using the anti-FGFR1, #F5421, antibodies, positive immunogold staining was observed at mitochondria (M), as well as cardiomyocyte sarcolemma, (SL, Figure 2b, pictures A, B, C). A positive signal at mitochondria was also obtained with anti-pY653/654-FGFR1 (Figure 2b, picture D). Control sections incubated with nonimmune rabbit immunoglobulin, followed by immuno-gold labelled secondary reagents, did not show any signal (Figure 2b, picture E).

**Figure 3:**
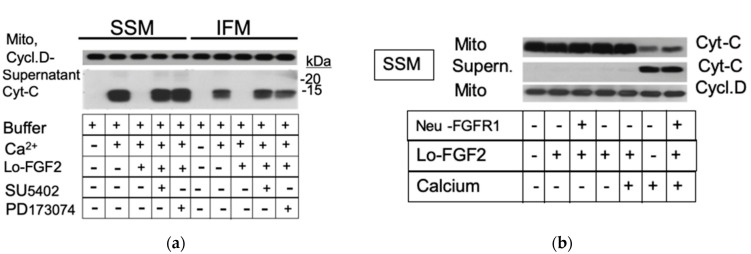
FGFR1 inhibition or neutralization prevents mito-protection by Lo-FGF2 against calcium overload: (**a**) Western blots of mitochondrial particulate fraction (Mito) and Supernatant fraction, probed for cyclophilin D (Cycl.D) or cytochrome c (Cyt-C), as indicated. Electrophoretic migration of markers is shown as -20 and -15 kDa. The Lo-FGF2 protection from calcium-induced cytochrome c release (mPTP) to the supernatant is prevented by both FGFR1 inhibitors SU-5402 and PD-173074 in both SSM and IFM; (**b**) Western blots of mitochondrial (SSM) particulate fraction (Mito) and supernatants, probed for cyclophilin D (Cycl.D) or cytochrome c (Cyt-C), as indicated. The Lo-FGF2 protection from calcium-induced cytochrome c release (mPTP) to the supernatant is prevented by anti-FGFR1 neutralizing antibodies, neu-FGFR1. Cytochrome c in the particulate (Mito) mitochondrial suspension is reduced when supernatant cytochrome c is increased; Cycl.D indicates similar protein amounts between Mito samples.

Mitochondria subjected to calcium overload in situ and in the test tube release cytochrome c to their environment; cytochrome c release to the cytosol will initiate a cascade of events and pathways leading to apoptotic and necrotic cell death [34,35]. Extracellular administration of Lo-FGF2 has been demonstrated to prevent cytochrome c release from cardiac mitochondria [17]. We now tested the effect of direct mitochondrial exposure to Lo-FGF2 towards calcium-overload-induced cytochrome c release in the absence or presence of FGFR1 inhibitors. Early generation ATP-competitive FGFR tyrosine kinase inhibitors SU-5402 and PD-173074 were used for these studies; both have been used extensively in the past [36,37]. SU-5402 inhibits FGFR1 and other tyrosine kinases, while PD-173074 shows higher selectivity towards FGFR1, as per distributors’ datasheets. Representative findings are shown in Figure 3a. Lo-FGF2 prevented calcium-induced cytochrome c release from both SSM and IFM. The protective effect of Lo-FGF2 was completely blocked by SU-5402 and PD-173074. Staining of the corresponding particulate mitochondrial fraction (Mito) for cyclophilin D is also included, to indicate equivalent loading of gel lanes.

Neutralizing antibodies for FGFR1 were also used to test their effect on mito-protection by Lo-FGF2. Representative findings are shown in Figure 3b. As expected, Lo-FGF2 prevented calcium-induced cytochrome c release from SSM. Neutralizing anti-FGFR1 antibodies (10 μg/mL) abolished the protective effect of Lo-FGF2 against cytochrome c release to the supernatant (Figure 3b). Analysis of the corresponding particulate mitochondrial fraction (Mito) by Western blotting showed a decrease in cytochrome c content, inversely mirroring the increase of this protein in the supernatant; staining of Mito for cyclophilin D confirmed similar protein content between lanes.

### 3.3. The Effects of Hi-FGF2 on Cardiac Mitochondria

**Figure 4:**
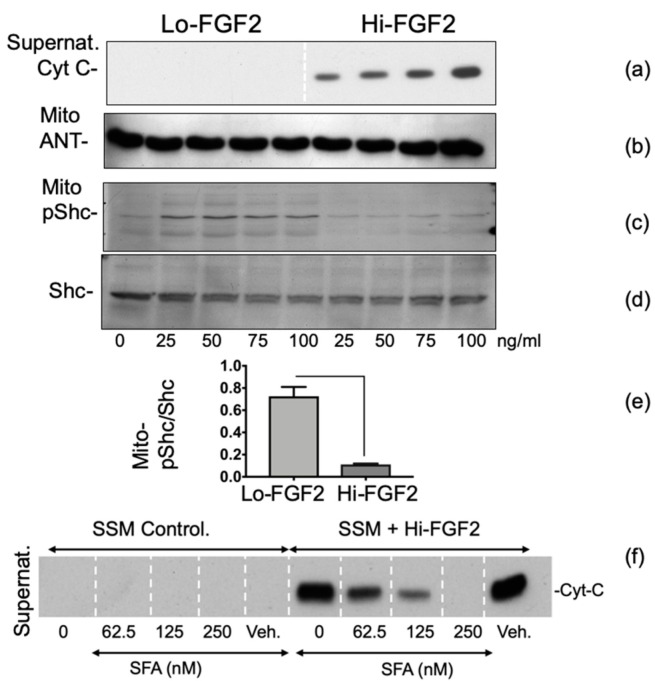
Direct detrimental effects of Hi-FGF2 on cardiac mitochondria: (**a**) Hi-FGF2 promotes cytochrome c release from isolated SSM mitochondria. Western blot showing cytochrome c (Cyt-C) detection in supernatants of SSM suspensions after exposure to either Lo-FGF2 or Hi-FGF2, at 0, 25, 50, 75, 100 ng/mL, as indicated; (**b**) Western blot showing that ANT remains essentially unchanged in SSM mitochondria regardless of Lo-FGF2 or Hi-FGF2 exposure. The ANT indicates similar protein loading and integrity of the inner mitochondrial membrane; (**c,d**) exposure to Lo-FGF2, but not Hi-FGF2, increases mitochondrial pShc without affecting total Shc. SSM exposed to Lo- or Hi-FGF, at 25–100 ng/mL, as indicated, and probed for pShc (**c**) or Shc, (**d**); (**e**) ratio of pShc/Shc at 25–100 ng/mL Hi- or Lo-FGF2, is significantly higher in Lo-FGF2-treated SSM compared with those treated with Hi-FGF2 (*n* = 4. *p* < 0.05); (**f**) Hi-FGF2 induces mPTP in SSM. Western blot of SSM supernatants probed for cytochrome c after exposure to Hi-FGF2, in the presence of 0, 62.5, 125, 250 nM sanglifehrin A (SFA), an inhibitor of mPTP. SFA decreased or eliminated cytochrome c release in a dose-dependent manner. SFA was dissolved in ethanol (vehicle, Veh) which, by itself, did not promote cytochrome c release. In the absence of Hi-FGF2, SFA did not promote cytochrome c release.

We next examined the direct effect of Hi-FGF2 (25–100 ng/mL) on SSM suspensions, under nonstressed conditions—namely, in the absence of calcium overload. Mitochondria treated with Hi-FGF2 released cytochrome c to the supernatant at all Hi-FGF2 concentrations used (Figure 4a). In comparison, Lo-FGF2 used in the same concentration range did not stimulate cytochrome c release. Staining of the corresponding particulate fraction for adenine nucleotide translocase (ANT) indicated equivalent protein loading and integrity of the inner mitochondrial membrane.

Shc (Src homology and collagen-containing) protein is an adaptor protein produced as 44, 52, and 66 kDa versions; Shc associates with and becomes phosphorylated on tyrosine residues (pY239/240, pY317) by receptor tyrosine kinases including FGFR1 in cells [38]. As Shc is present in mitochondria [39], we hypothesized that it could represent an immediate target of intramitochondrial mito-FGFR1 signalling. We, therefore, stimulated SSM with Lo- or Hi-FGF2 and used Western blotting to examine relative levels of phospho-Shc and total Shc in the particulate fraction. As shown in Figure 4c,d, exposure to Lo-FGF2 resulted in higher levels of a 52 kD mitochondrial pY239/240-Shc, compared with those after Hi-FGF2 stimulation. Total Shc (52 kDa) was relatively unchanged between the two groups treated with Lo- or Hi-FGF2. The graph in Figure 4d shows the ratio of pYShc/total Shc, after grouping together values for 25–100 ng/mL of each isoform.

Sanglifehrin A (SFA) represents a pharmacological inhibitor of mPTP opening. As seen in Figure 4f, SFA prevented the Hi-FGF2-induced release of cytochrome c to the SSM supernatant, in a dose-dependent manner, validating the notion that Hi-FGF2 promotes mPTP opening.

**Figure 5:**
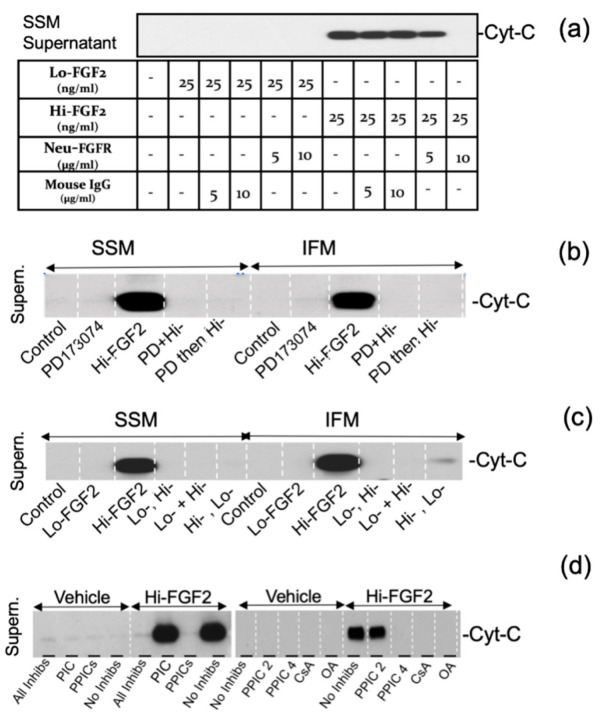
The Hi-FGF2-induced cytochrome c release from cardiac mitochondria is blocked by: FGFR1 neutralization; the FGFR1 inhibitor PD-173074; Lo-FGF2; cyclosporine A; Ser/Thr phosphatase inhibition; (**a**) Western blot showing cytochrome c (Cyt-C) in the supernatant fraction from SSM suspensions, after they were treated with Lo-FGF2 or Hi-FGF2, as indicated, at 25 ng/mL. Neutralizing anti-FGFR1 antibodies, Neu-FGFR, used at 5 or 10 μg/mL decreased or eliminated, respectively, the Hi-FGF2-induced cytochrome c release. Nonspecific mouse IgG had no effect; (**b**) Western blot showing cytochrome c (Cyt-C) in the supernatant fraction from Hi-FGF2-treated SSM or IFM suspensions. The FGFR1 inhibitor PD-173074 added to SSM or IFM suspensions together with Hi-FGF2, (PD + Hi-), or prior to Hi-FGF2 (PD, Hi-), prevented the Hi-FGF2-induced cytochrome c release, as indicated; (**c**). Western blot showing cytochrome c (Cyt-C) in the supernatant fraction from Hi-FGF2-treated SSM or IFM suspensions. Lo-FGF2 was added to SSM or IFM prior to Hi-FGF2, (Lo, Hi), together with Hi-FGF2, (Lo + Hi), or immediately after Hi-FGF2 (Hi, Lo), and prevented the Hi-FGF2-induced cytochrome c release, as indicated. Both Lo- and Hi-FGF2 were used at 25 ng/mL; (**d**) Western blots of supernatants from SSM suspensions incubated with either vehicle or vehicle + Hi-FGF2, in the presence of various protease and phosphatase inhibitor cocktails and individual inhibitors. Lanes are labelled as follows, from left to right: All Inhibs (mix of protease and phosphatase inhibitors, PIC (protease inhibitors), PPICs (protein phosphatase inhibitors mix), No Inhibs (no inhibitors have been included), PPIC 2 (phosphatase inhibitors set 2), PPIC 4 (phosphatase inhibitors set 4), the mPTP inhibitor Cyclosporin A (CsA) at 10 nM, and the PP1 and PP2A inhibitor okadaic acid (OA) at 100 nM.

The ability of neutralizing anti-FGFR1 antibodies (5–10 μg/mL) to influence the effects of Hi-FGF2 on SSM was also tested, and representative results are shown in Figure 5a. Neutralizing antibodies at 10 μg/mL blocked cytochrome c release induced by Hi-FGF2. Nonspecific antibodies used at similar concentrations had no effect; Lo-FGF2 did not induce cytochrome c release, as shown in Figure 5a.

We asked if Hi-FGF2 would promote cytochrome c release from IFM, in comparison to SSM, mitochondria, in the absence and presence of FGFR1 inhibition by PD-173074. Representative findings are shown in Figure 5b. Both SSM and IFM responded to Hi-FGF2 in a similar fashion by releasing cytochrome c to the supernatant. In addition, PD-173074 added either prior to or at the same time as Hi-FGF2, prevented the Hi-FGF2-induced cytochrome c release from both SSM and IFM mitochondria (Figure 5b).

Pre- or simultaneous exposure to an equivalent amount of Lo-FGF2 (25 ng/mL), prevented the Hi-FGF2-induced cytochrome c release from both SSM and IFM (Figure 5c).

While these experiments were conducted, we accidentally used a solution containing a mixture of phosphatase and protease inhibitor cocktails (PIC and PPICs) and found that it prevented Hi-FGF2 from promoting cytochrome c release. This serendipitous observation was explored further, to determine which component(s) of the cocktails might have the inhibitory effect. Representative results are shown in Figure 5d. The Hi-FGF2 effect was not prevented by the protease inhibitor cocktail (PIC), nor by a phosphatase inhibitor cocktail (PPIC set 2) expected to inhibit acid and alkaline phosphatases and protein tyrosine phosphatases. The effect of Hi-FGF2 was prevented by a phosphatase inhibitor cocktail expected to inhibit alkaline phosphatase, and serine/threonine phosphatases, such as protein phosphatase 1 (PP1) and protein phosphatase 2A (PP2A) phosphatases (PPIC set 4); and by okadaic acid, also an inhibitor of PP1 and PP2A phosphatases (Figure 5d). Taken together, our results indicate the effects of Hi-FGF2 are mediated by mito-FGFR1 and require serine/threonine phosphatase activity.

## 4. Discussion

The main findings presented here are as follows: (i) Lo-FGF2 or Hi-FGF2 exert, respectively, direct, beneficial, or detrimental effects on isolated cardiac mitochondria; (ii) mitochondria possess a functional FGFR1-like receptor; (iii) mito-FGFR1 relays the beneficial effects of Lo-FGF2 via mito-PKCε activity, and the deleterious effects of Hi-FGF2 via PP1 or PP2A-type phosphatase(s) activity.

Direct effects of FGF2 isoforms on mitochondria: Exposure of cardiac mitochondrial preparations to Lo-FGF2 was mito-protective against calcium-overload stress, as it raised mitochondrial resistance to mPTP, assessed either as mitochondrial swelling, or release of cytochrome c from mitochondria. Importantly, the mito-protective effect of Lo-FGF2 on cardiac SSM, as well as liver mitochondria that were included for comparative purposes, was abolished in the presence of mito-PKCε inhibition. Thus Lo-FGF2 is capable of directly activating mito-PKCε which then contributes to mPTP prevention; this response, furthermore, does not appear to be specific to heart mitochondria since it was also observed in mitochondria from the liver. The role of mito-PKCε in mito-protection is in agreement with previous studies [29,40]. However, Lo-FGF2 was directly protective towards IFM that have negligible levels of mito-PKCε when isolated from non-stimulated hearts; other kinase(s) localizing in mitochondria, such as glycogen synthase kinase 3 beta, GSK3β, which is present in IFM [17], might mediate the direct Lo-FGF2 mito-protection.

In contrast to Lo-FGF2, exposure to Hi-FGF2 was considered detrimental as it promoted cytochrome c release through mPTP opening. The involvement of mPTP was supported by the ability of two different mPTP inhibitors, SFA, and CsA to block the Hi-FGF2 induced cytochrome c release to the supernatant fraction of mitochondrial suspensions. Indirect support for mPTP involvement was provided by the ability of Lo-FGF2 (which prevents calcium overload-induced mPTP) to also prevent the effect of Hi-FGF2. The release of cytochrome c from mitochondria is an indicator of mitochondrial dysfunction and deterioration; furthermore, in the cellular context, cytochrome c release to the cytosol stimulates a series of events that promote cell death [34].

The presence of FGFR1 in cardiac mitochondria was suggested by the ability of Lo-FGF2 or Hi-FGF2 to exert their effects directly. Support for this notion was obtained by several independent pieces of evidence. Firstly, Western blotting of SSM and IFM suspensions with several antibodies recognizing either total FGFR1 (raised against the ligand-binding domain), or activated FGFR1, recognizing the catalytic tyrosine kinase domain when phosphorylated at Y766 or Y653/54. This is particularly compelling in the case of IFM preparations that have undergone incubation with proteolytic enzymes to strip away myofibrillar and other proteins which may co-purify or are associated with mitochondria but are not integral to the mitochondrial structure. Secondly, immuno-EM indicated the presence of FGFR1 in cardiac mitochondria, as well as sarcolemma (as expected), in situ. Thirdly, antibodies capable of binding to the extracellular domain of FGFR1 and preventing interaction with ligands also prevented the effects of either Lo- or Hi-FGF2. Finally, two different pharmacological inhibitors of FGFR1, SU-5402 and PD-173074, prevented the effects of either type of isoform. Overall, and despite contrasting end-points, both types of isoforms appear to require binding to mito-FGFR1 to exert their effects.

Other than the detection of FGFR1 in mitochondria from cancer cell lines [31], to our knowledge, there are no previous reports on the presence of FGFR1 specifically in heart mitochondria. Tyrosine kinase receptors such as the receptor for a nerve growth factor (NGF, neurotrophin) and, epidermal growth factor receptor, EGFR, have been detected in mitochondria [41]. In mitochondria isolated from the rat brain cortex, NGF tyrosine kinase receptors mediate direct protective effects of NGF against mPTP [42]. In addition, the mito-EGF receptor was associated with the cytochrome c oxidase subunit II in mitochondria and was shown to promote cell survival [43].

In addition to plasma membrane localization, FGFR1 can exist as a cytoplasmic, “membrane-bound” entity and can also translocate to the nucleus [44,45,46]. Work shown here suggests that at least a fraction of cytoplasmic, membrane-bound FGFR1 represents mitochondrial FGFR1. Lacking a specific targeting sequence, FGFR1 could theoretically translocate to mitochondria by a mechanism employing the TOM translocase complex and interaction with heat shock protein 90, HSP90, in a manner similar to other proteins such as connexin-43 or PKCε. FGFR1can interact with, and could potentially be, a “client” of HSP90 [47]. FGF2 binding to plasma membrane FGFR1 promotes receptor internalization and translocation to the nucleus [45]; one can speculate that it could also promote translocation to mitochondria. Plasma membrane EGFR is reported to translocate to mitochondria upon ligand binding [48].

Mitochondrial presence of FGFR1, together with several signaling molecules known to become activated downstream of FGFR1 within the cell, introduces the possibility of FGFR1-dependent intra-mitochondrial signal transduction linked to the regulation of mPTP. It is widely understood that mPTP represents a dynamic, multi-molecular entity [1,2]. Numerous protein–protein interactions involving all mitochondrial domains, such as the inner mitochondrial membrane, intermembrane, and matrix spaces, and proteins that translocate to and associate with mitochondria are capable of modulating mPTP formation and opening. Several kinases including PKCε, ERK, AKT, GSKβ, src, which become activated downstream of plasma membrane FGFR [6], have also been detected in mitochondria, where they contribute to mPTP regulation. Mitochondrial resident phosphatases have also been described [49]. It is plausible therefore that some of these kinases and phosphatases can become activated within mitochondria downstream of mito-FGFR1 and thus affect mPTP opening.

The question arises as to how the same receptor can be used to achieve different endpoints by Lo- or Hi-FGF2. Both types of isoforms are considered equally capable of interacting with and activating plasma membrane FGFR1 [6], although systematic and context-dependent studies have not been conducted. It is important to determine the relative concentration of FGF2 isoforms, as well as their affinities for FGFR1 in various cell types, in normal and stressed conditions, and over time in vivo. It was shown here that when both isoforms were used together at 25 ng/mL each, Hi-FGF2 was unable to elicit mPTP opening. This would suggest that Lo-FGF2 binds mito-FGFR1 with a higher affinity than Hi-FGF2 and is thus able to reduce/prevent Hi-FGF2 binding. It is also possible that the outcome of the interaction of each FGF2 isoform with mito-FGFR1 is differentially modulated by co-receptors or other proteins present at mitochondria. It is intriguing that exposure to Hi-FGF2, unlike Lo-FGF2, did not result in increased mito-Shc phosphorylation on tyrosine, suggestive of disrupted or diverted mito-FGFR1 signal transduction. It is possible that Hi-FGF2 binding to mito-FGFR1 is not able to elicit receptor tyrosine phosphorylation and subsequent recruitment of downstream molecules such as Shc. Alternatively, or concurrently, activation of a PP2A-like mitochondrial phosphatase in response to Hi-FGF2 might result in dephosphorylation of signaling molecules that are important for protection from mPTP. Interaction of PP2A with Shc is reported to inhibit growth factor signaling in the whole-cell context [50] and can be hypothesized to play a similar role in intramitochondrial signaling. The prevention of Hi-FGF2-induced mPTP opening by okadaic acid, a PP2A inhibitor, provides support to the notion of a serine/threonine-phosphatase mediated signaling by Hi-FGF2.

In the short term, extracellular-acting Lo- or Hi-FGF2 are similarly able to protect cardiomyocytes in culture, perfused hearts ex vivo, and infarcted hearts in vivo, presumably acting mainly through plasma membrane FGFR1. On the other hand, chronic studies have shown that endogenously expressed Hi-FGF2 (unlike Lo-FGF2) is not protective but rather it contributes to increased cardiac vulnerability to various stresses and reduces relative levels of cardiac pY-FGFR, which, based on findings presented here, likely consists of both sarcolemmal and mito- FGFR1 [16,21]. In view of the present findings, it is possible that endogenous cytosolic Hi-FGF2 might prevent mito-FGFR1 phosphorylation and activity, and contribute to mitochondrial vulnerability and dysfunction, regardless of the effects of extracellular Hi-FGF2 on plasma membrane FGFR1.

A limitation of our studies is that they were conducted on isolated mitochondria and remain to be shown to operate in a similar fashion in vivo. Detection of mito-FGFR1, as well as its activated version, pY653/654 FGFR1, in cardiac mitochondria by immuno-EM of heart sections, argues in favour of the notion that cytosolic FGF2 would exert direct effects on mitochondria in the whole cell and organ context. Overexpression studies have already indicated that intracellular Hi-FGF causes cell death requiring mitochondrial engagement [25], in support of this study and of the notion that mito-FGFR1 operates at the cellular level.

Extrapolating our findings to the in vivo setting suggests that the relative cytosolic levels of the FGF2 isoforms, whether freshly synthesized or internalized from the extracellular environment, could regulate baseline mitochondrial, and therefore cellular, resistance to injury by interacting with mito-FGFR1. Exciting questions that remain to be addressed in the future pertain to the exact mitochondrial localization and relative orientation of FGFR1, its proximity interactions with mitochondrial proteins, enzymes, and putative mPTP components in health and disease. Finally, the relative contributions of plasma membrane FGFR1-initiated signaling versus mito-FGFR1 signaling to cell viability need to be better understood to fine-tune potential therapeutic interventions aiming at FGFR1 inhibition.

## Data Availability

All available data are in Supplementary Materials.

## Supplementary Materials

The following are available online at https://www.mdpi.com/article/10.3390/cells10102735/s1, Table S1: The effect of calcium overload, Lo-FGF2, and PKCε inhibitors on cardiac (SSM) mitochondrial properties, Table S2: The effect of Lo-FGF2 on cardiac mitochondria (SSM) Respiration, Figure S1: Testing of anti-pY- FGFR1 antibodies, Figure S2: Lo-FGF2 promotes mito-FGFR1 phosphorylation in hearts. Additionally included in Supplementary Materials: The original, uncut gel and other images used to create Figures in the main text; the original spectrophotometry tracings used to create Figure 1, and reproduced (additional)experimental data (Western blots) to support data shown in the main text.
