# Supplementary material for: A Cardiac Mitochondrial FGFR1 Mediates the Antithetical Effects of FGF2 Isoforms on Permeability Transition
Source: Cells. 2021 Oct 13;10(10):2735. doi: 10.3390/cells10102735 (PMC8534529; doi:10.3390/cells10102735)

Figure S1 Testing of anti-pY- FGFR1 antibodies
Human endothelial kidney (HEK) 293 cells were transfected or not with a plasmid expressing mouse FGFR1 and stimulated or not with Lo-FGF2 (10 ng/ml, 15 minutes). Subsequently, cell lysates were analyzed by western blotting and probed with antibodies for pY766-FGFR1, pY653/654-FGFR1, or beta (β)-tubulin, as indicated.
Unstransfected cells presented negligible signal for pY766-FGFR1 , and detectable signal for pY653/654-FGFR1. In both cases, the immunoreactive signal increased substantially in transfected cells, especially after stimulation with Lo-FGF2. Intensity of signal for β–tubulin (loading control) appeared similar between the different samples.

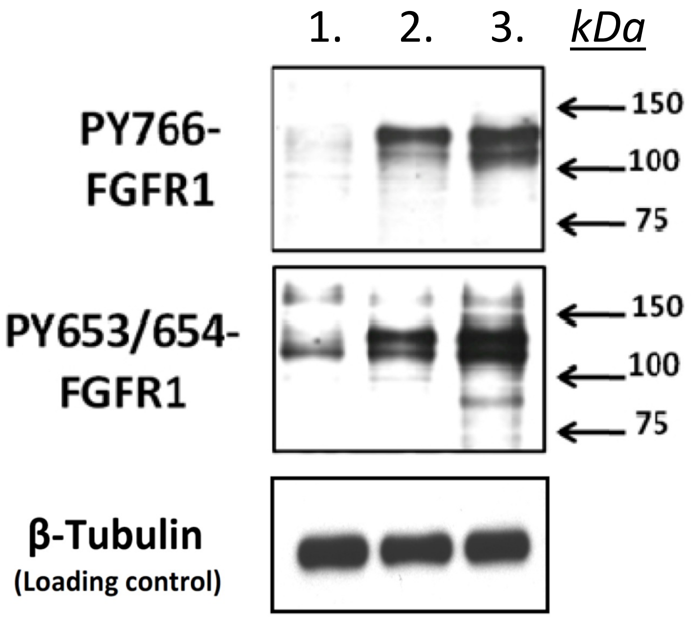

1. Untransfected HEK 293 cells
2. Cells transfected with cDNA for FGFR1
3. Cells transfected with a cDNA for FGFR1 + FGF2

Figure S2. **Lo-FGF2 promotes mito-FGFR1 phosphorylation in hearts**. Western blot staining of cardiac mitochondria (IFM) from saline-perfused (lanes 1,2,3, three preparations) and hearts stimulated with Lo-FGF2 for 15 minutes, lanes 4,5,6, three preparations) for pY-FGFR1. Ponceau S staining indicates similar protein loading between all lanes. A stronger signal for immunoreactive bands (70-110 kDa) can be observed in lanes 4,5 and 6, compared to lanes 1,2 and 3.

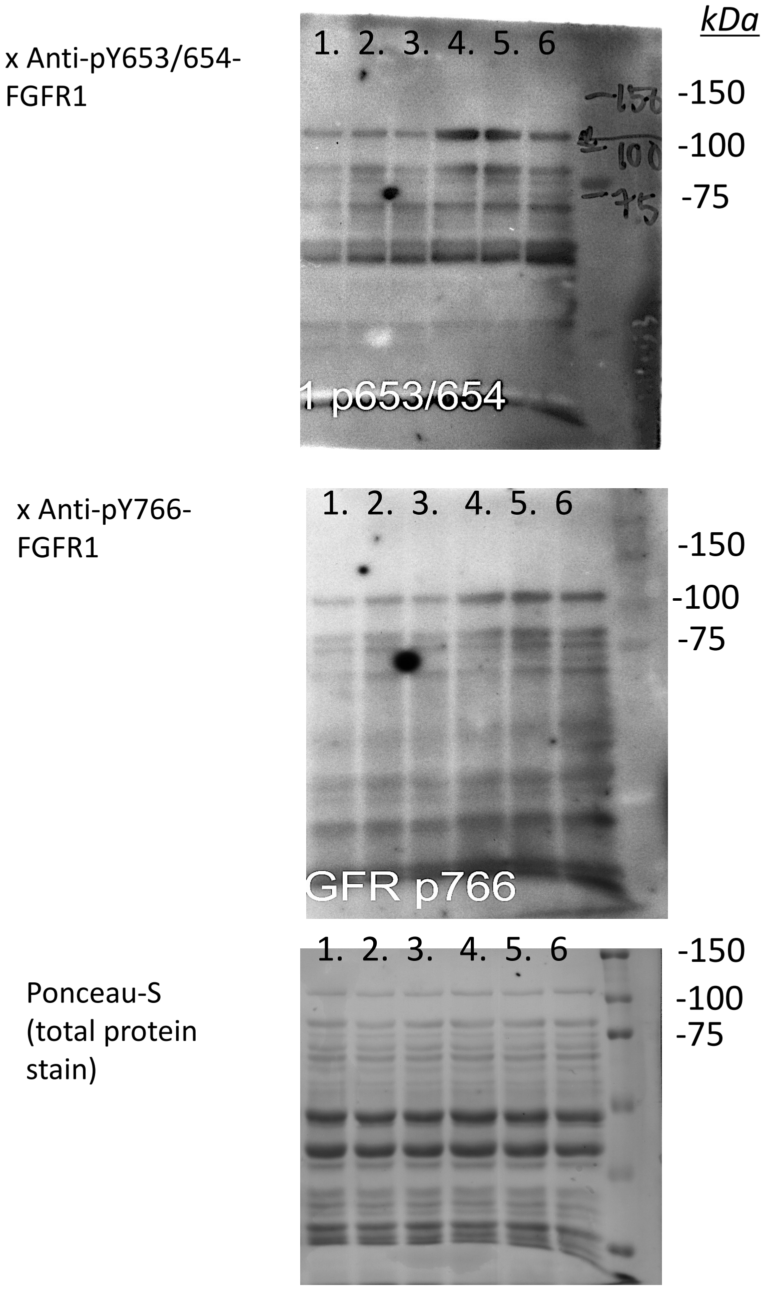

Methodology for whole heart perfusion with a Lo-FGF2 containing buffer for 15 minutes, is described in references #17, and #32 (PhD thesis of the first author). Mitochondrial samples (20 microg/lane) were analyzed in 7.5% polyacrylamide gels.

Table S1. The effect of calcium overload, Lo-FGF2, and PKCε inhibitors on cardiac (SSM) mitochondrial Optical Density (OD) at 545 nM

	Control	Lo-FGF2	Lo-FGF2 + eV1-2	Lo-FGF2 + scrambled peptide
**Calcium Retention Capacity** (nM Ca^{2+} /mg protein)	**500 ± 72**	**936 ± 5.8***	**375 ± 56**	**900 ± 13.5***
**Rate of swelling** (OD_{545} x 10^3/min)	**73.1 ± 5.6**	**75.5 ± 9.9**	**61.8 ± 5.4**	**75.2 ± 6.6**
**Amplitude of swelling** (% of maximal swelling)	**30.0 ± 0.3**	**29.3 ± 2.5**	**29.6 ± 2.3**	**32.9 ± 3.3**

Table S2. The effect of Lo-FGF2 on cardiac mitochondria (SSM) Respiration

	Rate of Respiration		RCI (State 3/ State 4)	ADP/O ratio nmol ADP/ nmol O2	Rate of Oxidative Phosphorylation (nmol ATP/min/ mg protein)
	State 3	State 4			
Control	154.3 ± 6.7	19.9 ± 1.2	7.79 ± 0.26	2.51 ± 0.05	387.3 ± 19.5
Lo-FGF2	171.3 ± 10.3	20.0 ± 2.4	8.85 ± 0.87	2.56 ± 0.04	437.9 ± 25.8

December 7, 2007

ADP + oligo + FGF-2 (50ng) + 50 mM Ca^{2+}

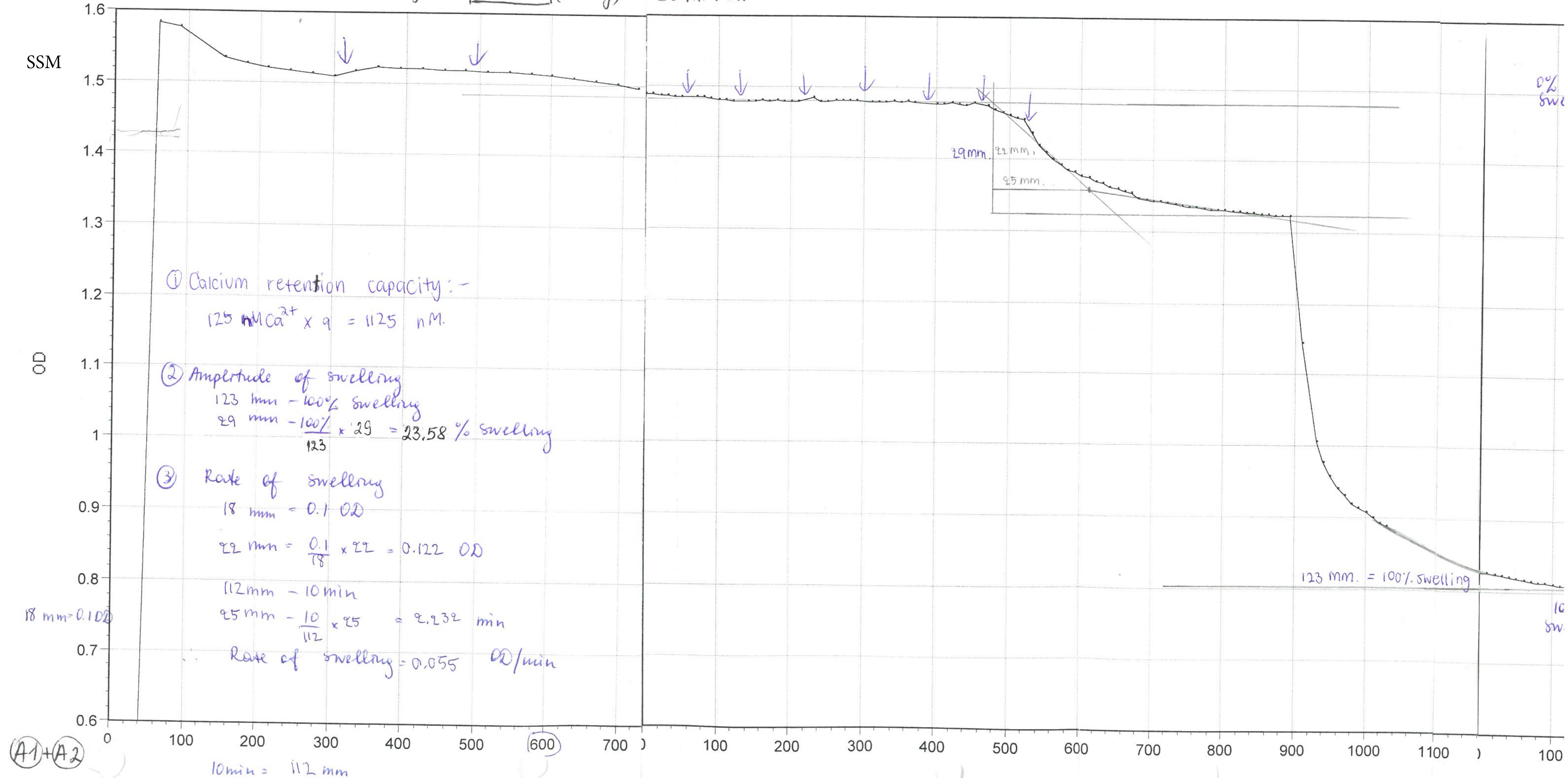

① Calcium retention capacity:-

125 nM Ca^{2+} x 9 = 1125 nM.

② Amplitude of swelling

123 mm - 100% swelling

29 mm - $\frac{100\%}{123}$ x 29 = 23.58 % swelling

③ Rate of swelling

18 mm = 0.1 OD

22 mm = $\frac{0.1}{18}$ x 22 = 0.122 OD

112mm - 10 min

25 mm - $\frac{10}{112}$ x 25 = 2.232 min

Rate of swelling = 0.055 OD/min

A1+A2

10 min = 112 mm

SSM

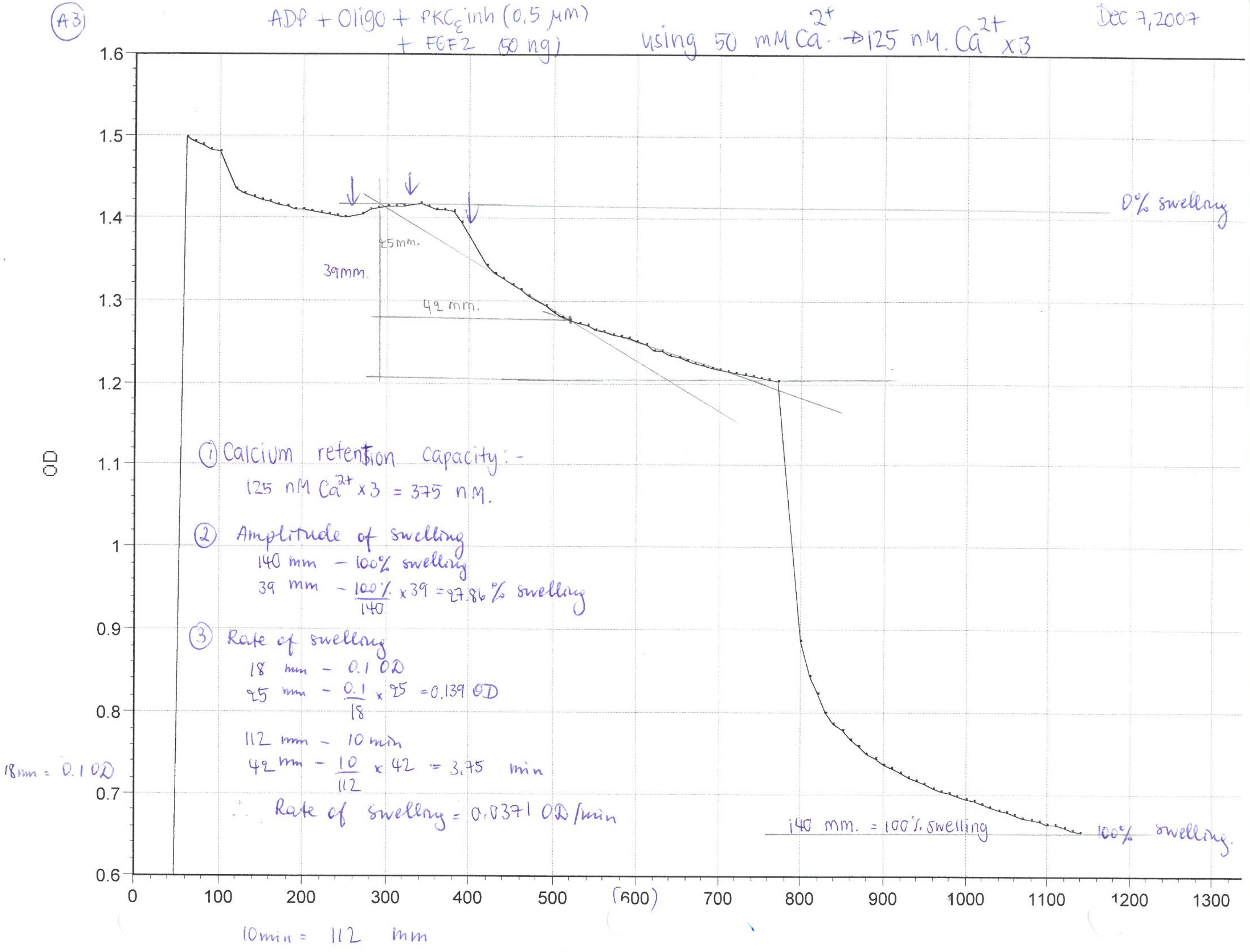

A3
ADP + Oligo + PKCε inh (0.5 μM) + FGF2 (50 ng)
using 50 mM Ca2+ → 125 nM. Ca2+ x3
Dec 7, 2007
OD
0% swelling
25mm.
39mm.
42 mm.
① Calcium retention capacity:-
125 nM Ca2+ x 3 = 375 nM.
② Amplitude of swelling
140 mm - 100% swelling
39 mm - 100% / 140 x 39 = 27.86% swelling
③ Rate of swelling
18 mm - 0.1 OD
25 mm - 0.1 / 18 x 25 = 0.139 OD
112 mm - 10 min
42 mm - 10 / 112 x 42 = 3.75 min
∴ Rate of swelling = 0.0371 OD/min
18mm = 0.1 OD
140 mm. = 100% swelling
100% swelling.
10min = 112 mm

SSM

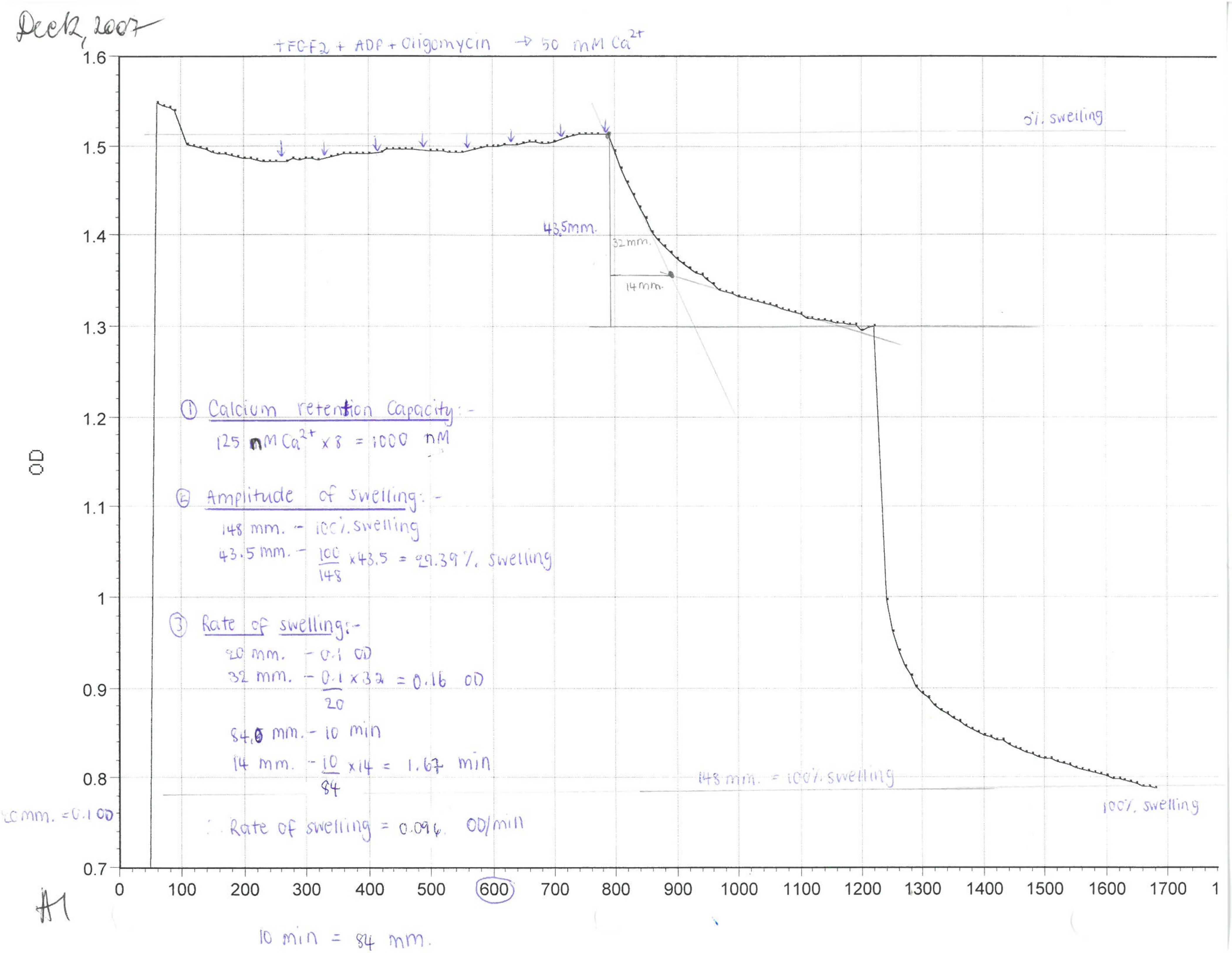
Dec 12, 2007
TFCF2 + ADP + Oligomycin → 50 mM Ca^{2+}
OD
5% swelling
43.5 mm.
32 mm.
14 mm.
① Calcium retention Capacity:-
125 nM Ca^{2+} x 8 = 1000 nM
② Amplitude of swelling:-
148 mm. - 100% swelling
43.5 mm. - 100/148 x 43.5 = 29.39% swelling
③ Rate of swelling:-
20 mm. - 0.1 OD
32 mm. - 0.1/20 x 32 = 0.16 OD
84.0 mm. - 10 min
14 mm. - 10/84 x 14 = 1.67 min
∴ Rate of swelling = 0.096 OD/min
148 mm. = 100% swelling
100% swelling
20 mm. = 0.1 OD
10 min = 84 mm.
1.6
1.5
1.4
1.3
1.2
1.1
1
0.9
0.8
0.7
0
100
200
300
400
500
600
700
800
900
1000
1100
1200
1300
1400
1500
1600
1700

SSM

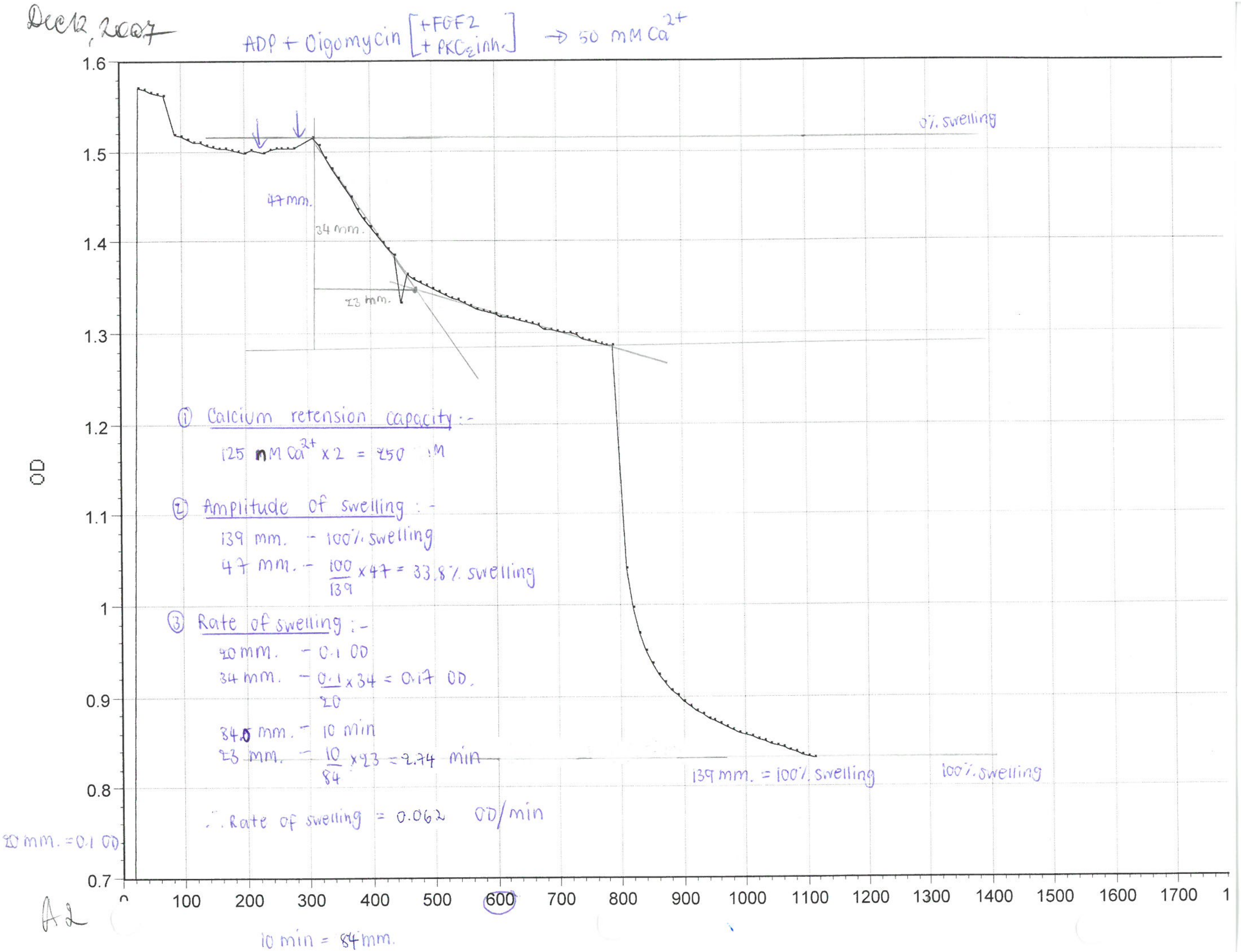
Dec 12, 2007
ADP + Oligomycin [+FGF2 + PKCε inh.] → 50 mM Ca^{2+}
0% swelling
47 mm.
34 mm.
23 mm.
① Calcium retension capacity :-
125 nM Ca^{2+} x 2 = 250 nM
② Amplitude of swelling :-
139 mm. - 100% swelling
47 mm. - 100/139 x 47 = 33.8% swelling
③ Rate of swelling :-
20 mm. - 0.1 OD
34 mm. - 0.1/20 x 34 = 0.17 OD.
84 mm. - 10 min
23 mm. - 10/84 x 23 = 2.74 min
∴ Rate of swelling = 0.062 OD/min
139 mm. = 100% swelling
100% swelling
20 mm. = 0.1 OD
A2
10 min = 84 mm.
OD
1.6
1.5
1.4
1.3
1.2
1.1
1
0.9
0.8
0.7
0
100
200
300
400
500
600
700
800
900
1000
1100
1200
1300
1400
1500
1600
1700

SSM

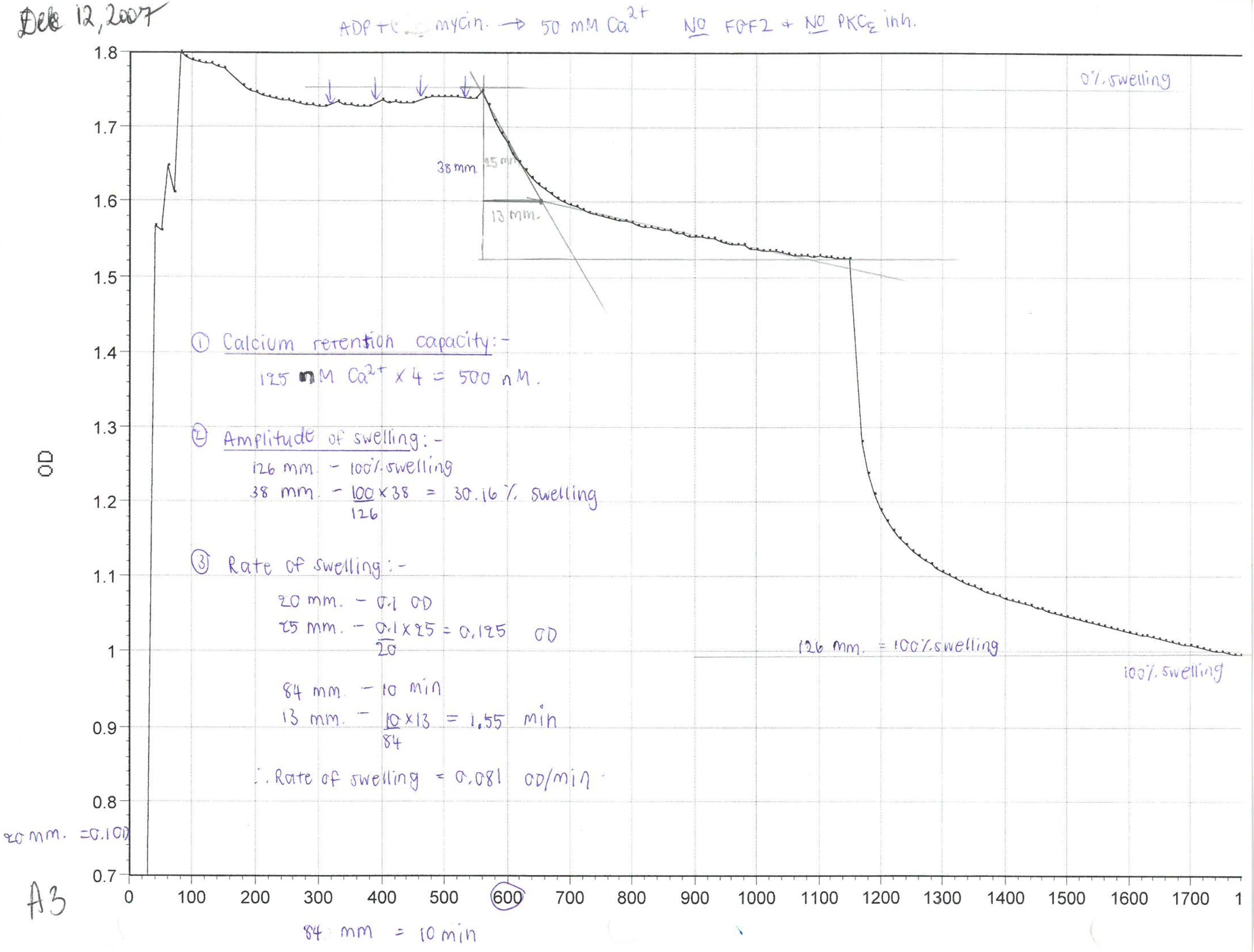
Dec 12, 2007
ADP + C[illegible]mycin → 50 mM Ca2+ NO FCF2 + NO PKCε inh.
0% swelling
38 mm
25 mm
13 mm.
① Calcium retention capacity:-
125 nM Ca2+ x 4 = 500 nM.
② Amplitude of swelling:-
126 mm - 100% swelling
38 mm - 100/126 x 38 = 30.16 % swelling
③ Rate of swelling:-
20 mm. - 0.1 OD
25 mm. - 0.1/20 x 25 = 0.125 OD
84 mm - 10 min
13 mm. - 10/84 x 13 = 1.55 min
∴ Rate of swelling = 0.081 OD/min
126 mm. = 100% swelling
100% swelling
20 mm. = 0.100
OD
1.8
1.7
1.6
1.5
1.4
1.3
1.2
1.1
1
0.9
0.8
0.7
0
100
200
300
400
500
600
700
800
900
1000
1100
1200
1300
1400
1500
1600
1700
1
A3
84 mm = 10 min

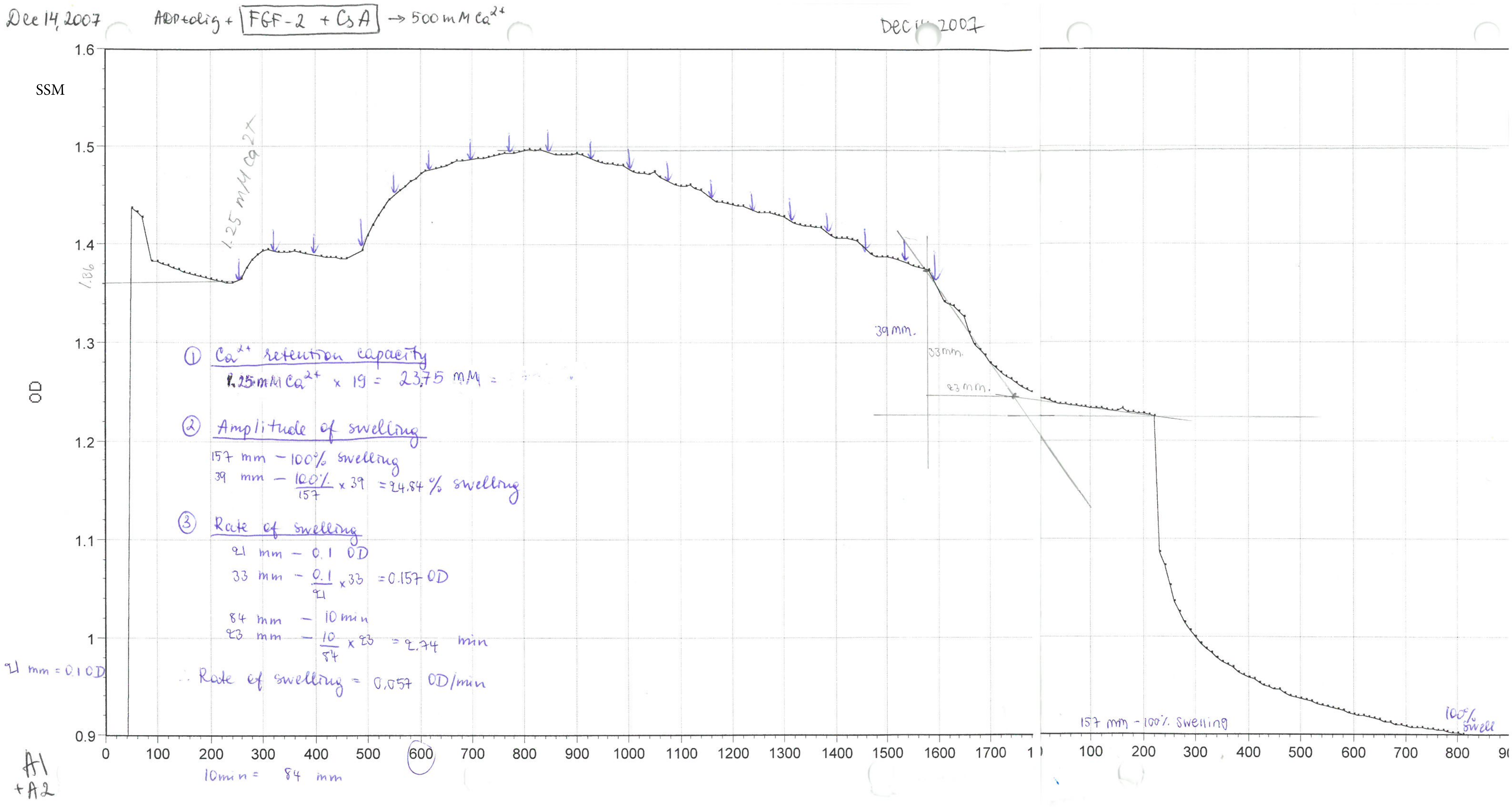

Dec 14, 2007
ADP+olig + FGF-2 + CsA → 500 mM Ca^{2+}
Dec 14 2007
SSM
OD
1.25 mM Ca^{2+}
1.36
1.6
1.5
1.4
1.3
1.2
1.1
1
0.9
0
100
200
300
400
500
600
700
800
900
1000
1100
1200
1300
1400
1500
1600
1700
39 mm.
33 mm.
23 mm.
① Ca^{2+} retention capacity
1.25 mM Ca^{2+} x 19 = 23.75 mM = [illegible]
② Amplitude of swelling
157 mm – 100% swelling
39 mm – 100%/157 x 39 = 24.84 % swelling
③ Rate of swelling
21 mm – 0.1 OD
33 mm – 0.1/21 x 33 = 0.157 OD
84 mm – 10 min
23 mm – 10/84 x 23 = 2.74 min
∴ Rate of swelling = 0.057 OD/min
21 mm = 0.1 OD
10 min = 84 mm
A1 + A2
157 mm – 100% Swelling
100% swell

[illegible],2007 ADP+olig + FGF-2+ PKCε inhib + CsA → 500 mM Ca^{2+} SSM Dec 14, 2007

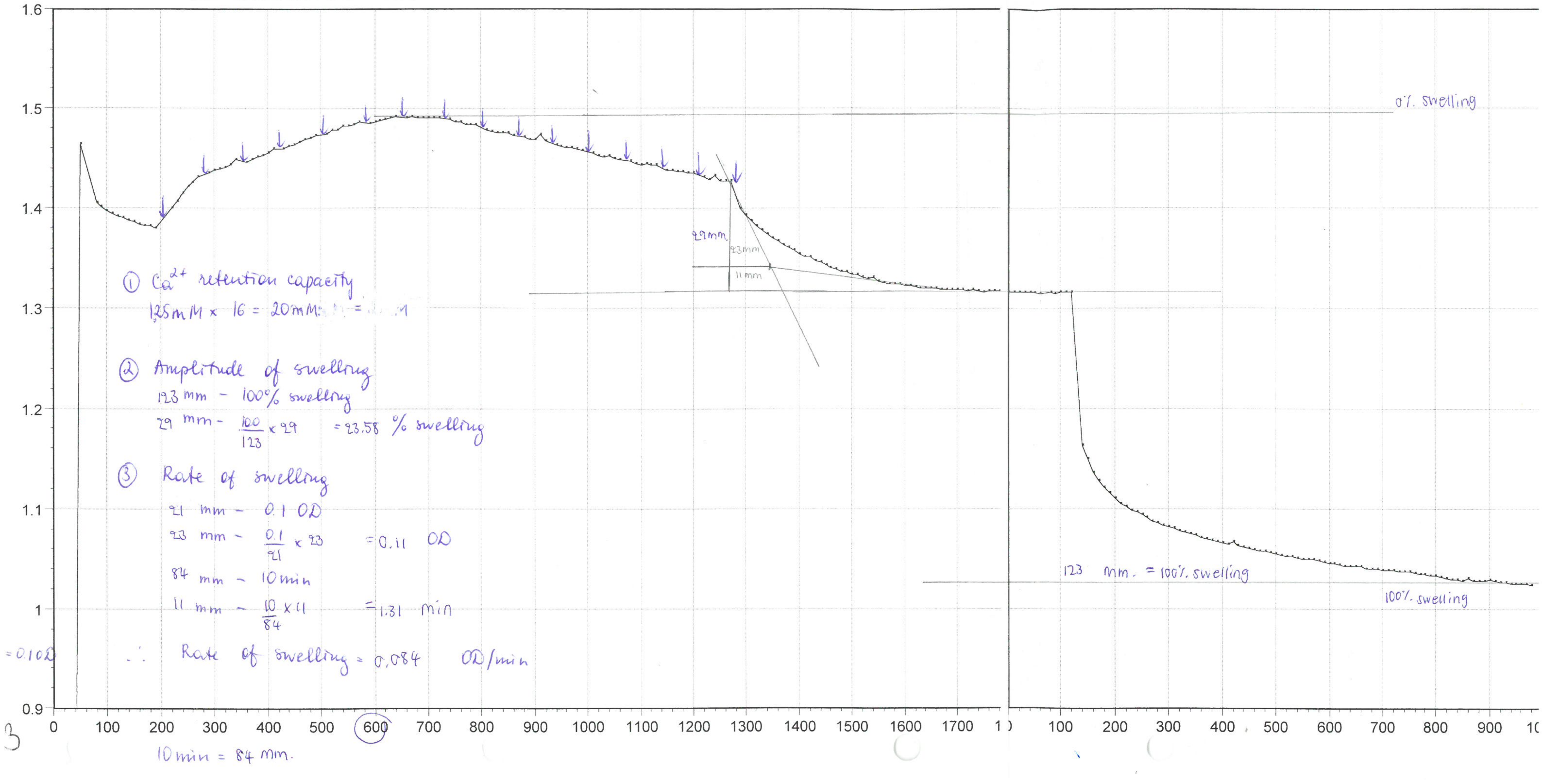

SSM

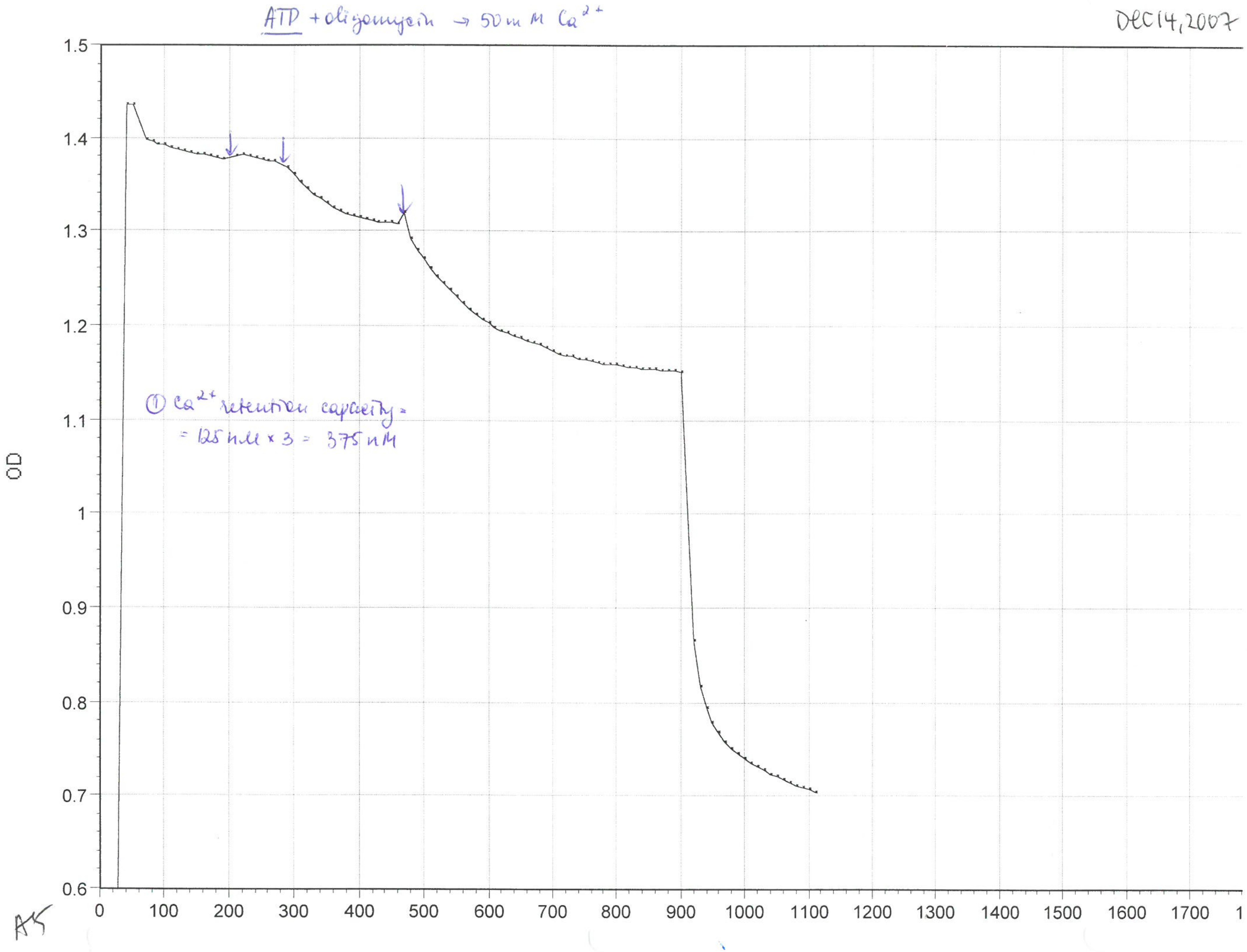
ATP + oligomycin → 50 mM Ca^{2+}
DEC 14, 2007
OD
1.5
1.4
1.3
1.2
1.1
1
0.9
0.8
0.7
0.6
0
100
200
300
400
500
600
700
800
900
1000
1100
1200
1300
1400
1500
1600
1700
① Ca^{2+} retention capacity =
= 125 nM × 3 = 375 nM
AS

ATP + olig + FGF2 → 50 mM Ca^{2+}

Dec 14, 2007

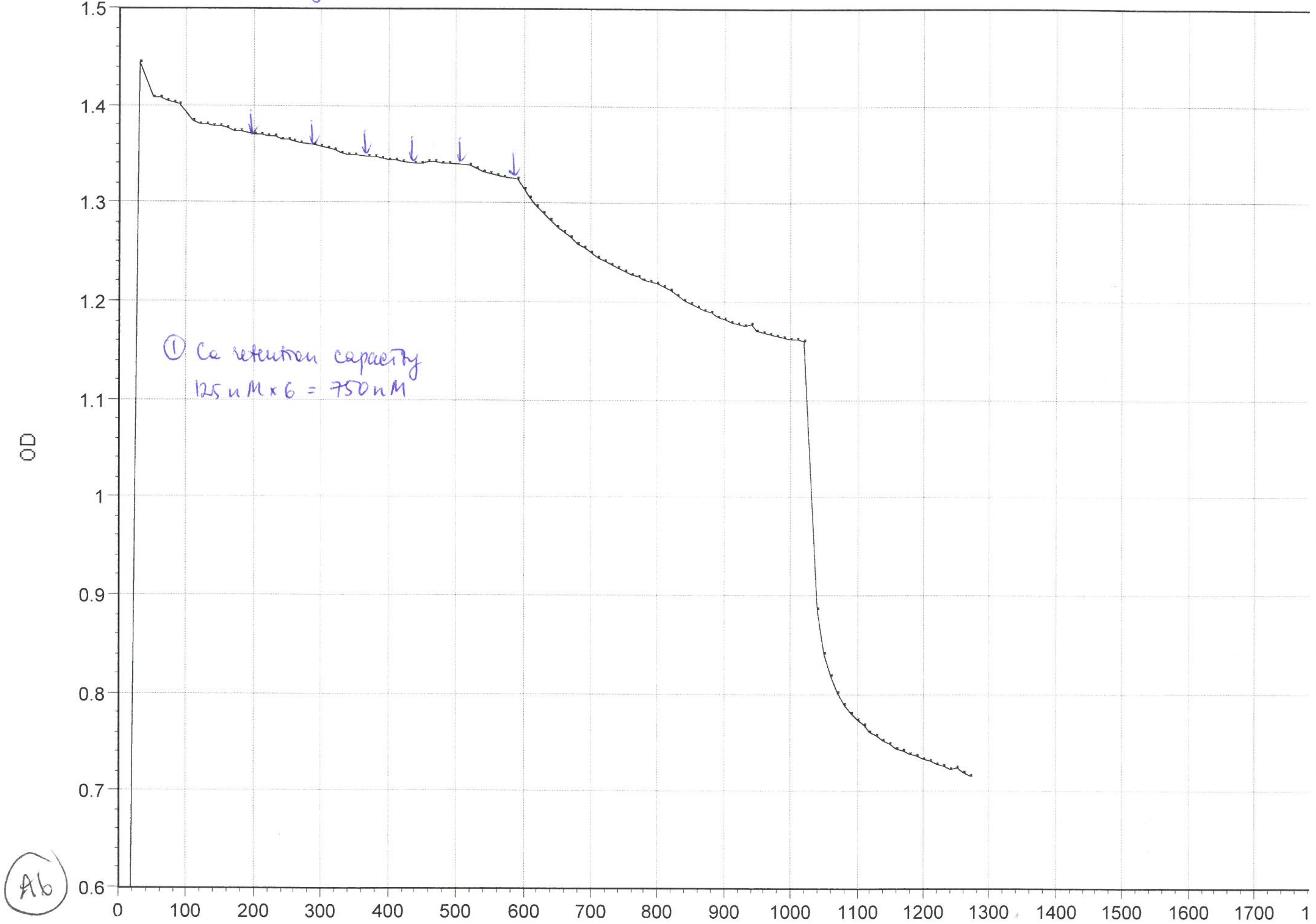

SSM

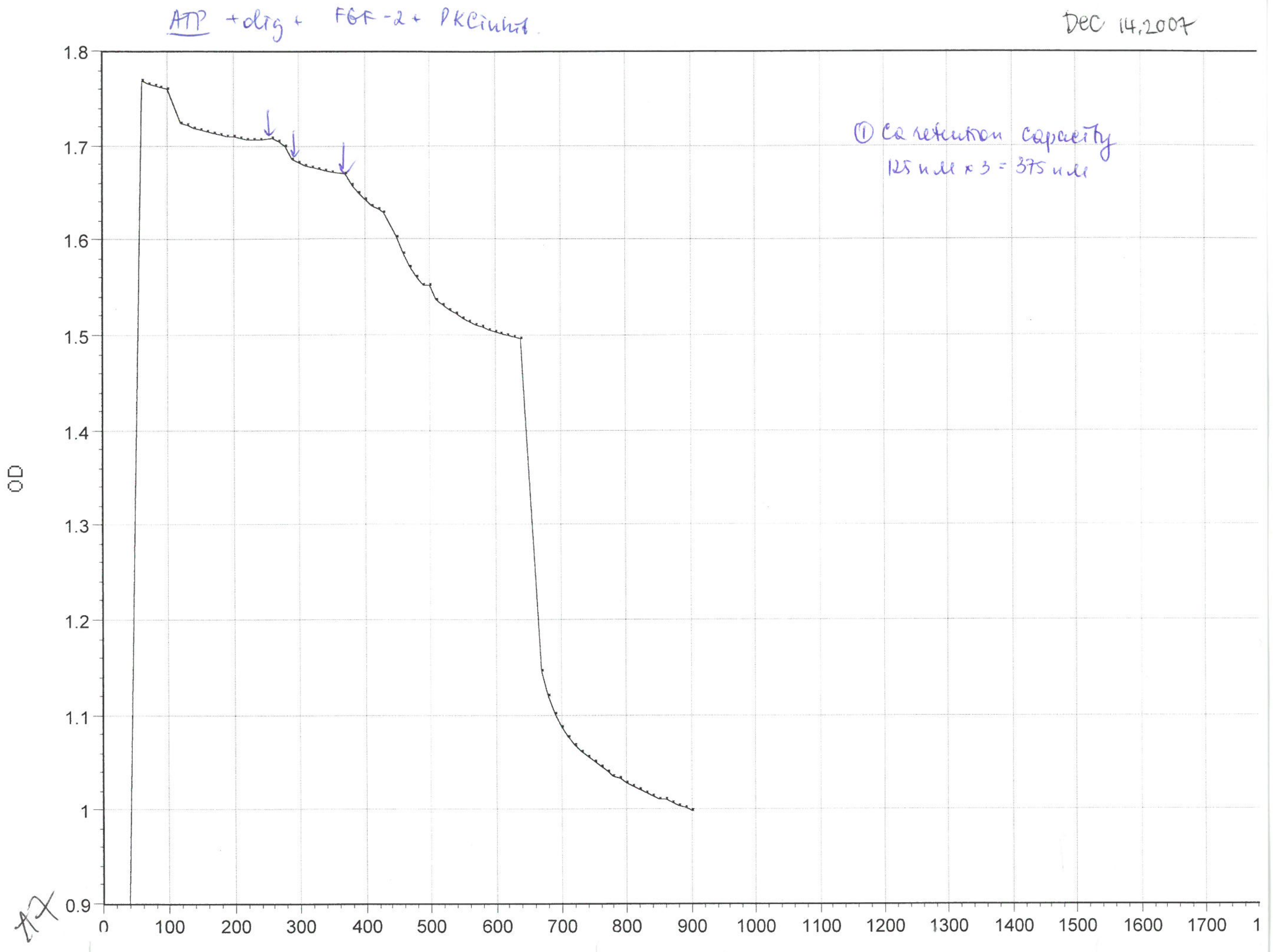
ATP + dig + FGF-2 + PKCinhit
Dec 14, 2007
① Ca retention capacity
125 nM × 3 = 375 nM
OD
1.8
1.7
1.6
1.5
1.4
1.3
1.2
1.1
1
0.9
0
100
200
300
400
500
600
700
800
900
1000
1100
1200
1300
1400
1500
1600
1700
1
AX

SSM

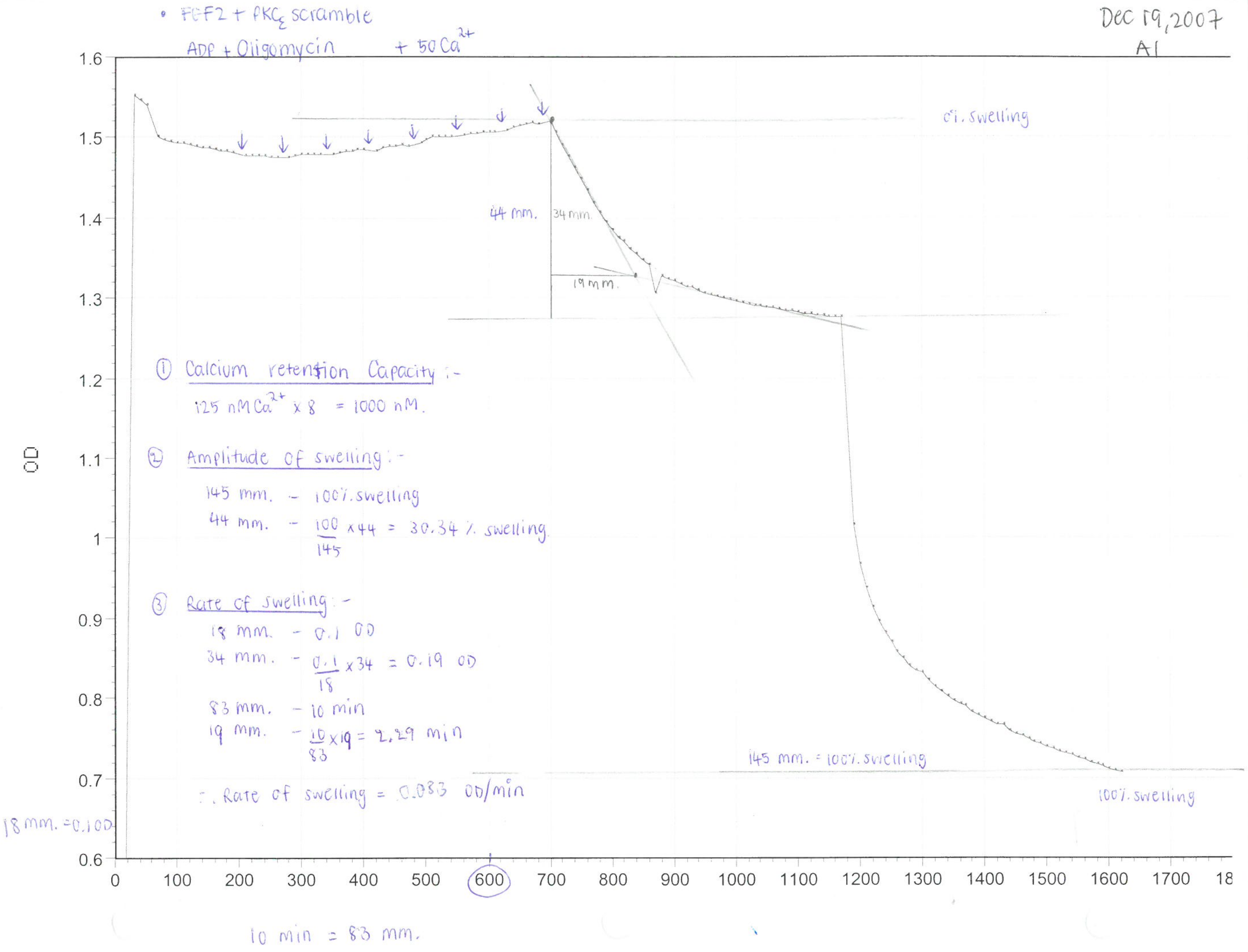

• FGF2 + PKCε scramble
ADP + Oligomycin + 50 Ca^{2+}
Dec 19, 2007
A1
OD
0.6
0.7
0.8
0.9
1
1.1
1.2
1.3
1.4
1.5
1.6
0
100
200
300
400
500
600
700
800
900
1000
1100
1200
1300
1400
1500
1600
1700
18
0% swelling
44 mm.
34 mm.
19 mm.
① Calcium retention Capacity :-
125 nM Ca^{2+} x 8 = 1000 nM.
② Amplitude of swelling :-
145 mm. - 100% swelling
44 mm. - 100/145 x 44 = 30.34 % swelling
③ Rate of swelling :-
18 mm. - 0.1 OD
34 mm. - 0.1/18 x 34 = 0.19 OD
83 mm. - 10 min
19 mm. - 10/83 x 19 = 2.29 min
∴ Rate of swelling = 0.083 OD/min
145 mm. = 100% swelling
100% swelling
18 mm. = 0.1 OD
10 min = 83 mm.

SSM

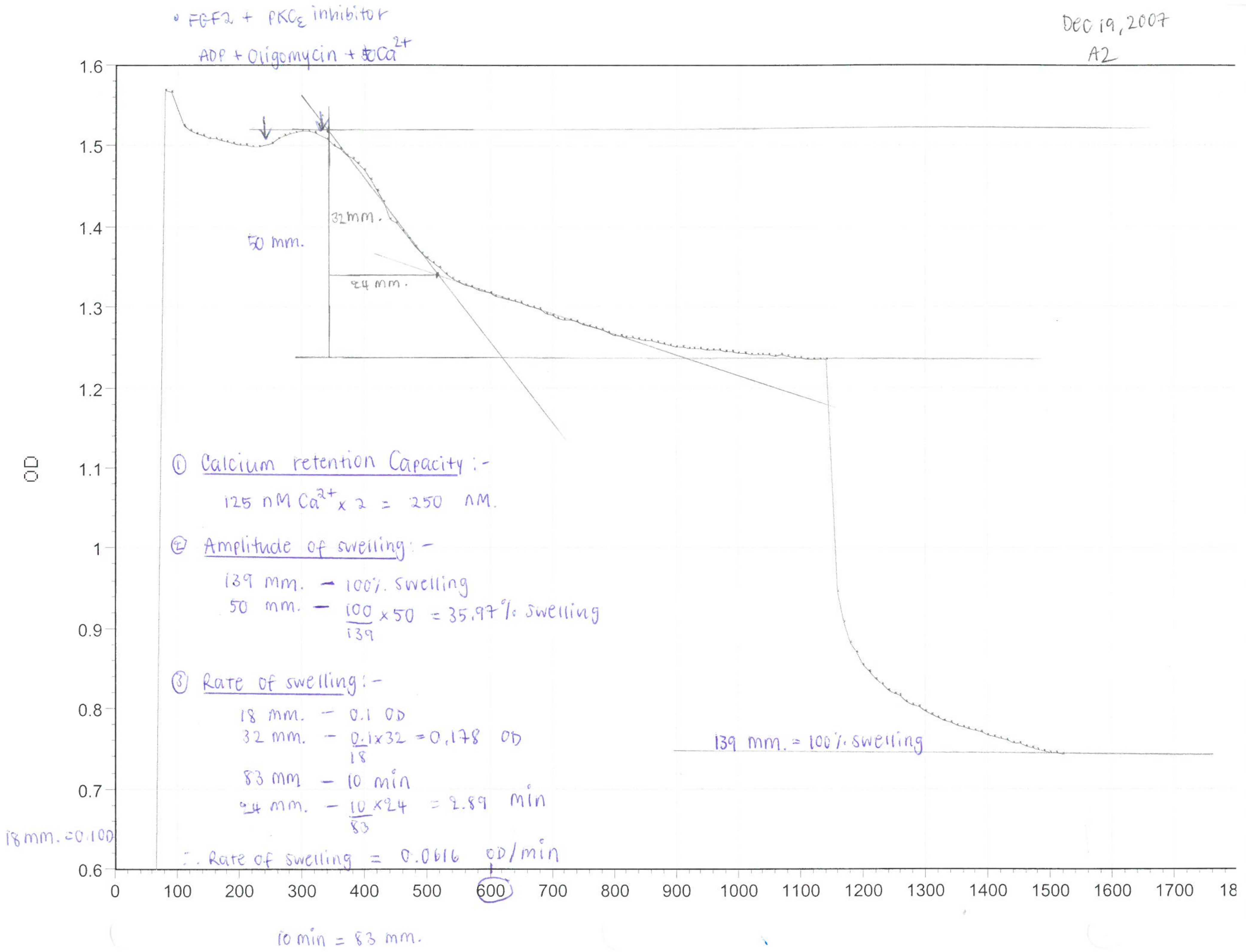

• FGF2 + PKCε inhibitor
ADP + Oligomycin + Ca2+
Dec 19, 2007
A2
OD
32 mm.
50 mm.
24 mm.
① Calcium retention Capacity :-
125 nM Ca2+ x 2 = 250 nM.
② Amplitude of swelling :-
139 mm. — 100% swelling
50 mm. — 100/139 x 50 = 35.97% swelling
③ Rate of swelling :-
18 mm. — 0.1 OD
32 mm. — 0.1x32/18 = 0.178 OD
83 mm — 10 min
24 mm. — 10x24/83 = 2.89 min
∴ Rate of swelling = 0.0616 OD/min
139 mm. = 100% swelling
18 mm. = 0.100
10 min = 83 mm.

SSM

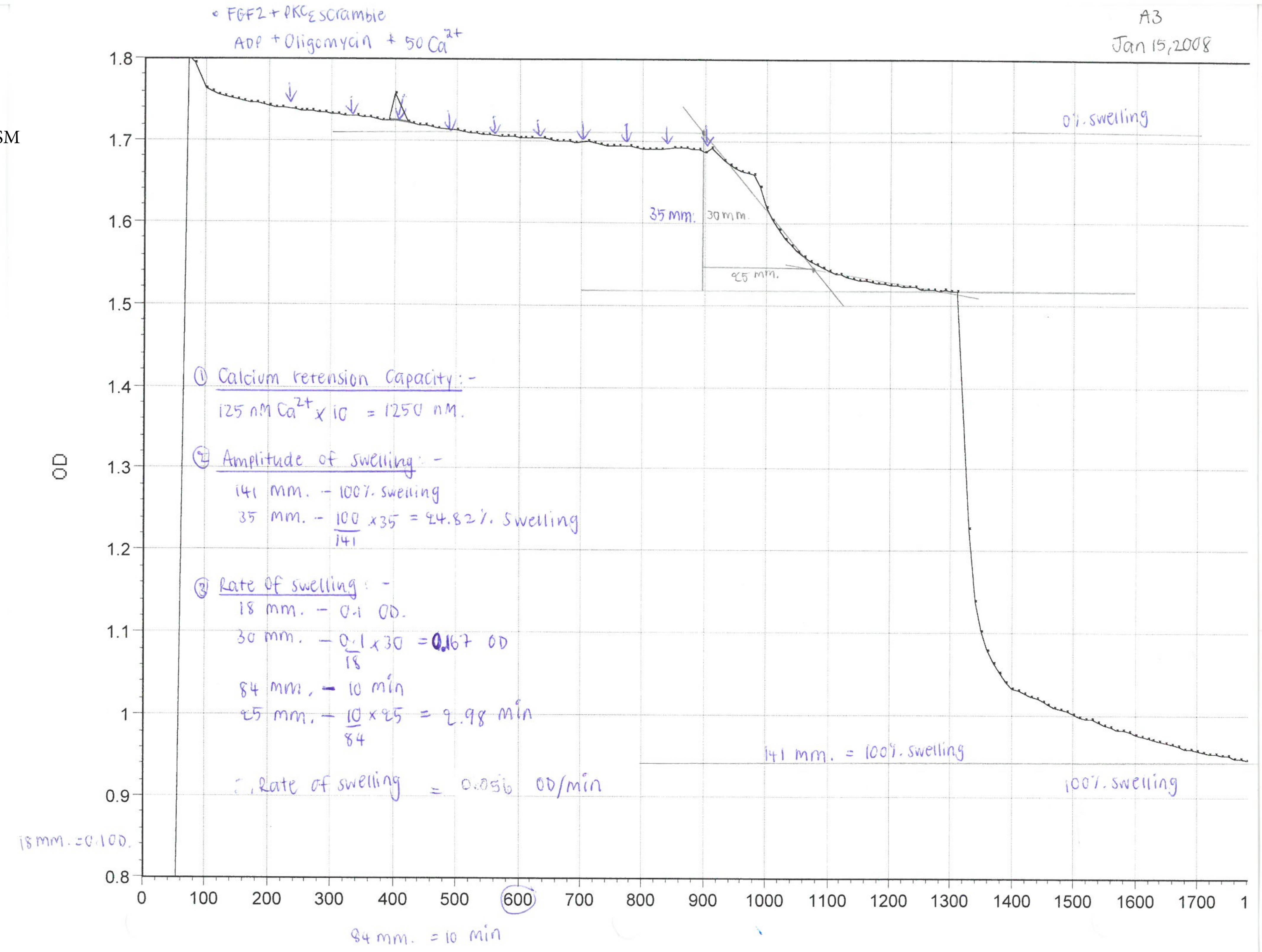

• FGF2 + PKCε scramble
ADP + Oligomycin + 50 Ca^{2+}
A3
Jan 15, 2008
OD
0 100 200 300 400 500 600 700 800 900 1000 1100 1200 1300 1400 1500 1600 1700 1
0.8 0.9 1 1.1 1.2 1.3 1.4 1.5 1.6 1.7 1.8
0% swelling
35 mm.
30 mm.
25 mm.
① Calcium retension capacity :-
125 nM Ca^{2+} x 10 = 1250 nM.
② Amplitude of swelling :-
141 mm. - 100% swelling
35 mm. - $\frac{100}{141}$ x 35 = 24.82% swelling
③ Rate of swelling :-
18 mm. - 0.1 OD.
30 mm. - $\frac{0.1}{18}$ x 30 = 0.167 OD
84 mm. - 10 min
25 mm. - $\frac{10}{84}$ x 25 = 2.98 min
∴ Rate of swelling = 0.056 OD/min
141 mm. = 100% swelling
100% swelling
18 mm. = 0.1 OD.
84 mm. = 10 min

SSM

• FGF2
• ADP + Oligomycin + 50 Ca^{2+}

A1
Jan 15, 2008

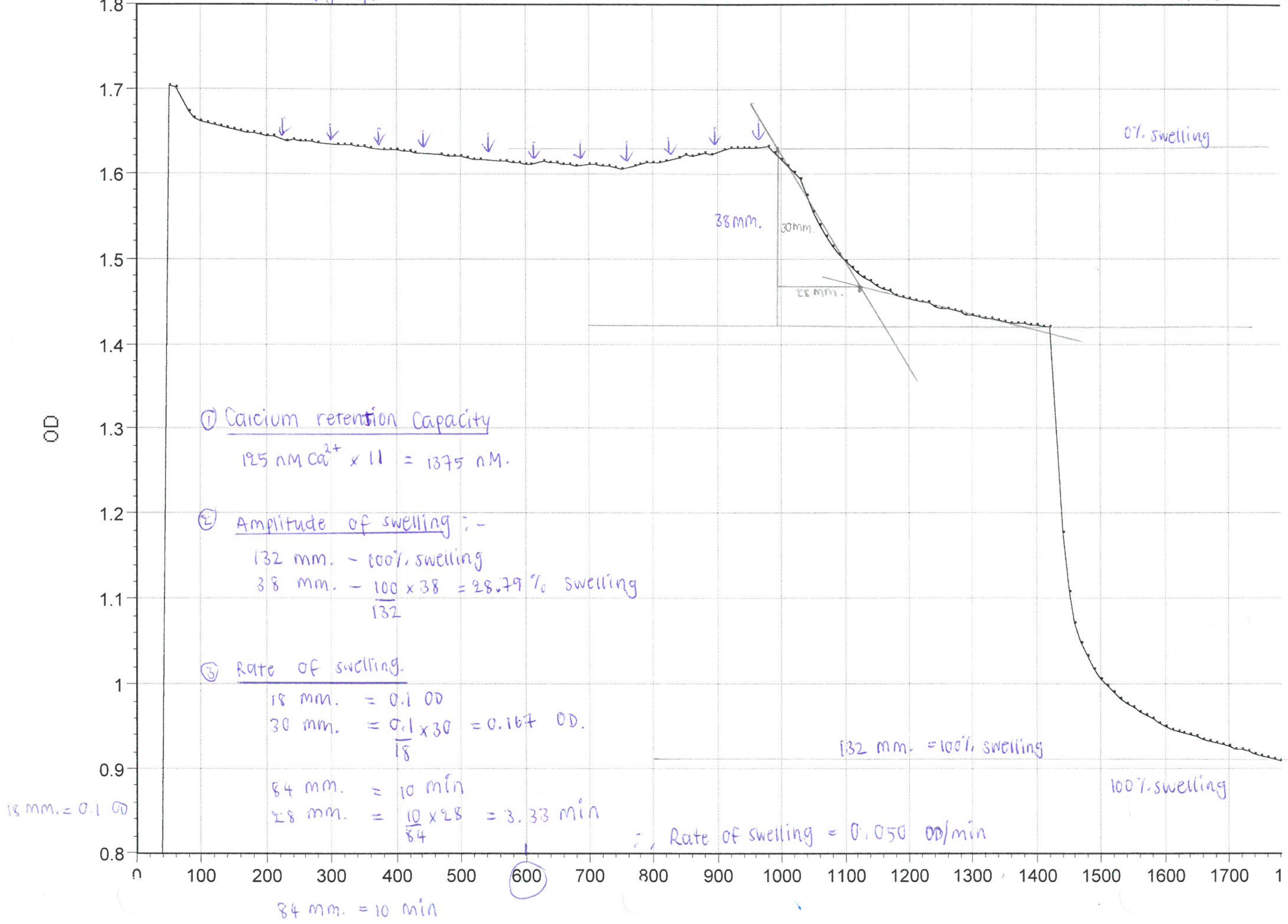

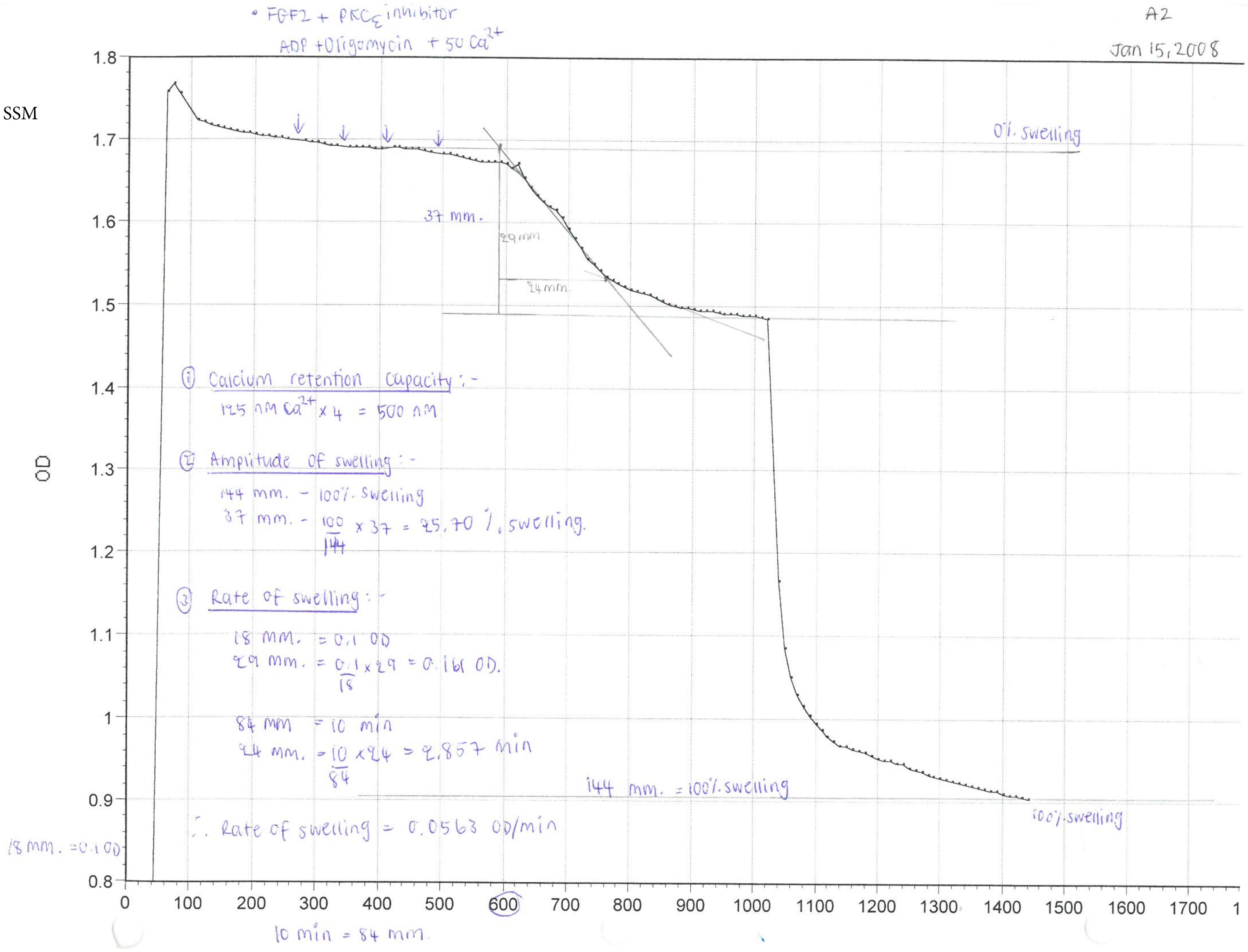

• FGF2 + PKCε inhibitor
ADP + Oligomycin + 50 Ca^{2+}
A2
Jan 15, 2008
SSM
OD
1.8
1.7
1.6
1.5
1.4
1.3
1.2
1.1
1
0.9
0.8
0 100 200 300 400 500 600 700 800 900 1000 1100 1200 1300 1400 1500 1600 1700 1
0% swelling
37 mm.
29 mm
24 mm.
① Calcium retention capacity :-
125 nM Ca^{2+} x 4 = 500 nM
② Amplitude of swelling :-
144 mm. - 100% swelling
37 mm. - $\frac{100}{144}$ x 37 = 25.70 % swelling.
③ Rate of swelling :-
18 mm. = 0.1 OD
29 mm. = $\frac{0.1}{18}$ x 29 = 0.161 OD.
84 mm = 10 min
24 mm. = $\frac{10}{84}$ x 24 = 2.857 min
144 mm. = 100% swelling
∴ Rate of swelling = 0.0563 OD/min
100% swelling
18 mm. = 0.1 OD
10 min = 84 mm.

SSM

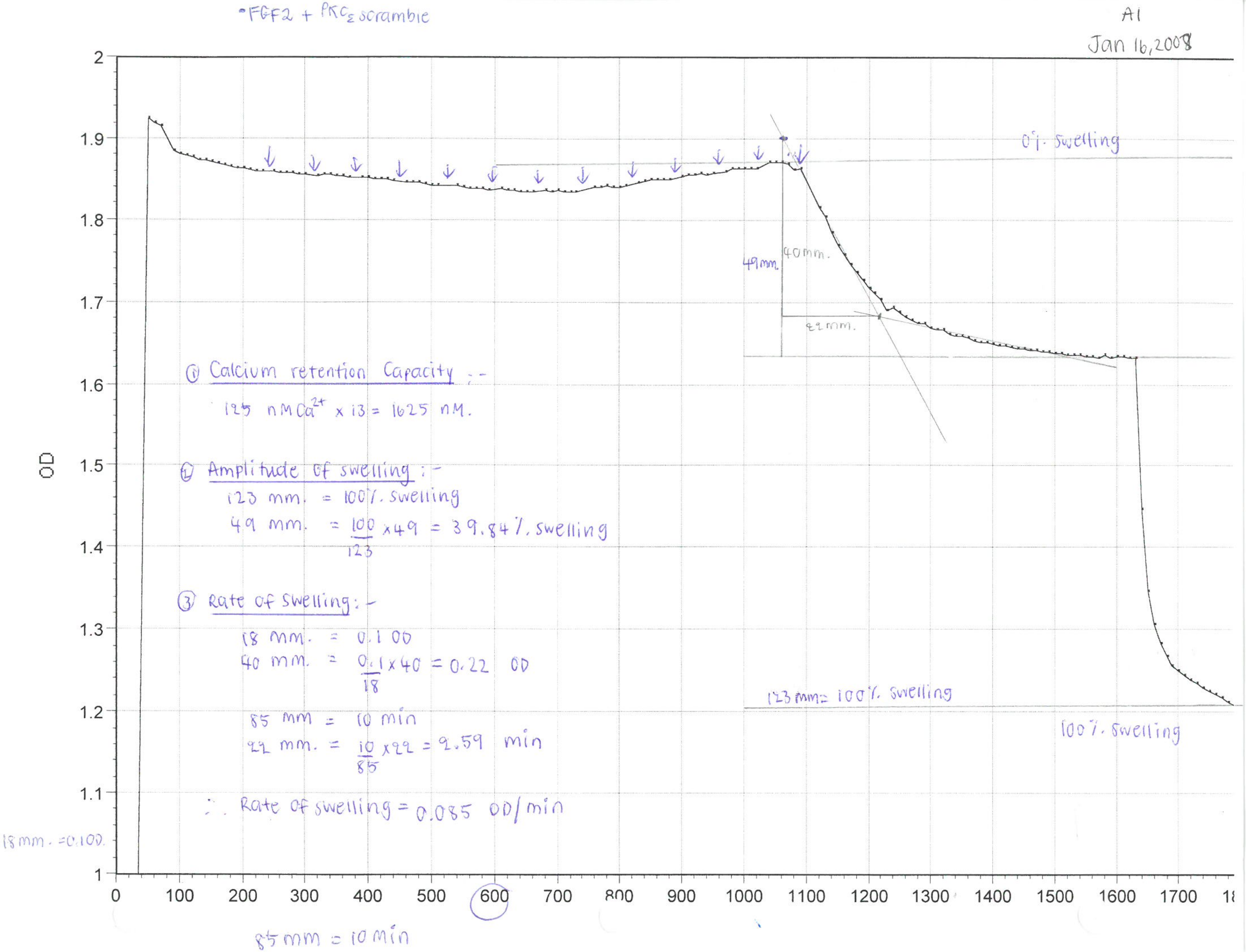

*FGF2 + PKCε scramble
A1
Jan 16, 2008
OD
2
1.9
1.8
1.7
1.6
1.5
1.4
1.3
1.2
1.1
1
0 100 200 300 400 500 600 700 800 900 1000 1100 1200 1300 1400 1500 1600 1700 18
0% swelling
49mm.
40mm.
22mm.
① Calcium retention Capacity :-
125 nM Ca2+ x 13 = 1625 nM.
② Amplitude of swelling :-
123 mm. = 100% swelling
49 mm. = 100/123 x49 = 39.84% swelling
③ Rate of Swelling :-
18 mm. = 0.1 OD
40 mm. = 0.1/18 x40 = 0.22 OD
85 mm = 10 min
22 mm. = 10/85 x22 = 2.59 min
∴ Rate of swelling = 0.085 OD/min
123 mm = 100% swelling
100% swelling
18mm. = 0.1 OD.
85 mm = 10 min

SSM

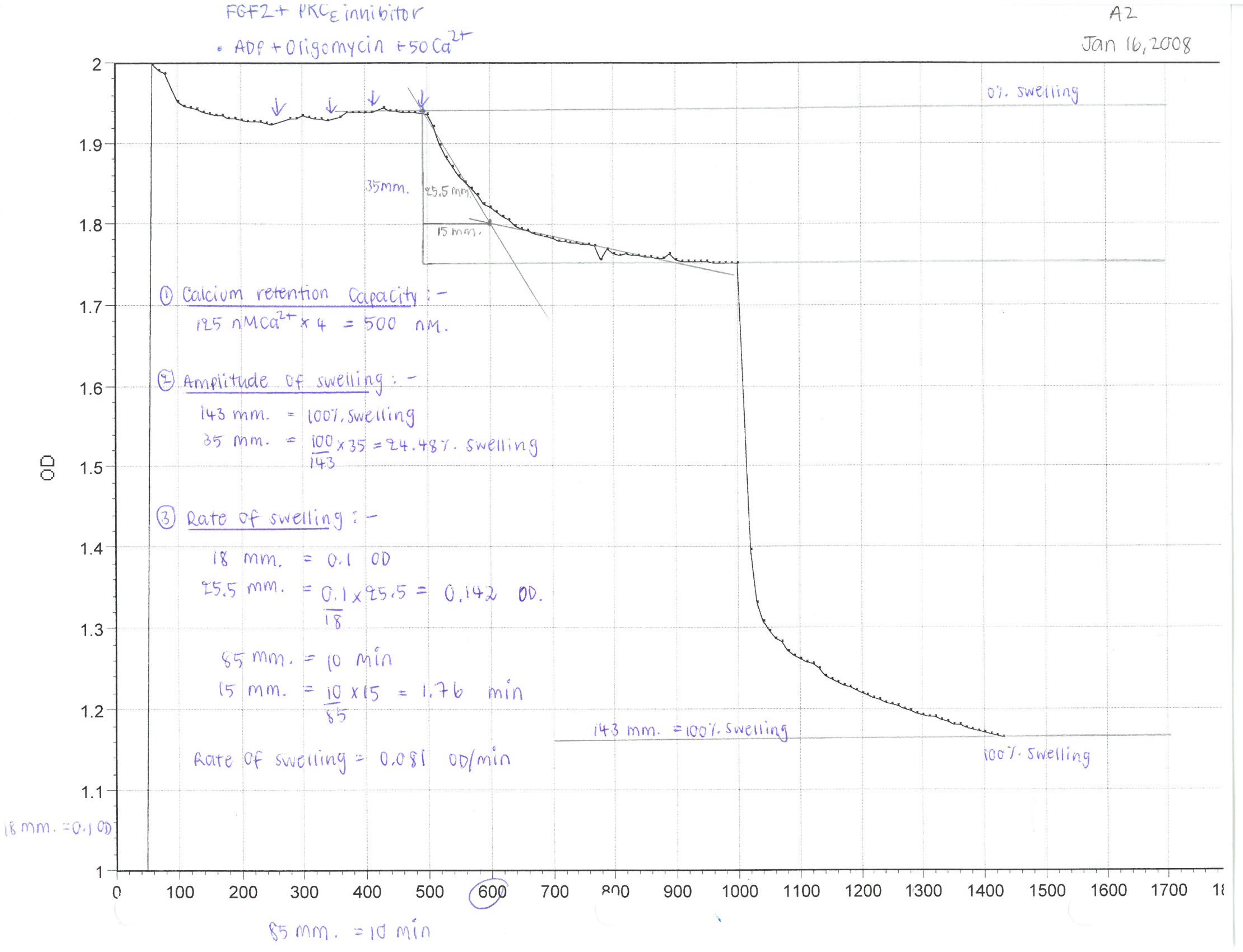

FGF2+ PKCε inhibitor
• ADP + Oligomycin +50 Ca2+
A2
Jan 16, 2008
0% swelling
35mm.
25.5 mm.
15 mm.
① Calcium retention Capacity :-
125 nMCa2+ x 4 = 500 nM.
② Amplitude of swelling :-
143 mm. = 100% swelling
35 mm. = 100/143 x 35 = 24.48% swelling
③ Rate of swelling :-
18 mm. = 0.1 OD
25.5 mm. = 0.1/18 x 25.5 = 0.142 OD.
85 mm. = 10 min
15 mm. = 10/85 x 15 = 1.76 min
Rate of swelling = 0.081 OD/min
143 mm. = 100% swelling
100% swelling
18 mm. = 0.1 OD
85 mm. = 10 min
OD
2
1.9
1.8
1.7
1.6
1.5
1.4
1.3
1.2
1.1
1
0
100
200
300
400
500
600
700
800
900
1000
1100
1200
1300
1400
1500
1600
1700

SSM

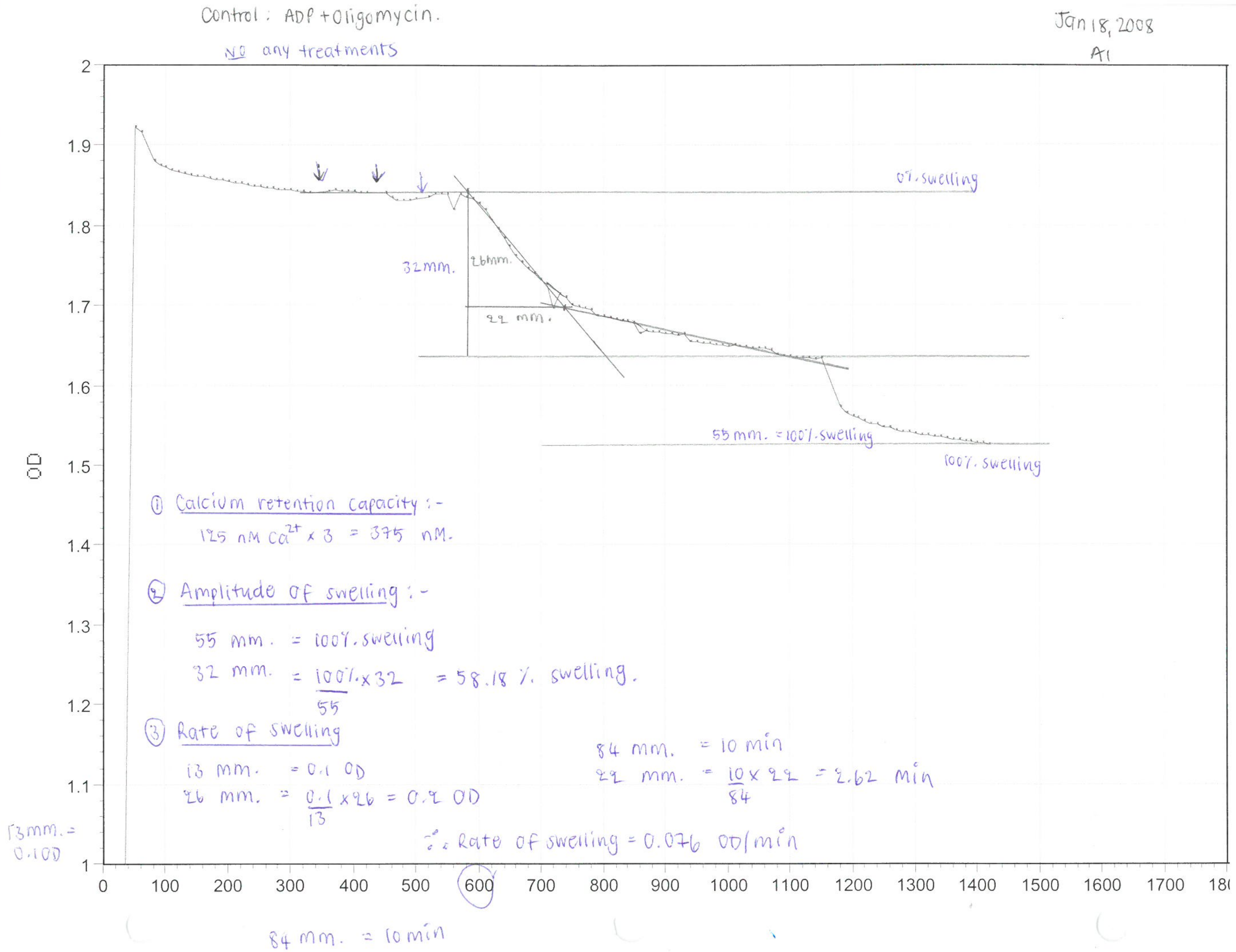

Control : ADP + Oligomycin.
No any treatments
Jan 18, 2008
A1
0% swelling
32 mm.
26 mm.
22 mm.
55 mm. = 100% swelling
100% swelling
OD
① Calcium retention capacity :-
125 nM Ca2+ x 3 = 375 nM.
② Amplitude of swelling :-
55 mm. = 100% swelling
32 mm. = 100% x 32 / 55 = 58.18 % swelling.
③ Rate of swelling
13 mm. = 0.1 OD
26 mm. = 0.1/13 x 26 = 0.2 OD
84 mm. = 10 min
22 mm. = 10/84 x 22 = 2.62 min
∴ Rate of swelling = 0.076 OD/min
13 mm. = 0.1 OD
84 mm. = 10 min

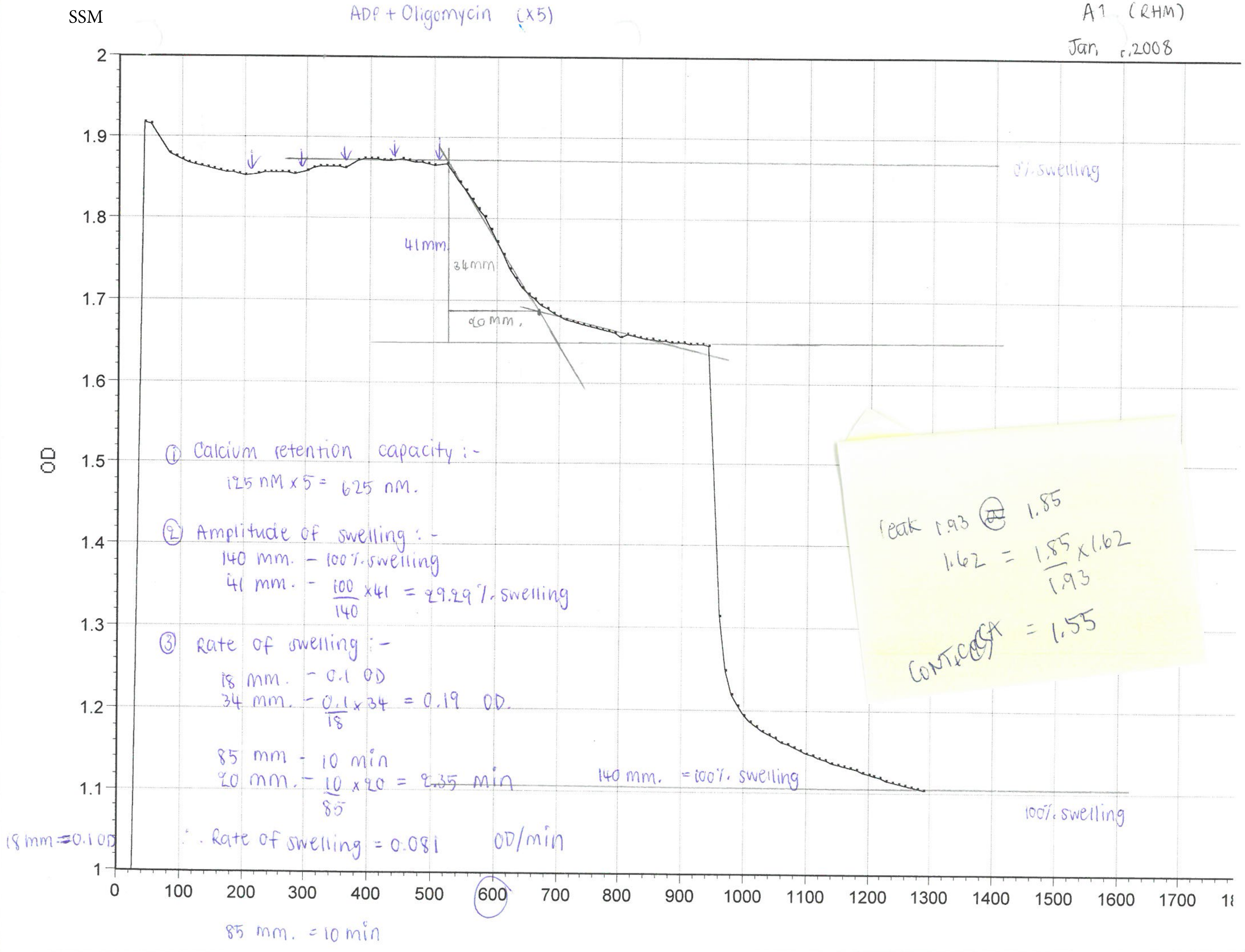

SSM
ADP + Oligomycin (x5)
A1 (RHM)
Jan. [illegible] 2008
OD
0
100
200
300
400
500
600
700
800
900
1000
1100
1200
1300
1400
1500
1600
1700
1
1.1
1.2
1.3
1.4
1.5
1.6
1.7
1.8
1.9
2
0% swelling
41mm
34mm
20mm
① Calcium retention capacity :-
125 nM x 5 = 625 nM.
② Amplitude of swelling :-
140 mm. - 100% swelling
41 mm. - 100/140 x 41 = 29.29% swelling
③ Rate of swelling :-
18 mm. - 0.1 OD
34 mm. - 0.1/18 x 34 = 0.19 OD.
85 mm - 10 min
20 mm. - 10/85 x 20 = 2.35 min
140 mm. = 100% swelling
100% swelling
18 mm = 0.1 OD
∴ Rate of swelling = 0.081 OD/min
85 mm. = 10 min
Peak 1.93 [illegible] 1.85
1.62 = 1.85/1.93 x 1.62
CONT (CAGA) = 1.55

SSM

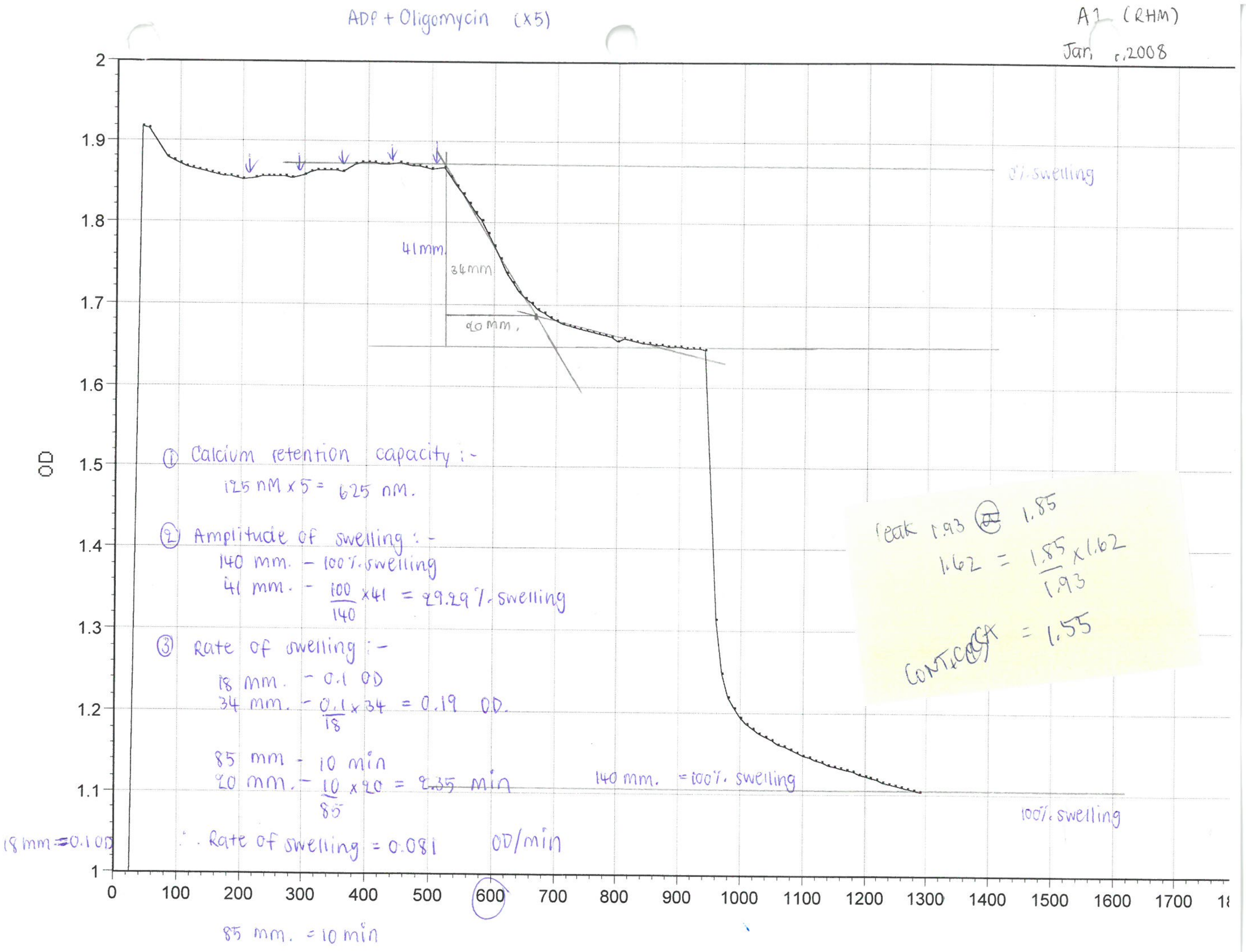

ADP + Oligomycin (x5)
A1 (RHM)
Jan, 2008
OD
2
1.9
1.8
1.7
1.6
1.5
1.4
1.3
1.2
1.1
1
0
100
200
300
400
500
600
700
800
900
1000
1100
1200
1300
1400
1500
1600
1700
0% swelling
41mm
34mm
20mm
① Calcium retention capacity :-
125 nM x 5 = 625 nM.
② Amplitude of swelling :-
140 mm. – 100% swelling
41 mm. – 100/140 x41 = 29.29% swelling
③ Rate of swelling :-
18 mm. – 0.1 OD
34 mm. – 0.1/18 x 34 = 0.19 OD.
85 mm – 10 min
20 mm. – 10/85 x 20 = 2.35 min
∴ Rate of swelling = 0.081 OD/min
18 mm = 0.1 OD
140 mm. = 100% swelling
100% swelling
85 mm. = 10 min
Peak 1.93 = 1.85
1.62 = 1.85/1.93 x 1.62
CONT.CA = 1.55

SSM

ADP + Oligomycin + FCF2 (x9)

A2 (RHM)

Jan 2[illegible], [illegible]008

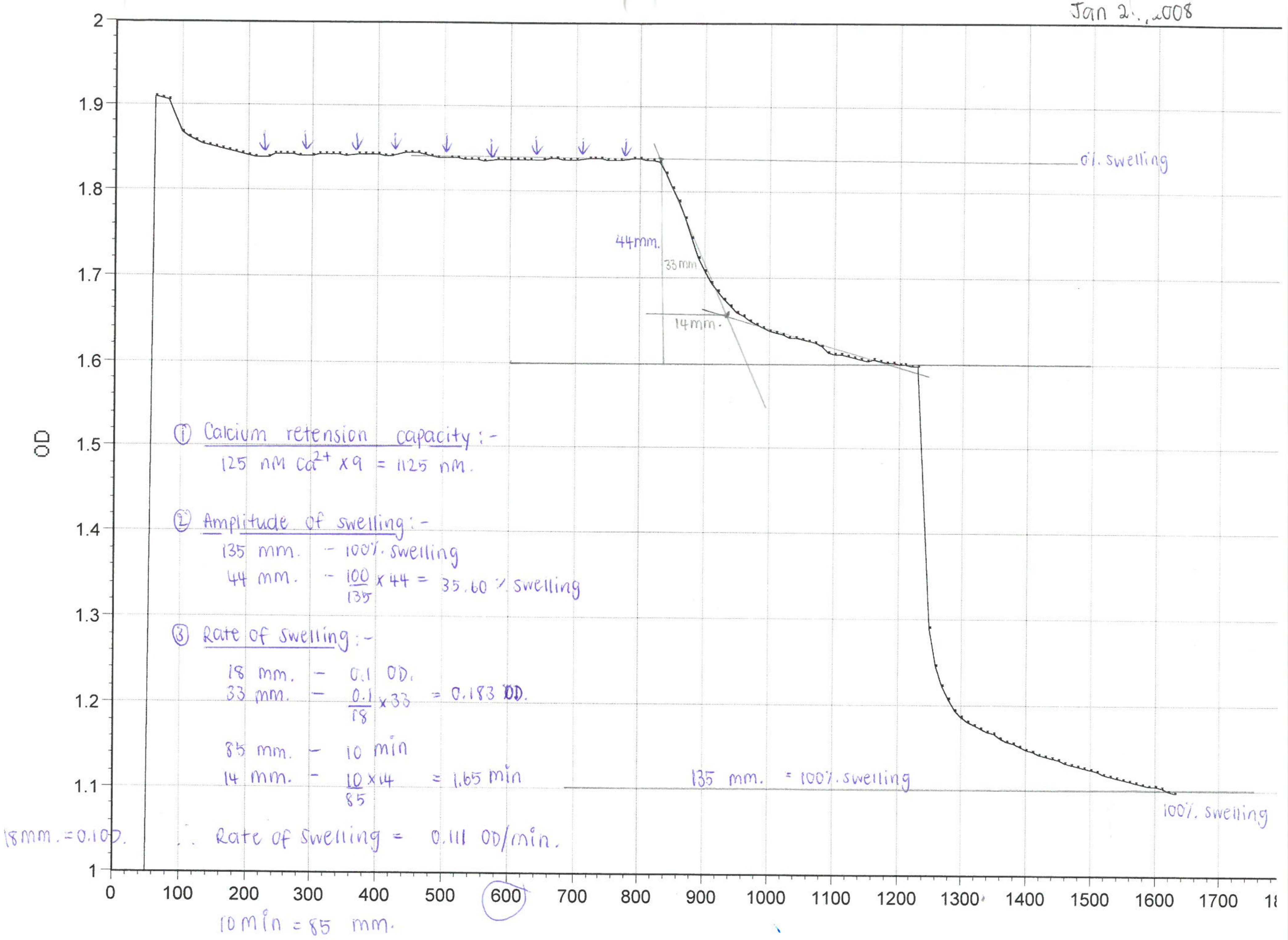

SSM

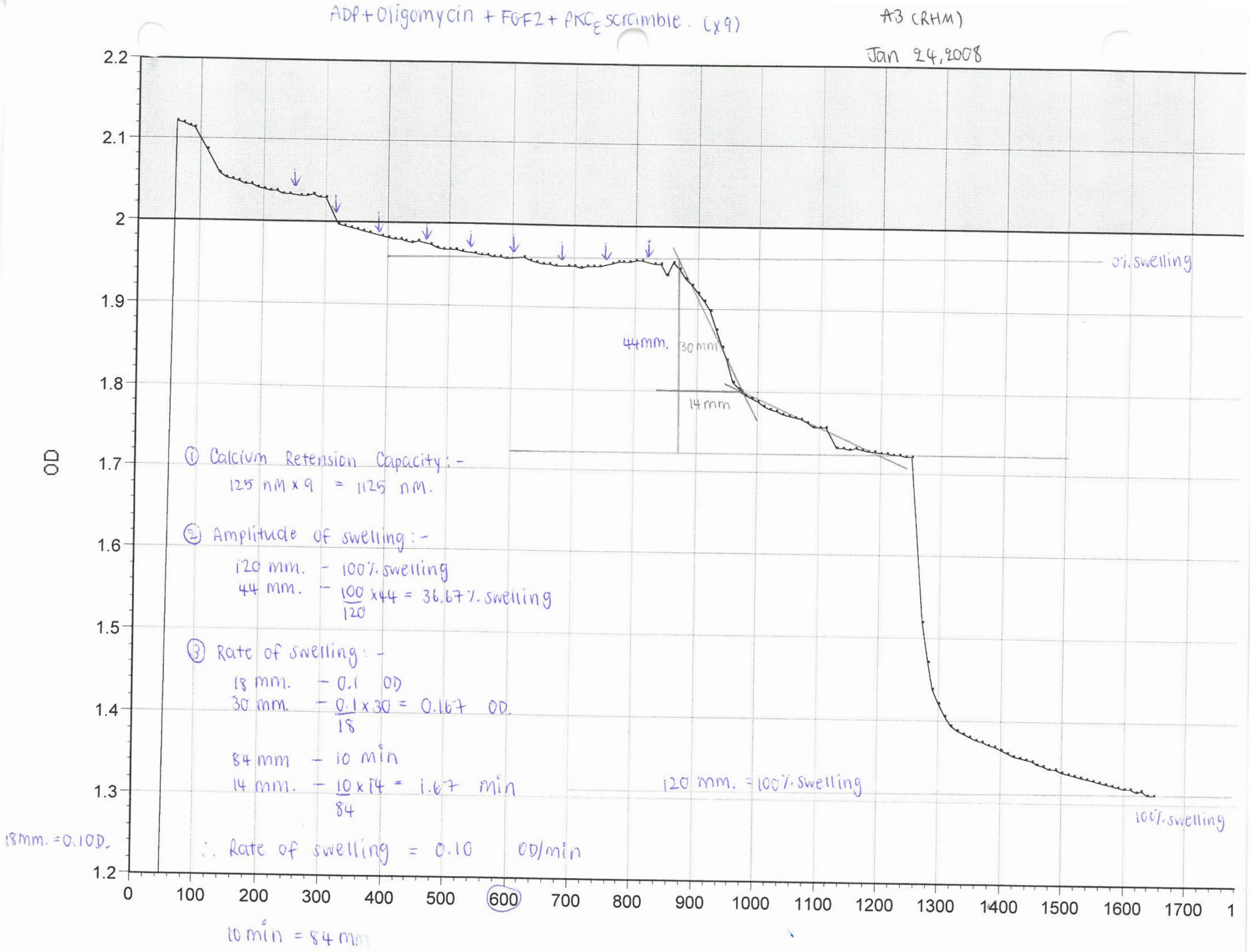
ADP + Oligomycin + FGF2 + PKC$_\varepsilon$ scramble. (x9)
A3 (RHM)
Jan 24, 2008
OD
2.2
2.1
2
1.9
1.8
1.7
1.6
1.5
1.4
1.3
1.2
0
100
200
300
400
500
600
700
800
900
1000
1100
1200
1300
1400
1500
1600
1700
1
0% swelling
44 mm.
30 mm
14 mm
① Calcium Retension Capacity :-
125 nM x 9 = 1125 nM.
② Amplitude of swelling :-
120 mm. - 100% swelling
44 mm. - 100/120 x 44 = 36.67% swelling
③ Rate of swelling :-
18 mm. - 0.1 OD
30 mm. - 0.1/18 x 30 = 0.167 OD.
84 mm - 10 min
14 mm. - 10/84 x 14 = 1.67 min
120 mm. = 100% swelling
100% swelling
18 mm. = 0.1 OD.
∴ Rate of swelling = 0.10 OD/min
10 min = 84 mm

LIVER

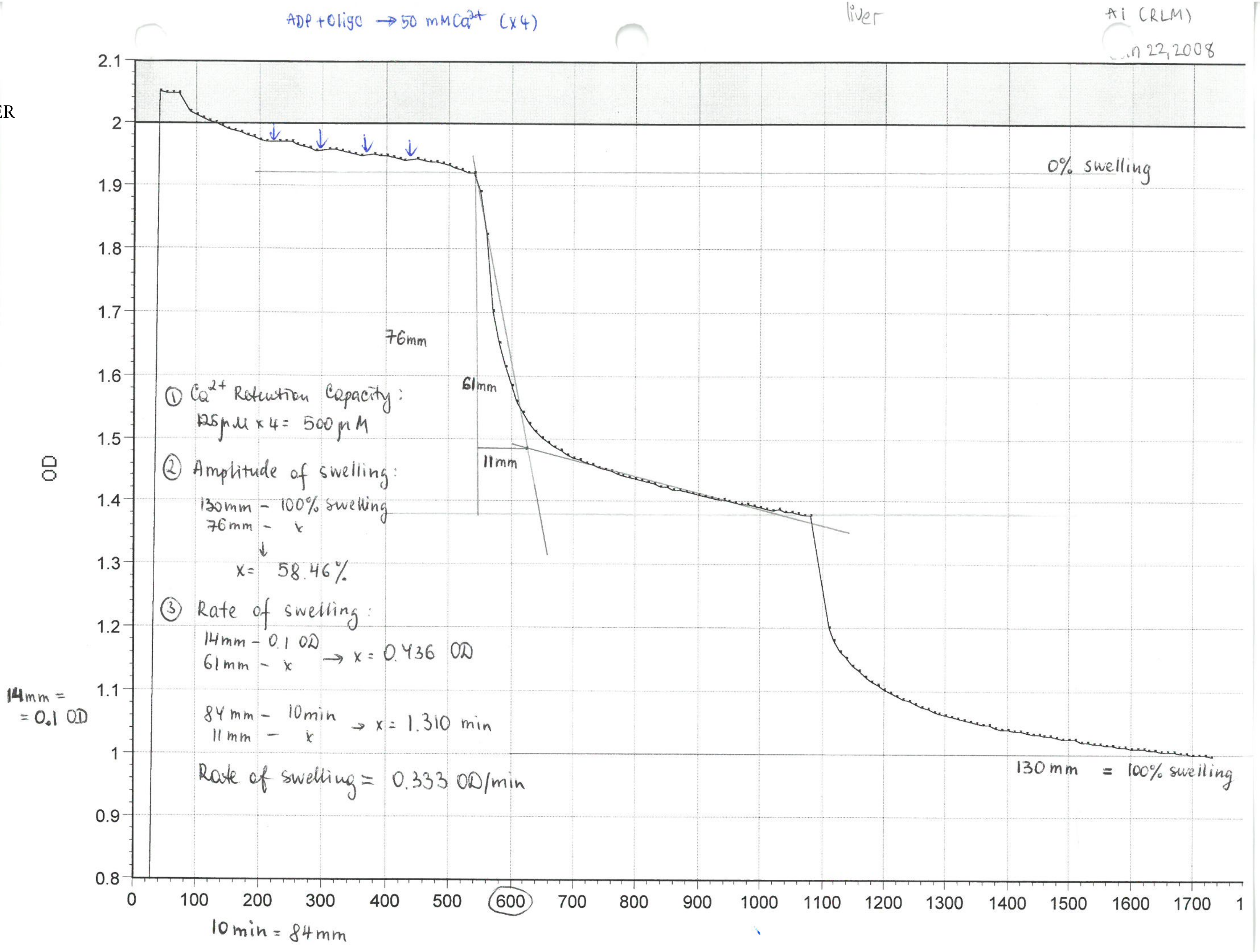

ADP + Oligo → 50 mM Ca2+ (x4)
liver
Ai (RLM)
…n 22, 2008
0% swelling
76mm
61mm
11mm
OD
① Ca2+ Retention Capacity:
125 µM x 4 = 500 µM
② Amplitude of swelling:
130mm - 100% swelling
76mm - x
x = 58.46%
③ Rate of swelling:
14mm - 0.1 OD
61mm - x
→ x = 0.436 OD
84 mm - 10 min
11 mm - x
→ x = 1.310 min
Rate of swelling = 0.333 OD/min
130 mm = 100% swelling
14mm = 0.1 OD
10 min = 84 mm
2.1
2
1.9
1.8
1.7
1.6
1.5
1.4
1.3
1.2
1.1
1
0.9
0.8
0
100
200
300
400
500
600
700
800
900
1000
1100
1200
1300
1400
1500
1600
1700

LIVER

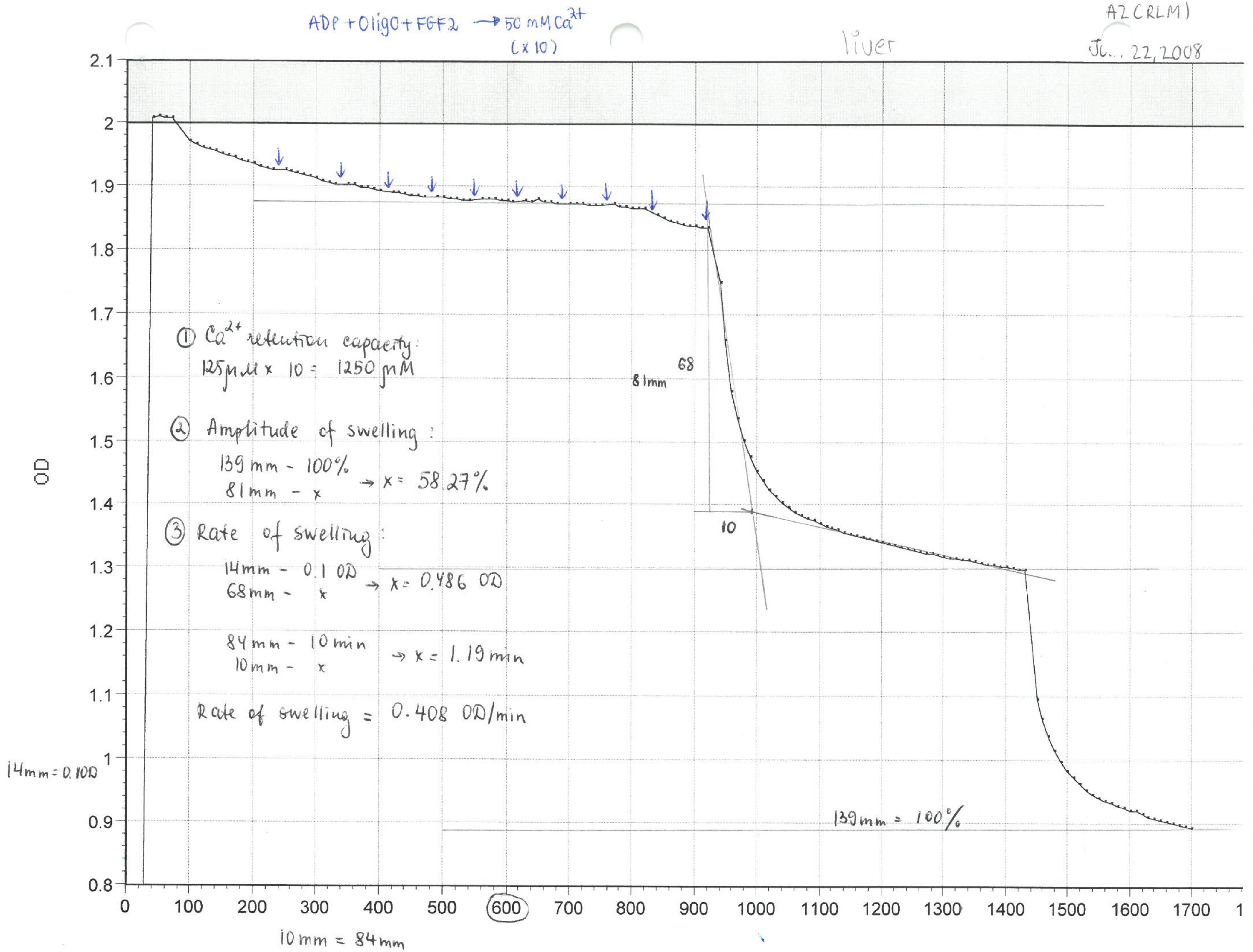

ADP + Oligo + FGF2 → 50 mM Ca^{2+} (x 10)
liver
A2 (RLM)
Ju... 22, 2008
OD
① Ca^{2+} retention capacity:
125 µM x 10 = 1250 µM
② Amplitude of swelling:
139 mm - 100%
81 mm - x
→ x = 58.27%
③ Rate of swelling:
14 mm - 0.1 OD
68 mm - x
→ x = 0.486 OD
84 mm - 10 min
10 mm - x
→ x = 1.19 min
Rate of swelling = 0.408 OD/min
68
81 mm
10
14 mm = 0.1 OD
139 mm = 100%
10 mm = 84 mm

LIVER

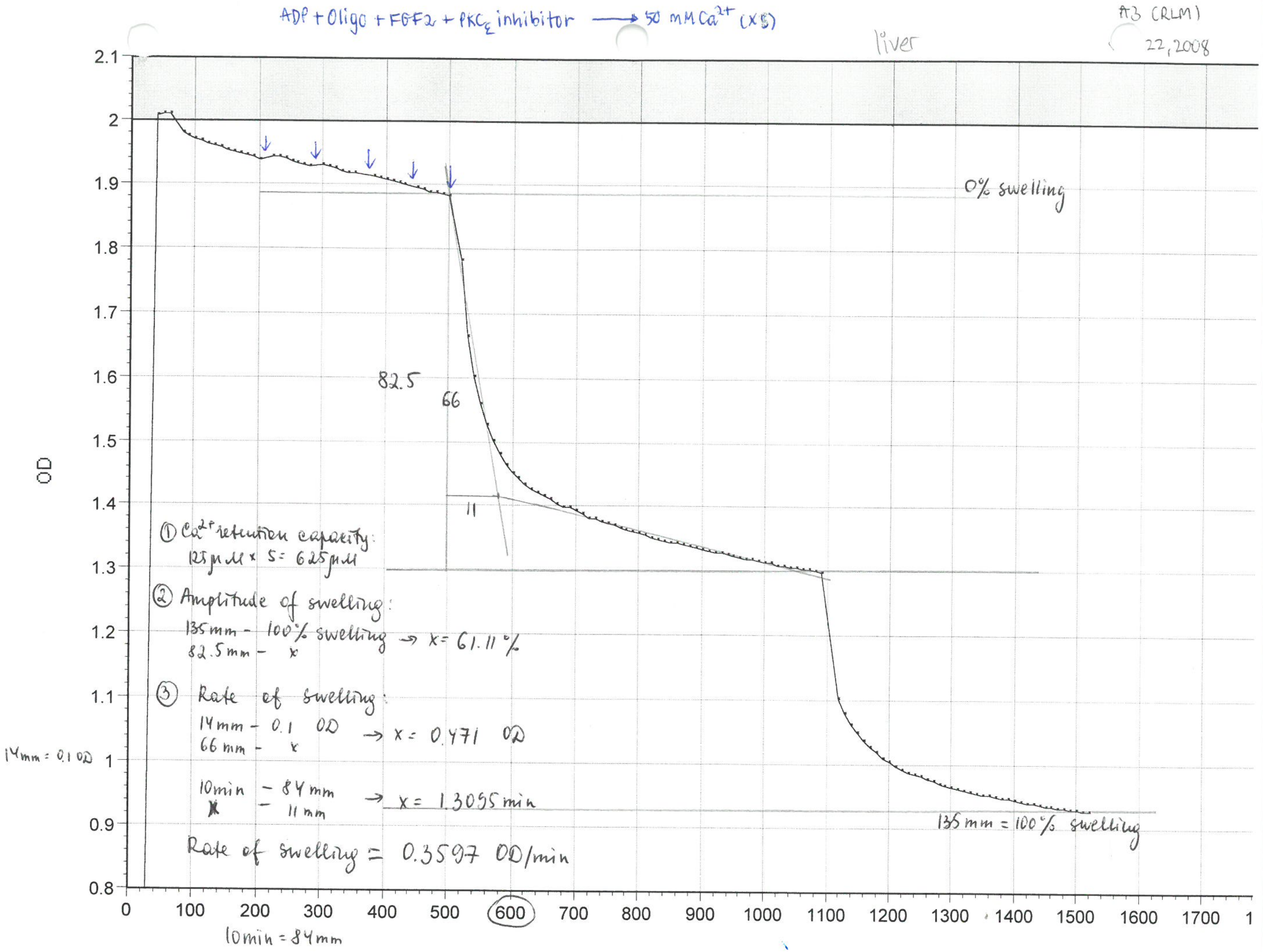
ADP + Oligo + FGF2 + PKCε inhibitor → 50 mM Ca2+ (x5)
liver
A3 (RLM)
22, 2008
OD
0% swelling
82.5
66
11
① Ca2+ retention capacity:
125 μM x 5 = 625 μM
② Amplitude of swelling:
135 mm - 100% swelling → x = 61.11 %
82.5 mm - x
③ Rate of swelling:
14 mm - 0.1 OD → x = 0.471 OD
66 mm - x
10 min - 84 mm → x = 1.3095 min
x - 11 mm
Rate of swelling = 0.3597 OD/min
14mm = 0.1 OD
135 mm = 100% swelling
10min = 84mm
2.1
2
1.9
1.8
1.7
1.6
1.5
1.4
1.3
1.2
1.1
1
0.9
0.8
0
100
200
300
400
500
600
700
800
900
1000
1100
1200
1300
1400
1500
1600
1700

LIVER

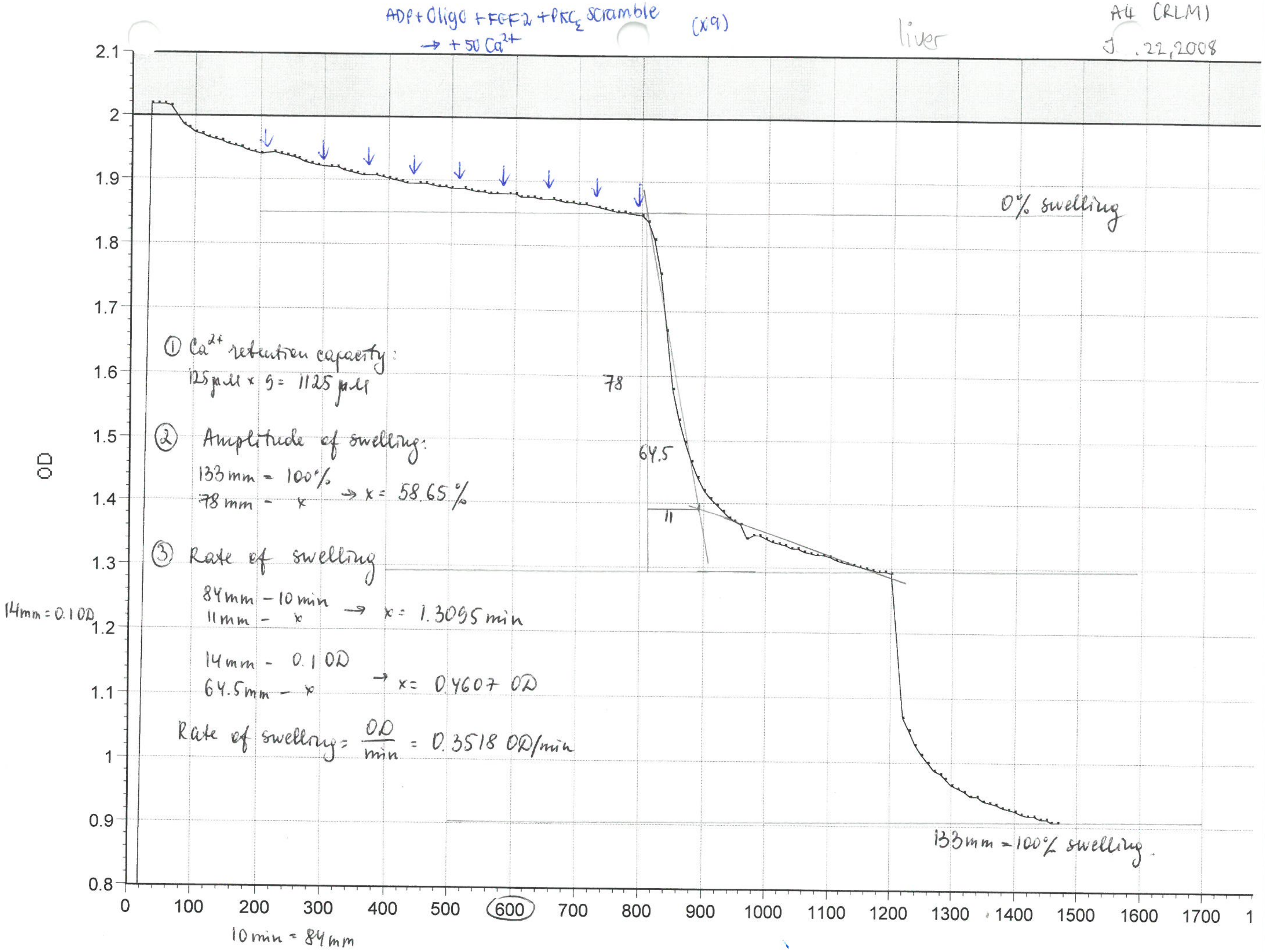
ADP+Oligo +FGF2 +PKCε scramble
→ + 50 Ca2+
(x9)
liver
A4 (RLM)
J .22,2008
OD
0% swelling
78
64.5
11
① Ca2+ retention capacity:
125 μM × 9 = 1125 μM
② Amplitude of swelling:
133 mm = 100%
78 mm - x → x = 58.65 %
③ Rate of swelling
84 mm - 10 min
11 mm - x → x = 1.3095 min
14 mm - 0.1 OD
64.5 mm - x → x = 0.4607 OD
Rate of swelling = OD/min = 0.3518 OD/min
14mm = 0.1 OD
133 mm = 100% swelling.
10 min = 84 mm
2.1
2
1.9
1.8
1.7
1.6
1.5
1.4
1.3
1.2
1.1
1
0.9
0.8
0
100
200
300
400
500
600
700
800
900
1000
1100
1200
1300
1400
1500
1600
1700
1

LIVER

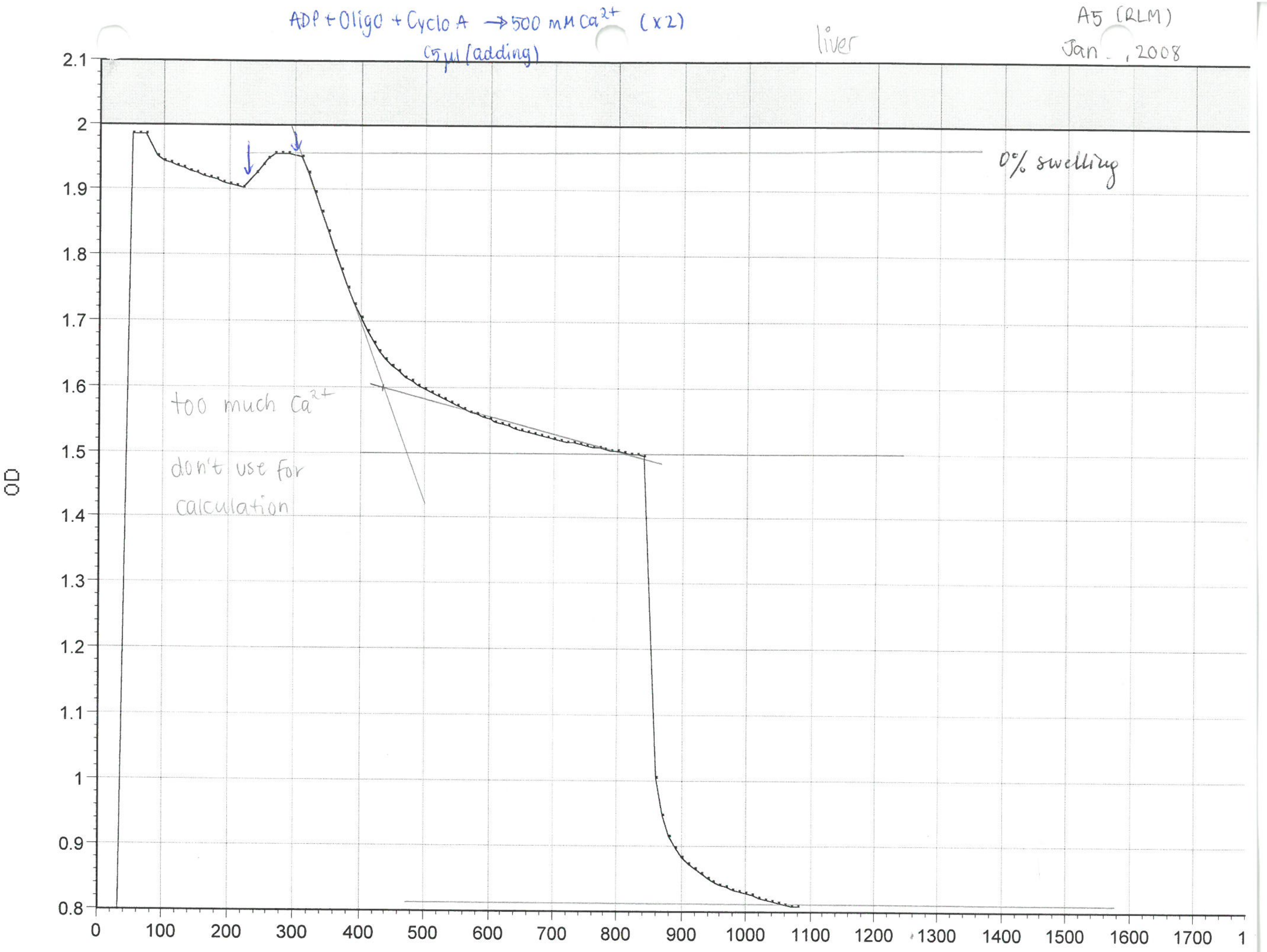
ADP + Oligo + Cyclo A → 500 mM Ca^{2+} (x2)
(5μl/adding)
liver
A5 (RLM)
Jan , 2008
0% swelling
too much Ca^{2+}
don't use for
calculation
OD
2.1
2
1.9
1.8
1.7
1.6
1.5
1.4
1.3
1.2
1.1
1
0.9
0.8
0
100
200
300
400
500
600
700
800
900
1000
1100
1200
1300
1400
1500
1600
1700

LIVER

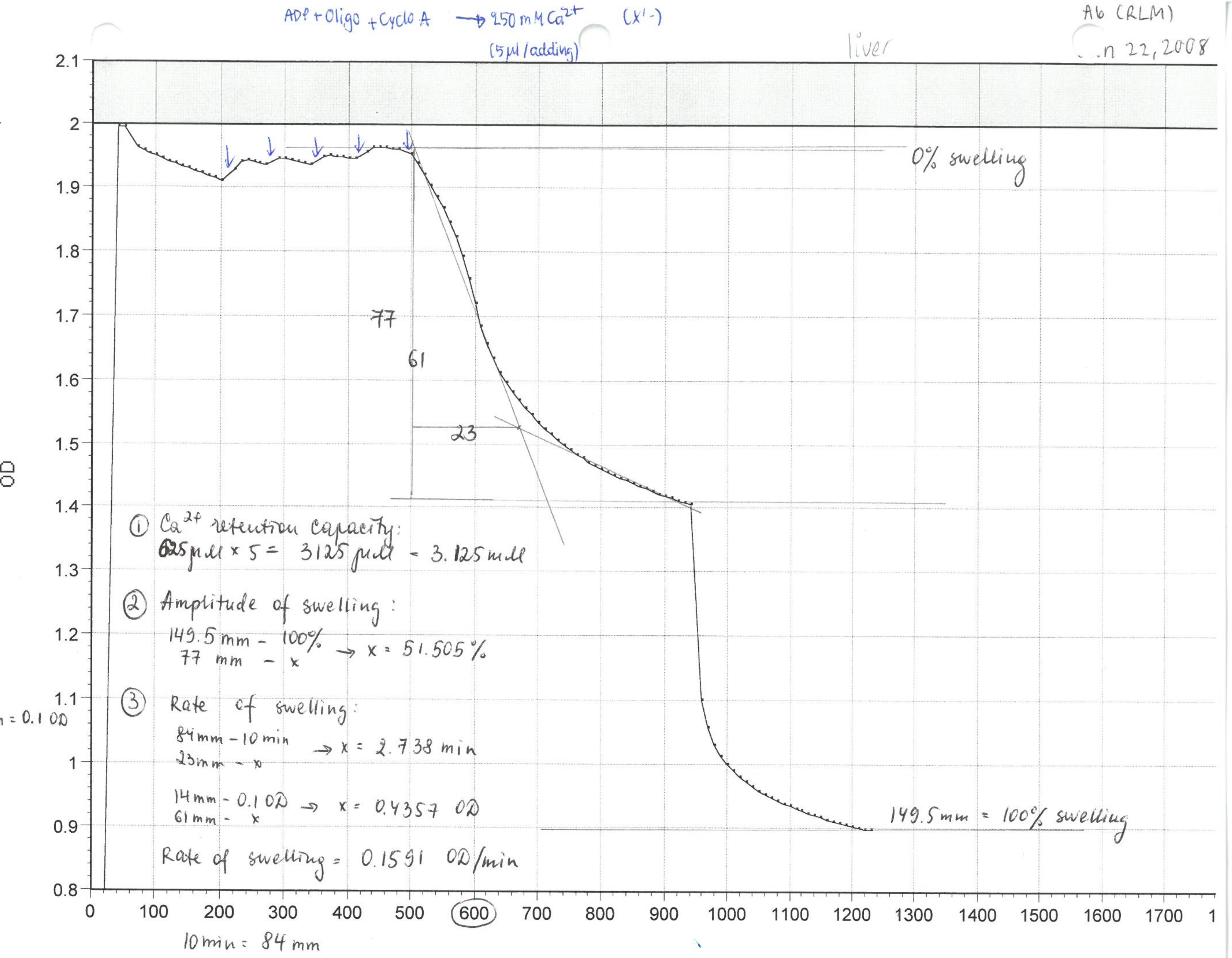

ADP + Oligo + Cyclo A → 250 mM Ca^{2+} (x'-)
(5µl / adding)
liver
A6 (RLM)
. .n 22, 2008
OD
2.1
2
1.9
1.8
1.7
1.6
1.5
1.4
1.3
1.2
1.1
1
0.9
0.8
0
100
200
300
400
500
600
700
800
900
1000
1100
1200
1300
1400
1500
1600
1700
1
0% swelling
77
61
23
149.5 mm = 100% swelling
14mm = 0.1 OD
10 min = 84 mm
① Ca^{2+} retention capacity:
625 µM × 5 = 3125 µM = 3.125 mM
② Amplitude of swelling:
149.5 mm – 100%
77 mm – x
→ x = 51.505 %
③ Rate of swelling:
84 mm – 10 min
23 mm – x
→ x = 2.738 min
14 mm – 0.1 OD
61 mm – x
→ x = 0.4357 OD
Rate of swelling = 0.1591 OD/min

LIVER

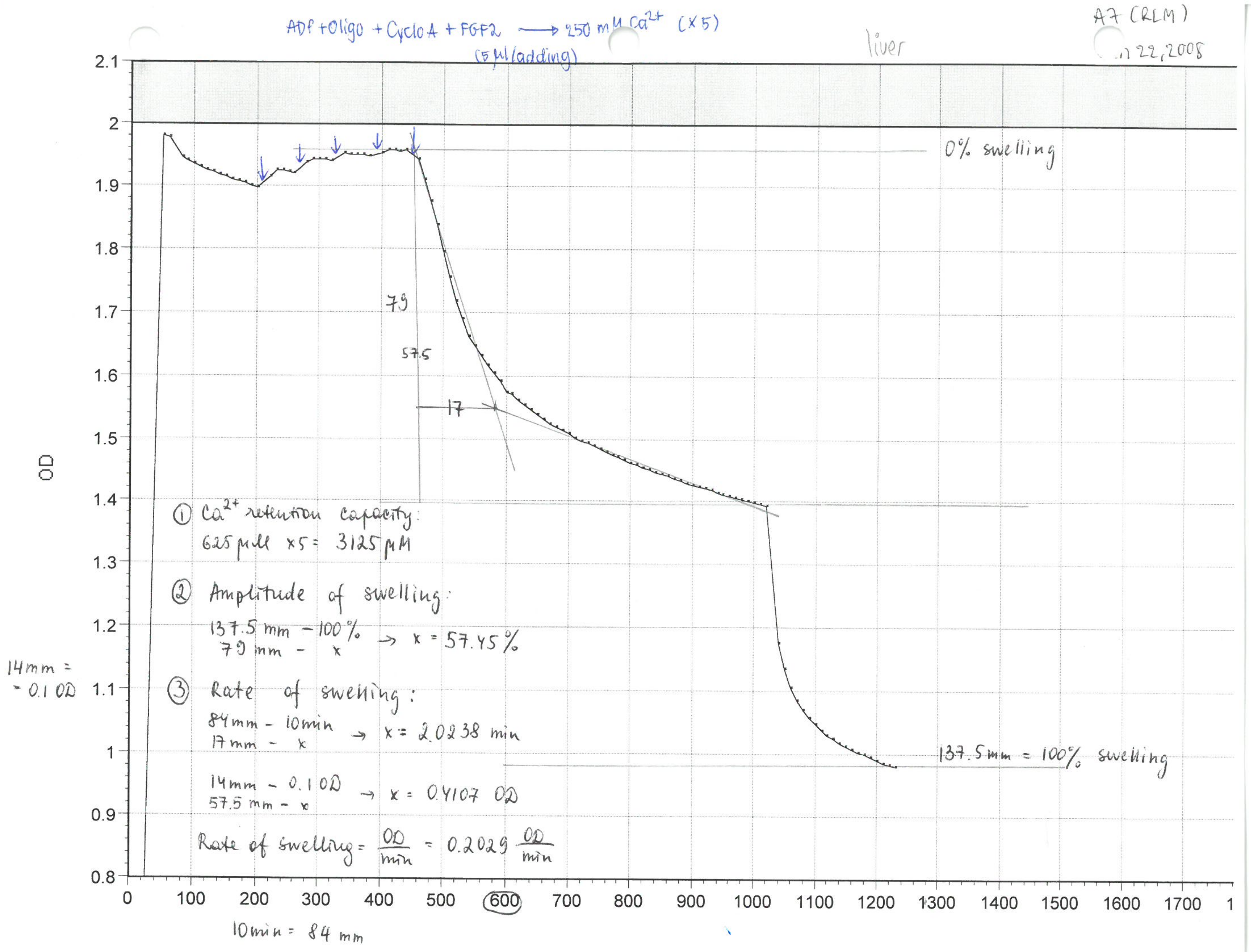
ADP + Oligo + Cyclo A + FGF2 ⟶ 250 mM Ca^{2+} (x5)
(5 μl/adding)
liver
A7 (RLM)
.. 22, 2008
OD
2.1
2
1.9
1.8
1.7
1.6
1.5
1.4
1.3
1.2
1.1
1
0.9
0.8
0
100
200
300
400
500
600
700
800
900
1000
1100
1200
1300
1400
1500
1600
1700
0% swelling
79
57.5
17
137.5 mm = 100% swelling
14 mm = = 0.1 OD
10 min = 84 mm
① Ca^{2+} retention capacity:
625 μM x5 = 3125 μM
② Amplitude of swelling:
137.5 mm − 100%
79 mm − x → x = 57.45%
③ Rate of swelling:
84 mm − 10 min
17 mm − x → x = 2.0238 min
14 mm − 0.1 OD
57.5 mm − x → x = 0.4107 OD
Rate of swelling = OD/min = 0.2029 OD/min

LIVER

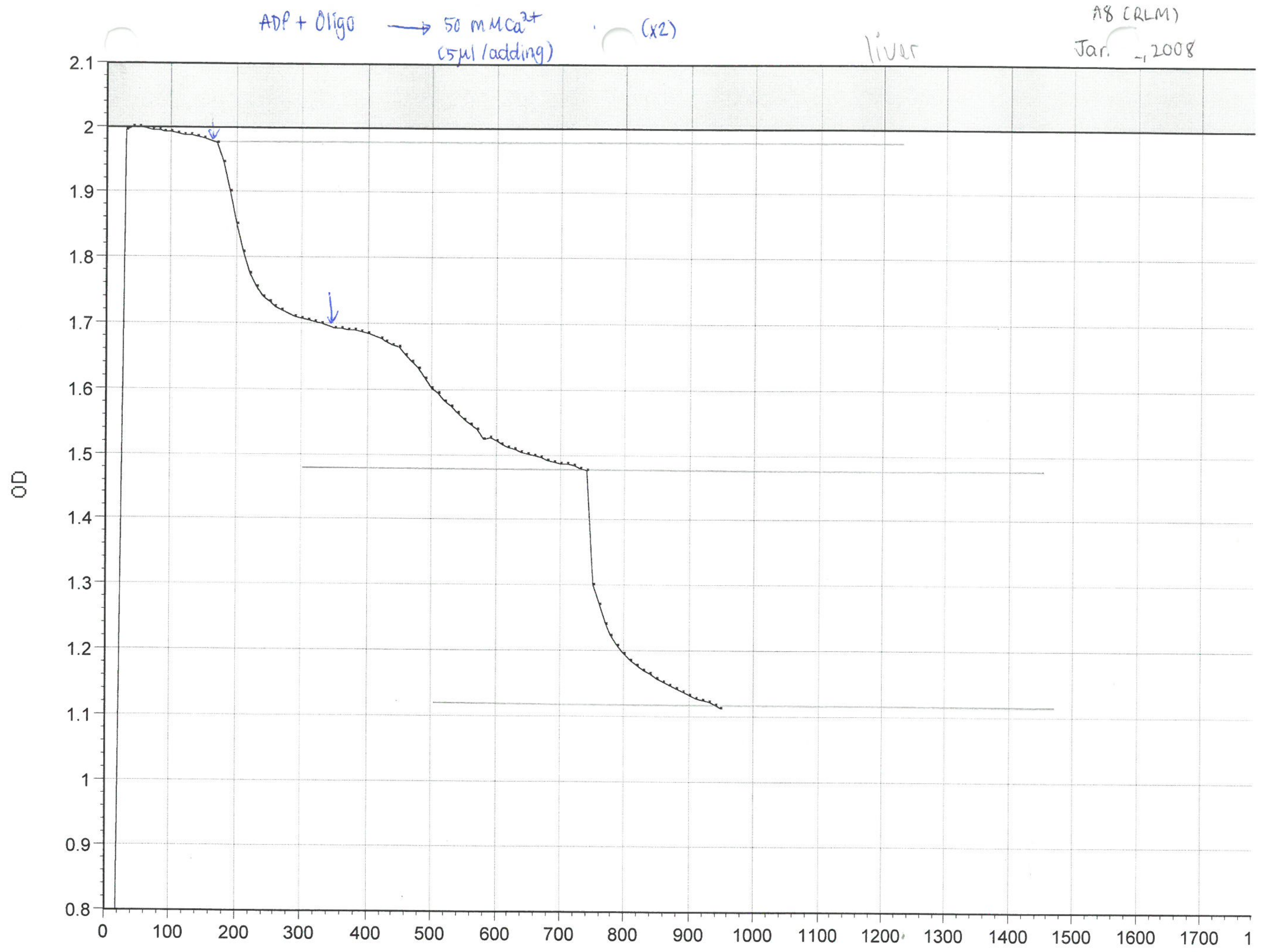
ADP + Oligo → 50 mMCa2+
(5μl /adding)
(x2)
liver
A8 (RLM)
Jan. , 2008
OD
2.1
2
1.9
1.8
1.7
1.6
1.5
1.4
1.3
1.2
1.1
1
0.9
0.8
0
100
200
300
400
500
600
700
800
900
1000
1100
1200
1300
1400
1500
1600
1700

LIVER

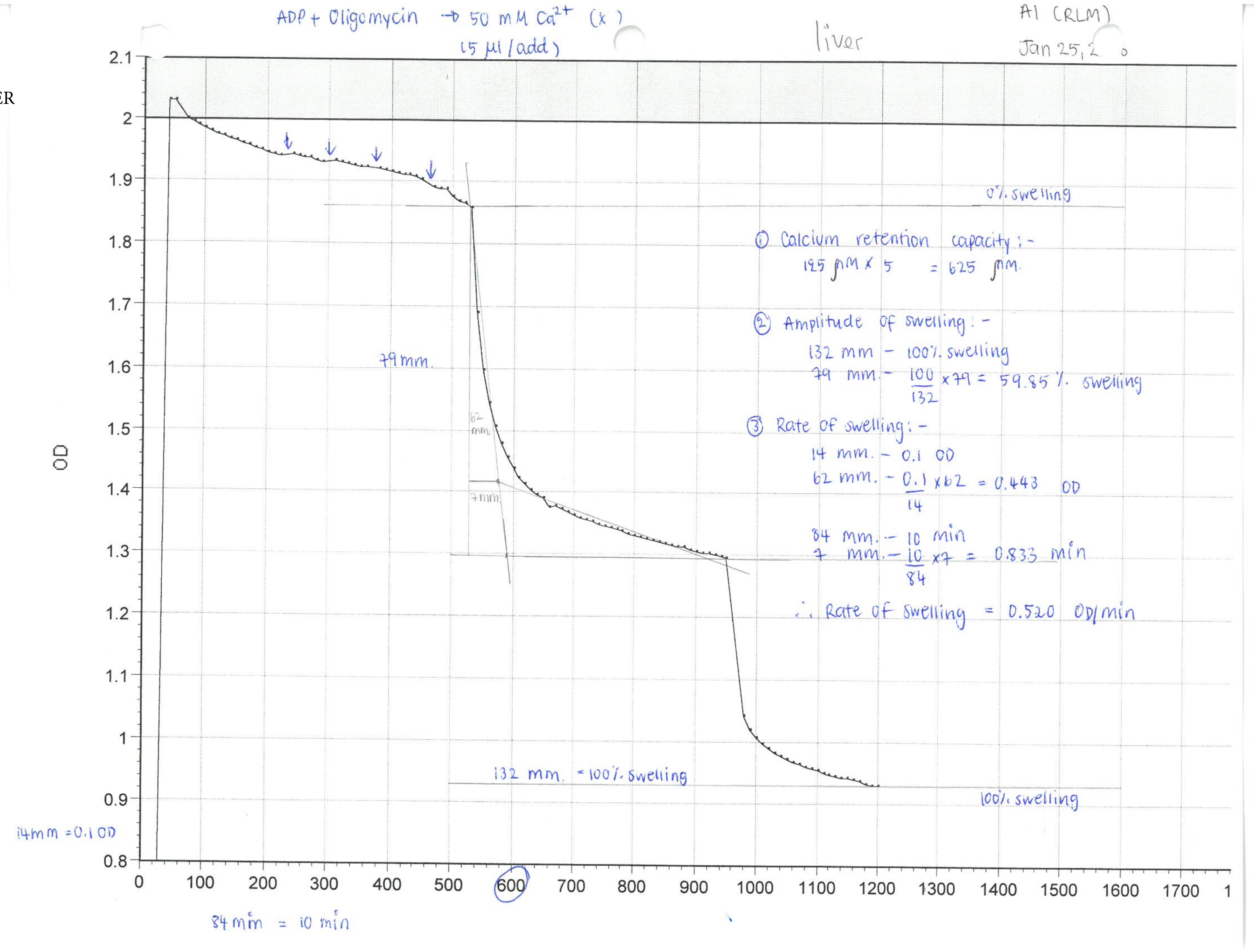
ADP + Oligomycin → 50 mM Ca^{2+} (x) (5 µl / add)
liver
A1 (RLM)
Jan 25, 2
OD
0% swelling
① Calcium retention capacity :-
125 µM x 5 = 625 µM.
② Amplitude of swelling :-
132 mm – 100% swelling
79 mm.– 100/132 x 79 = 59.85 % swelling
③ Rate of swelling :-
14 mm. – 0.1 OD
62 mm. – 0.1/14 x 62 = 0.443 OD
84 mm. – 10 min
7 mm. – 10/84 x 7 = 0.833 min
∴ Rate of swelling = 0.520 OD/min
79 mm.
62 mm.
7 mm.
132 mm. = 100% swelling
100% swelling
14mm = 0.1 OD
84 mm = 10 min
2.1
2
1.9
1.8
1.7
1.6
1.5
1.4
1.3
1.2
1.1
1
0.9
0.8
0
100
200
300
400
500
600
700
800
900
1000
1100
1200
1300
1400
1500
1600
1700
1

LIVER

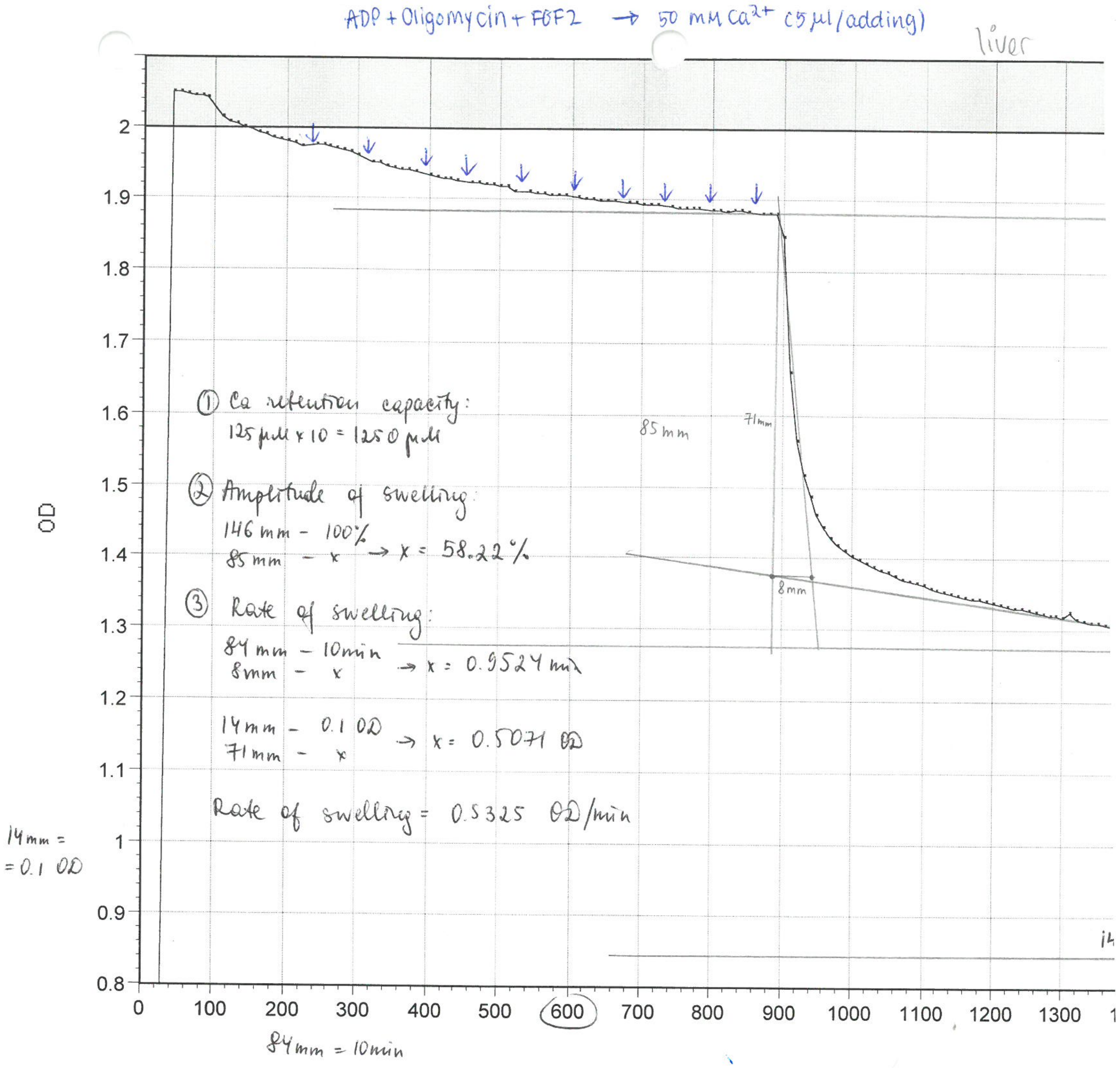
ADP + Oligomycin + FOF2 → 50 mM Ca2+ (5 μl/adding)
liver
OD
2
1.9
1.8
1.7
1.6
1.5
1.4
1.3
1.2
1.1
1
0.9
0.8
0
100
200
300
400
500
600
700
800
900
1000
1100
1200
1300
① Ca retention capacity:
125 μM x 10 = 1250 μM
② Amplitude of swelling:
146 mm - 100%
85 mm - x → x = 58.22%
③ Rate of swelling:
84 mm - 10 min
8 mm - x → x = 0.9524 min
14 mm - 0.1 OD
71 mm - x → x = 0.5071 OD
Rate of swelling = 0.5325 OD/min
85 mm
71 mm
8 mm
14 mm =
= 0.1 OD
84 mm = 10 min

LIVER

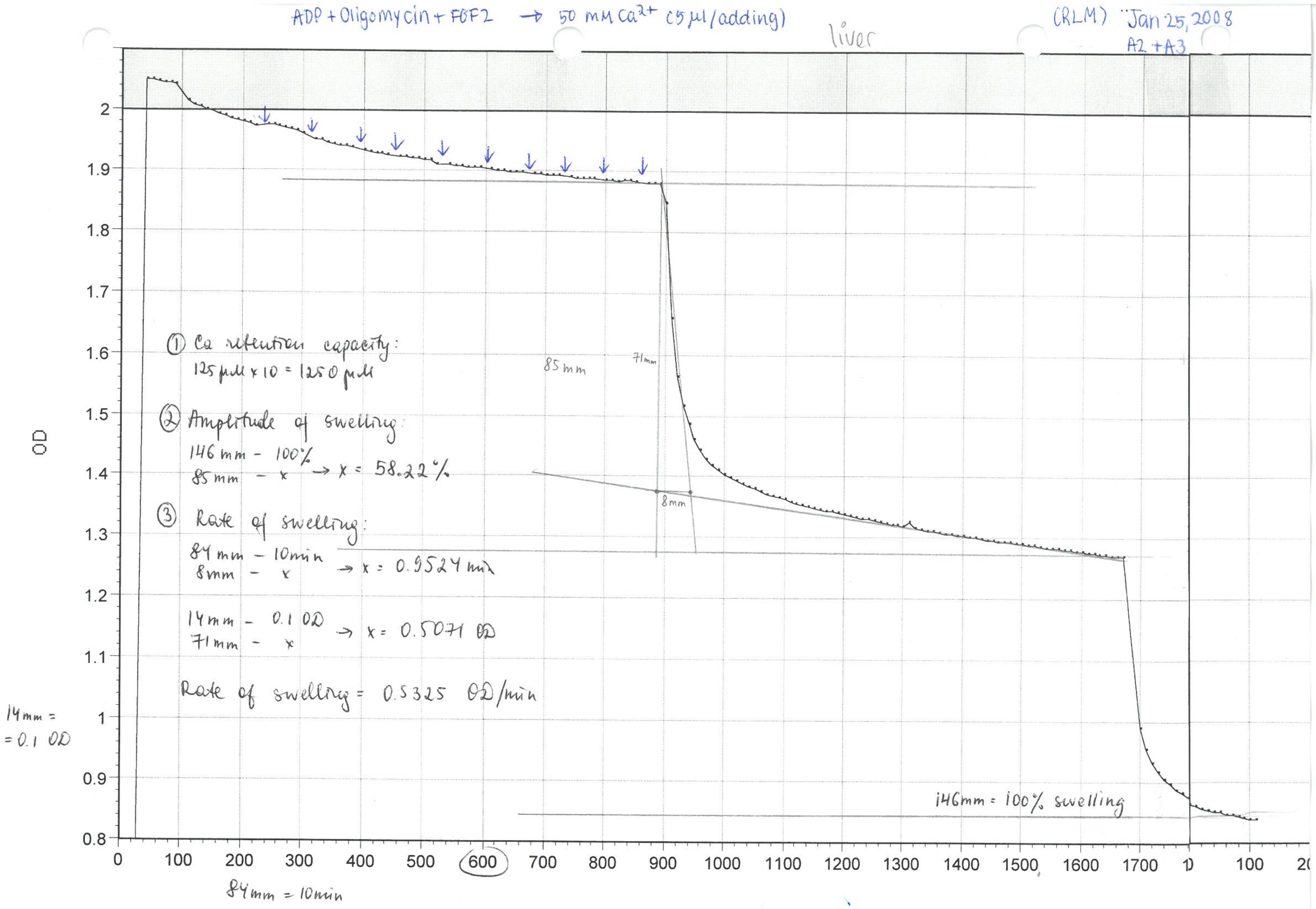

ADP + Oligomycin + FOF2 → 50 mM Ca^{2+} (5 µl/adding)
(RLM) Jan 25, 2008
A2 + A3
liver
OD
① Ca retention capacity:
125 µM x 10 = 1250 µM
② Amplitude of swelling:
146 mm - 100%
85 mm - x → x = 58.22%
③ Rate of swelling:
84 mm - 10 min
8 mm - x → x = 0.9524 min
14 mm - 0.1 OD
71 mm - x → x = 0.5071 OD
Rate of swelling = 0.5325 OD/min
85 mm
71 mm
8 mm
14 mm = = 0.1 OD
146 mm = 100% swelling
84 mm = 10 min

LIVER

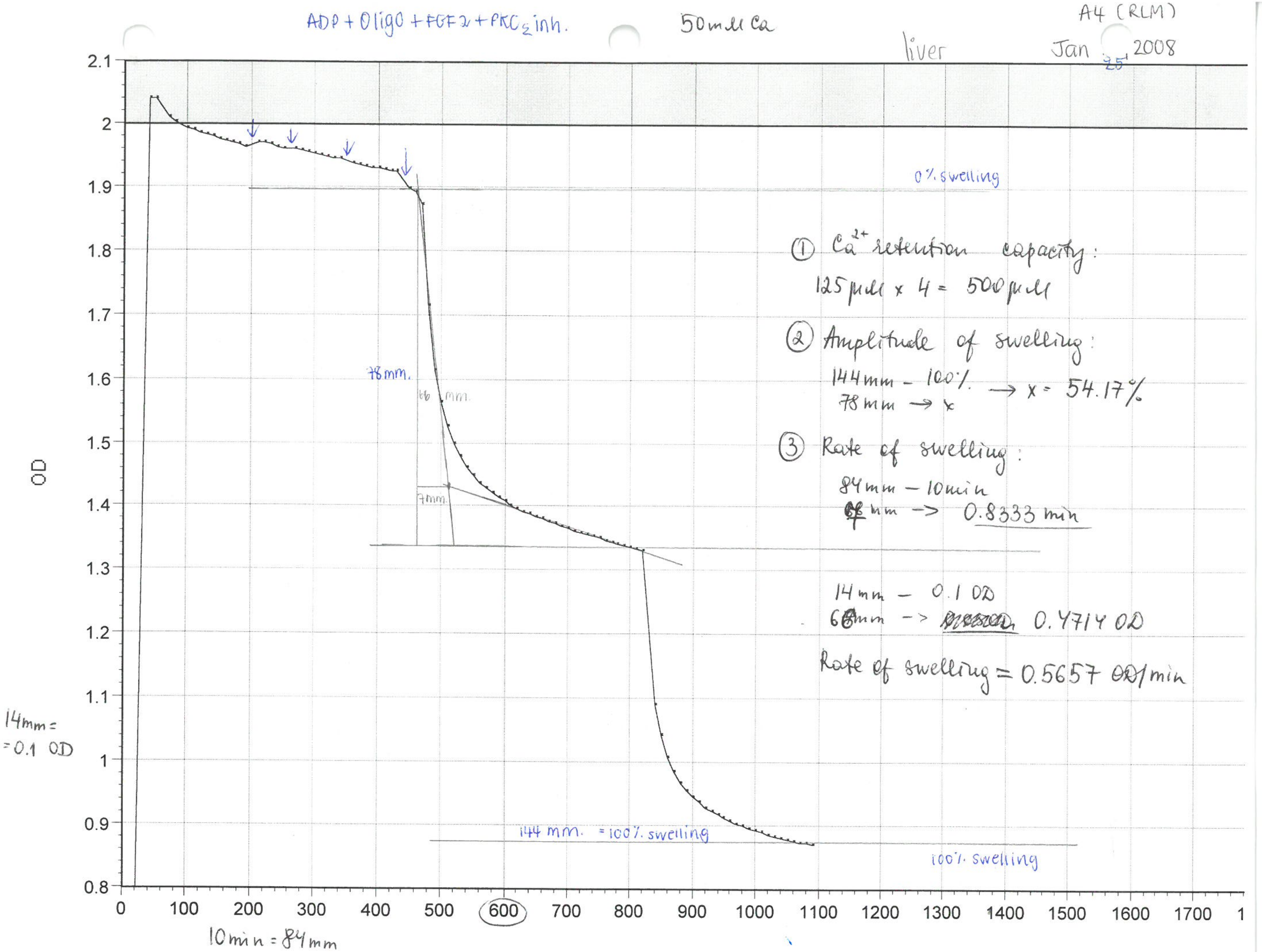

ADP + Oligo + FGF 2 + PKCε inh.
50 mM Ca
A4 (RLM)
liver
Jan 25 2008
OD
10 min = 84 mm
14mm = = 0.1 OD
0% swelling
100% swelling
144 mm. = 100% swelling
78mm.
66 mm.
7mm.
① Ca²⁺ retention capacity:
125 µM x 4 = 500 µM
② Amplitude of swelling:
144mm – 100%
78 mm → x → x = 54.17%
③ Rate of swelling:
84mm – 10min
7 mm → 0.8333 min
14 mm – 0.1 OD
66mm → 0.4714 OD
Rate of swelling = 0.5657 OD/min

LIVER

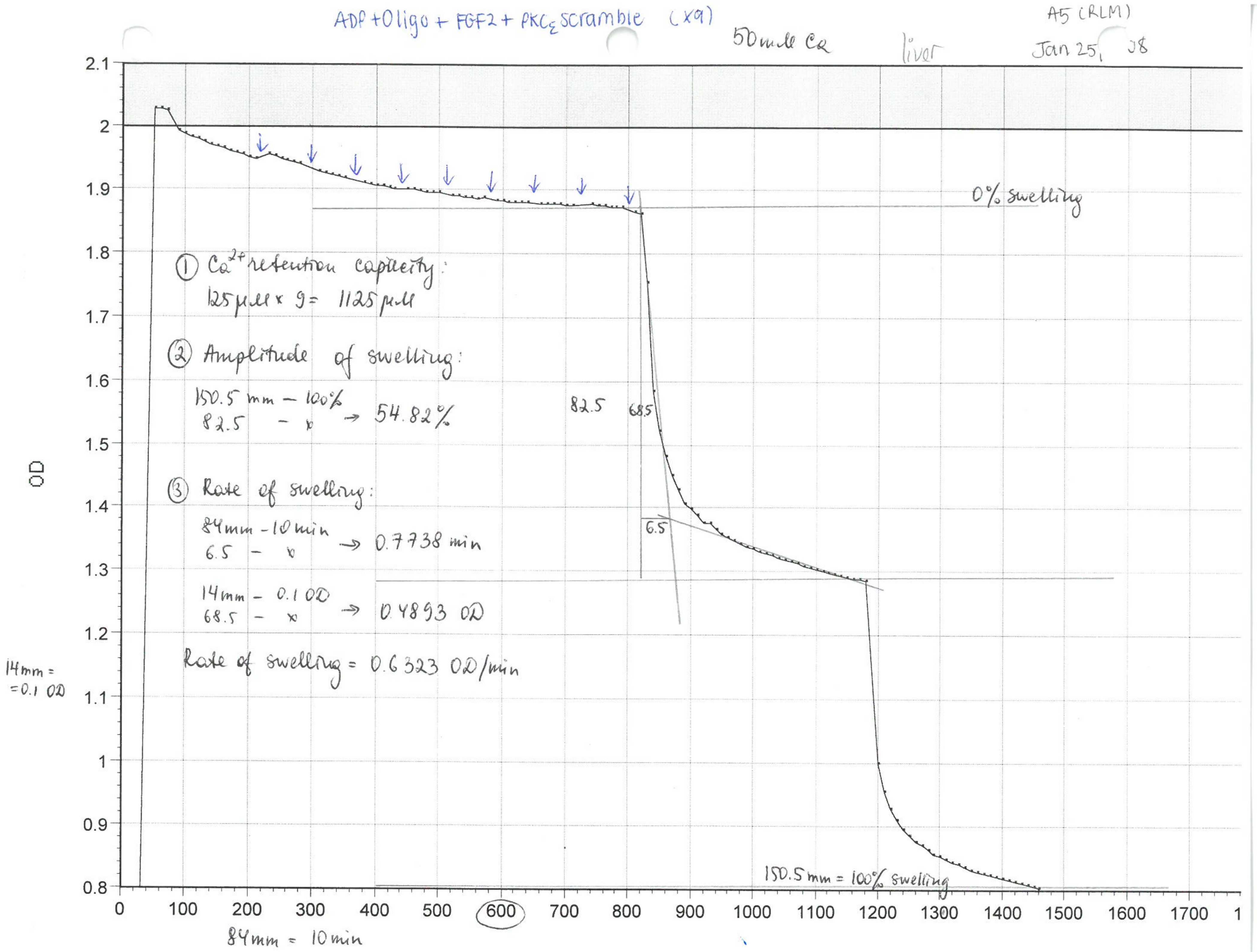
ADP + Oligo + FGF2 + PKCε scramble (x9)
50 µM Ca
liver
A5 (RLM)
Jan 25, 08
0% swelling
OD
① Ca^{2+} retention capacity:
125 µM x 9 = 1125 µM
② Amplitude of swelling:
150.5 mm — 100%
82.5 — x → 54.82%
③ Rate of swelling:
84 mm - 10 min
6.5 - x → 0.7738 min
14 mm - 0.1 OD
68.5 - x → 0.4893 OD
Rate of swelling = 0.6323 OD/min
82.5
68.5
6.5
14mm = 0.1 OD
150.5 mm = 100% swelling
84 mm = 10 min
2.1
2
1.9
1.8
1.7
1.6
1.5
1.4
1.3
1.2
1.1
1
0.9
0.8
0
100
200
300
400
500
600
700
800
900
1000
1100
1200
1300
1400
1500
1600
1700

LIVER

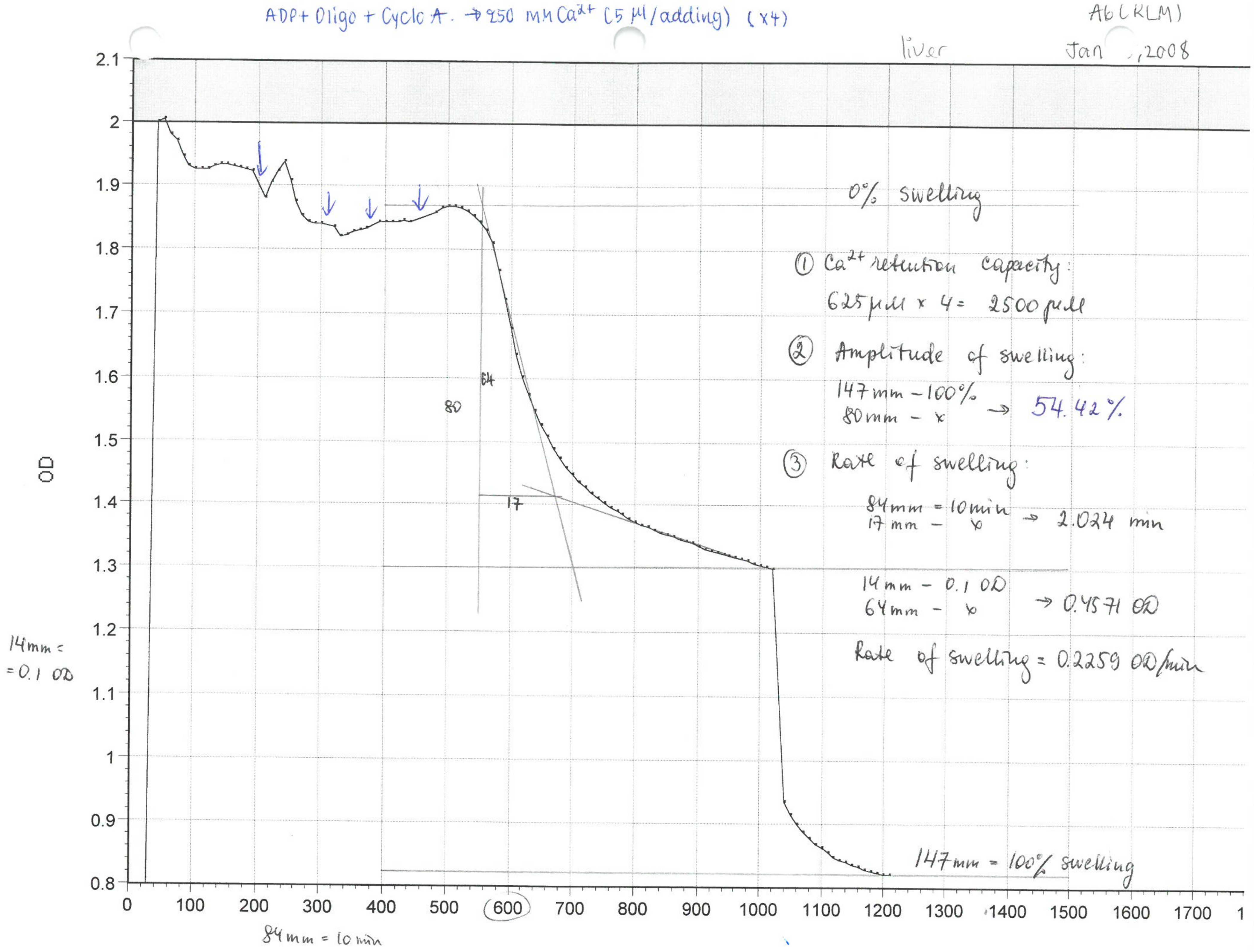
ADP+ Oligo + Cyclo A. → 250 mM Ca2+ (5 µl/adding) (x4)
A6 (RLM)
liver
Jan ,2008
OD
14mm = = 0.1 OD
0% swelling
① Ca2+ retention capacity:
625 µM x 4 = 2500 µM
② Amplitude of swelling:
147 mm – 100%
80 mm – x
→ 54.42%
③ Rate of swelling:
84 mm = 10 min
17 mm – x
→ 2.024 min
14 mm – 0.1 OD
64 mm – x
→ 0.4571 OD
Rate of swelling = 0.2259 OD/min
147 mm = 100% swelling
64
80
17
84 mm = 10 min
2.1
2
1.9
1.8
1.7
1.6
1.5
1.4
1.3
1.2
1.1
1
0.9
0.8
0
100
200
300
400
500
600
700
800
900
1000
1100
1200
1300
1400
1500
1600
1700

LIVER

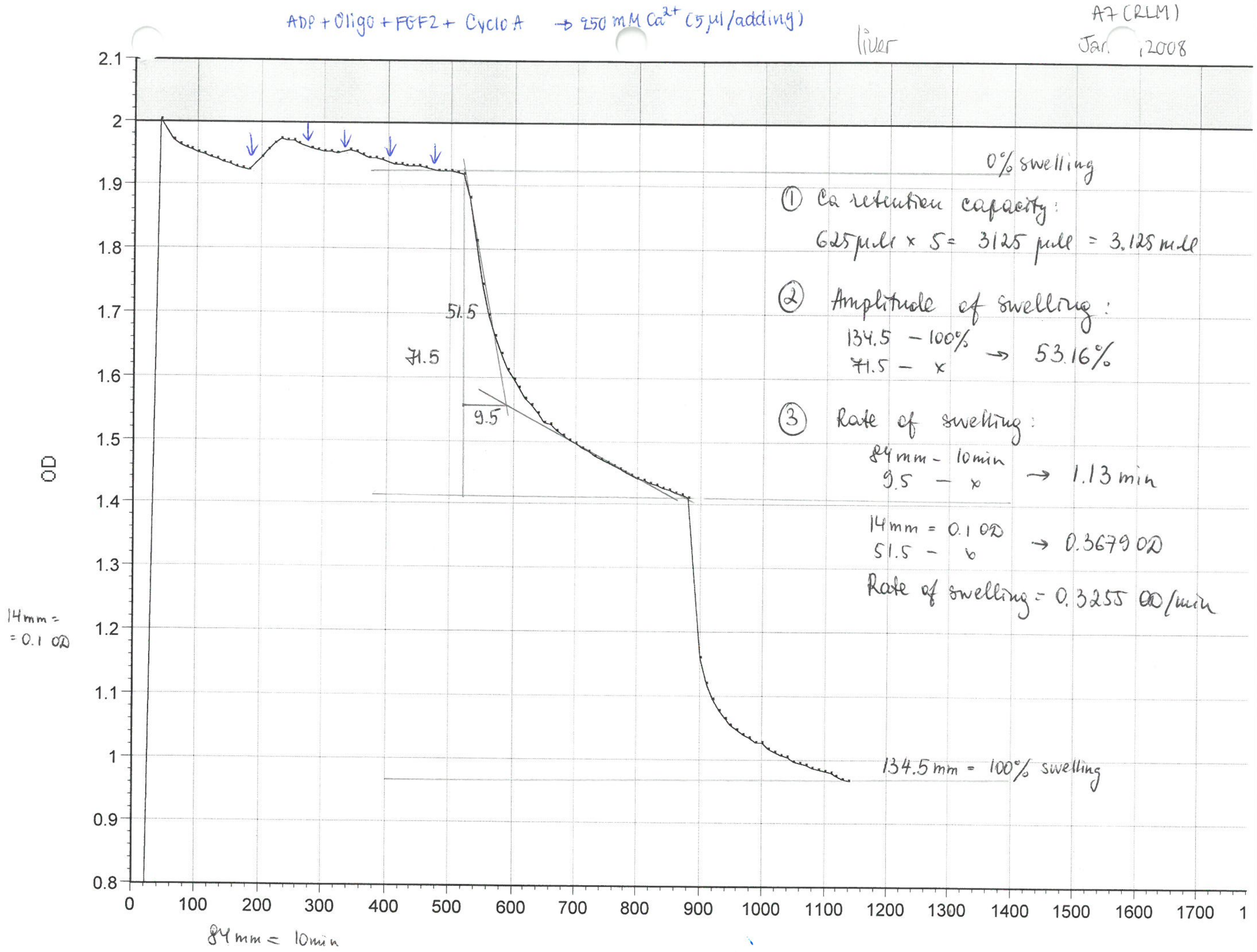
ADP + Oligo + FGF2 + Cyclo A → 250 mM Ca2+ (5 µl/adding)
A7 (RLM)
liver
Jan. , 2008
OD
14mm = = 0.1 OD
0% swelling
① Ca retention capacity:
625 µM × 5 = 3125 µM = 3.125 mM
② Amplitude of swelling:
134.5 – 100%
71.5 – x
→ 53.16%
③ Rate of swelling:
84 mm – 10 min
9.5 – x
→ 1.13 min
14 mm = 0.1 OD
51.5 – b
→ 0.3679 OD
Rate of swelling = 0.3255 OD/min
51.5
71.5
9.5
134.5 mm = 100% swelling
84 mm = 10 min
2.1
2
1.9
1.8
1.7
1.6
1.5
1.4
1.3
1.2
1.1
1
0.9
0.8
0
100
200
300
400
500
600
700
800
900
1000
1100
1200
1300
1400
1500
1600
1700

LIVER

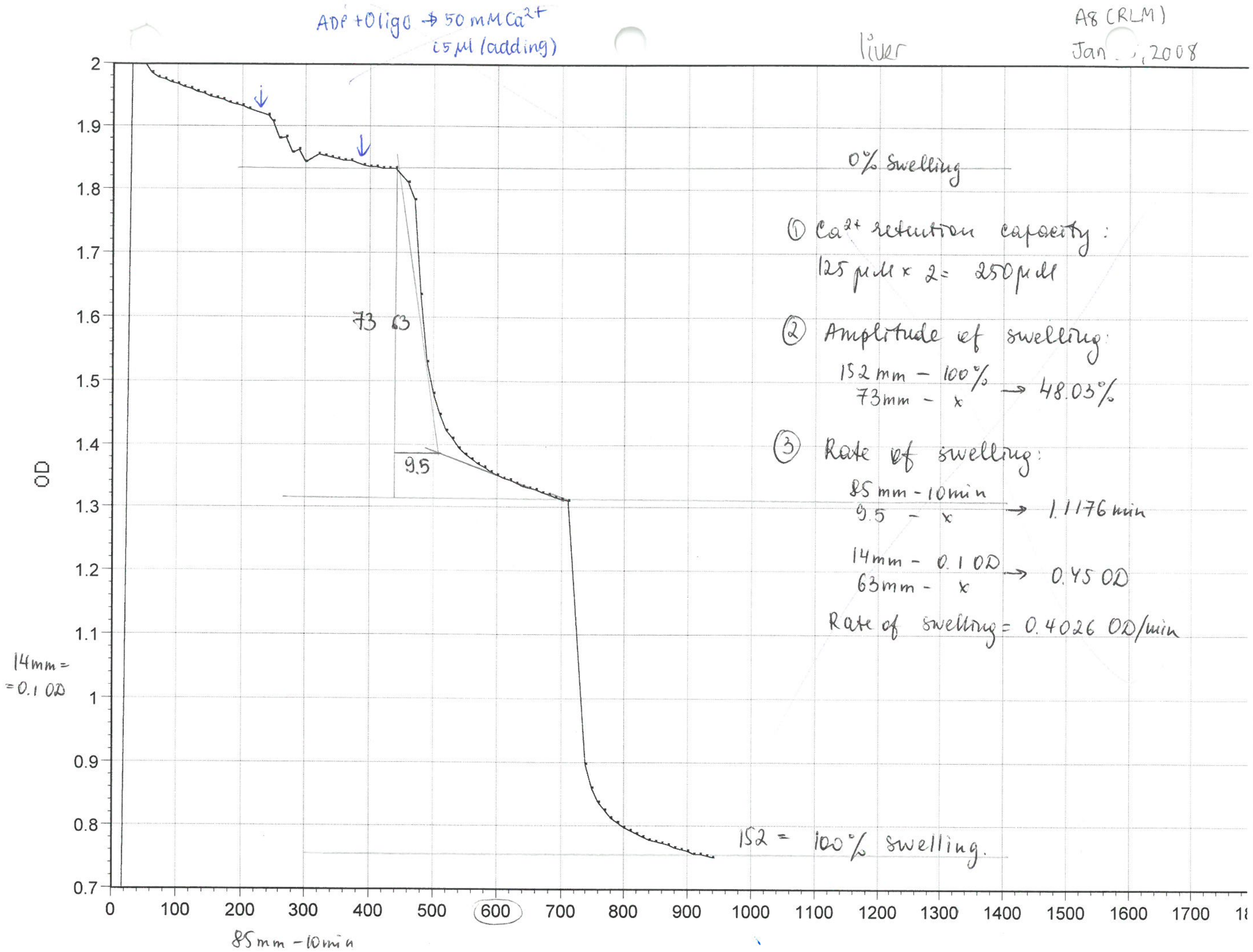
ADP + Oligo → 50 mM Ca^{2+}
5 μl (adding)
liver
A8 (RLM)
Jan. , 2008
OD
0% swelling
① Ca^{2+} retention capacity:
125 μM x 2 = 250 μM
② Amplitude of swelling:
152 mm – 100%
73 mm – x → 48.03%
③ Rate of swelling:
85 mm – 10 min
9.5 – x → 1.1176 min
14 mm – 0.1 OD
63 mm – x → 0.45 OD
Rate of swelling = 0.4026 OD/min
73
63
9.5
152 = 100% swelling.
14 mm = = 0.1 OD
85 mm – 10 min

LIVER

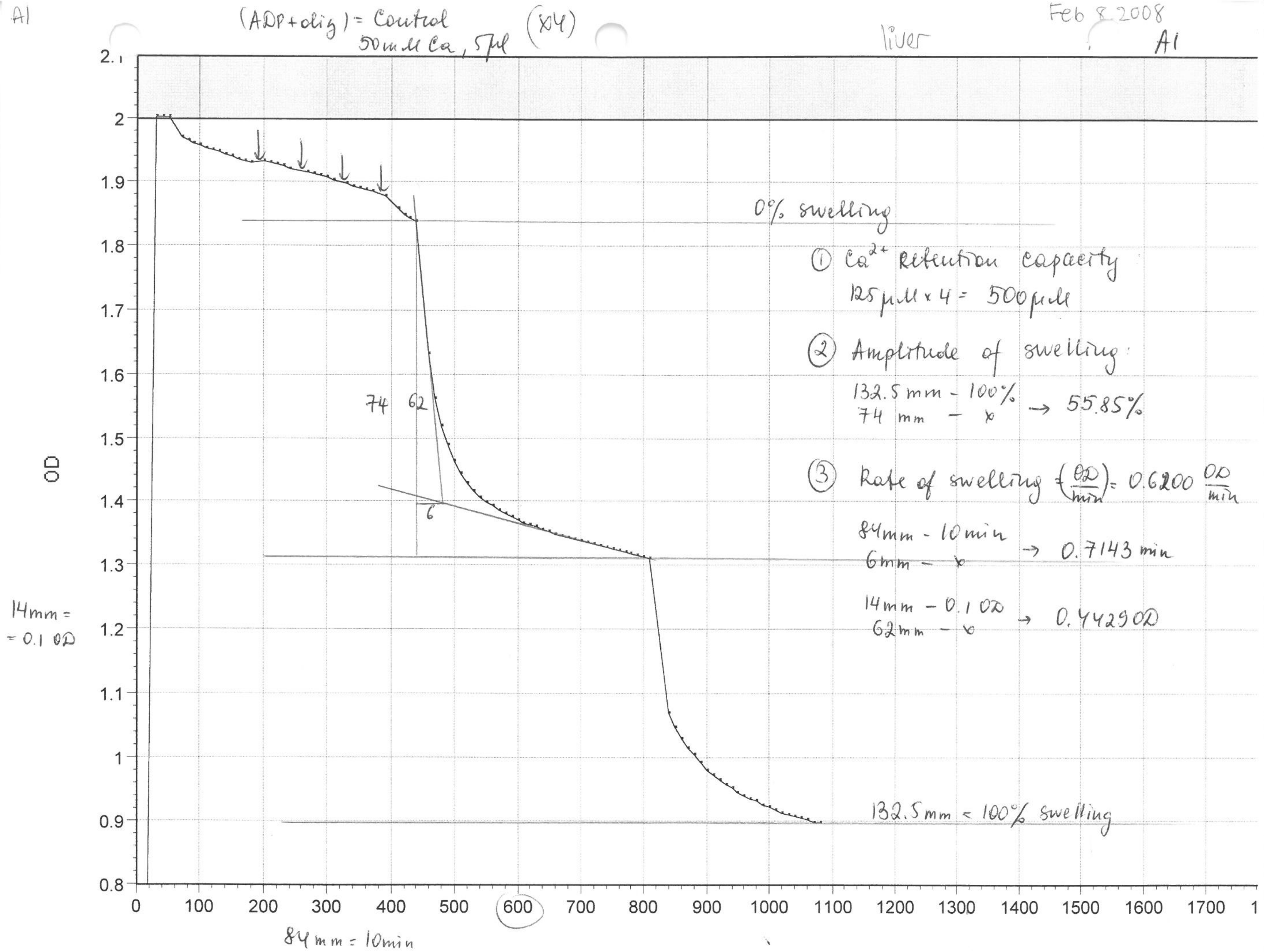

A1
(ADP+olig) = Control
50 mM Ca, 5 µl
(x4)
Feb 8 2008
liver
A1
OD
14mm = = 0.1 OD
84 mm = 10min
74
62
6
0% swelling
① Ca2+ Retention capacity
125 µM x 4 = 500 µM
② Amplitude of swelling:
132.5 mm - 100%
74 mm - x → 55.85%
③ Rate of swelling (OD/min) = 0.6200 OD/min
84mm - 10 min
6mm - x → 0.7143 min
14mm - 0.1 OD
62mm - x → 0.4429 OD
132.5mm = 100% swelling

LIVER

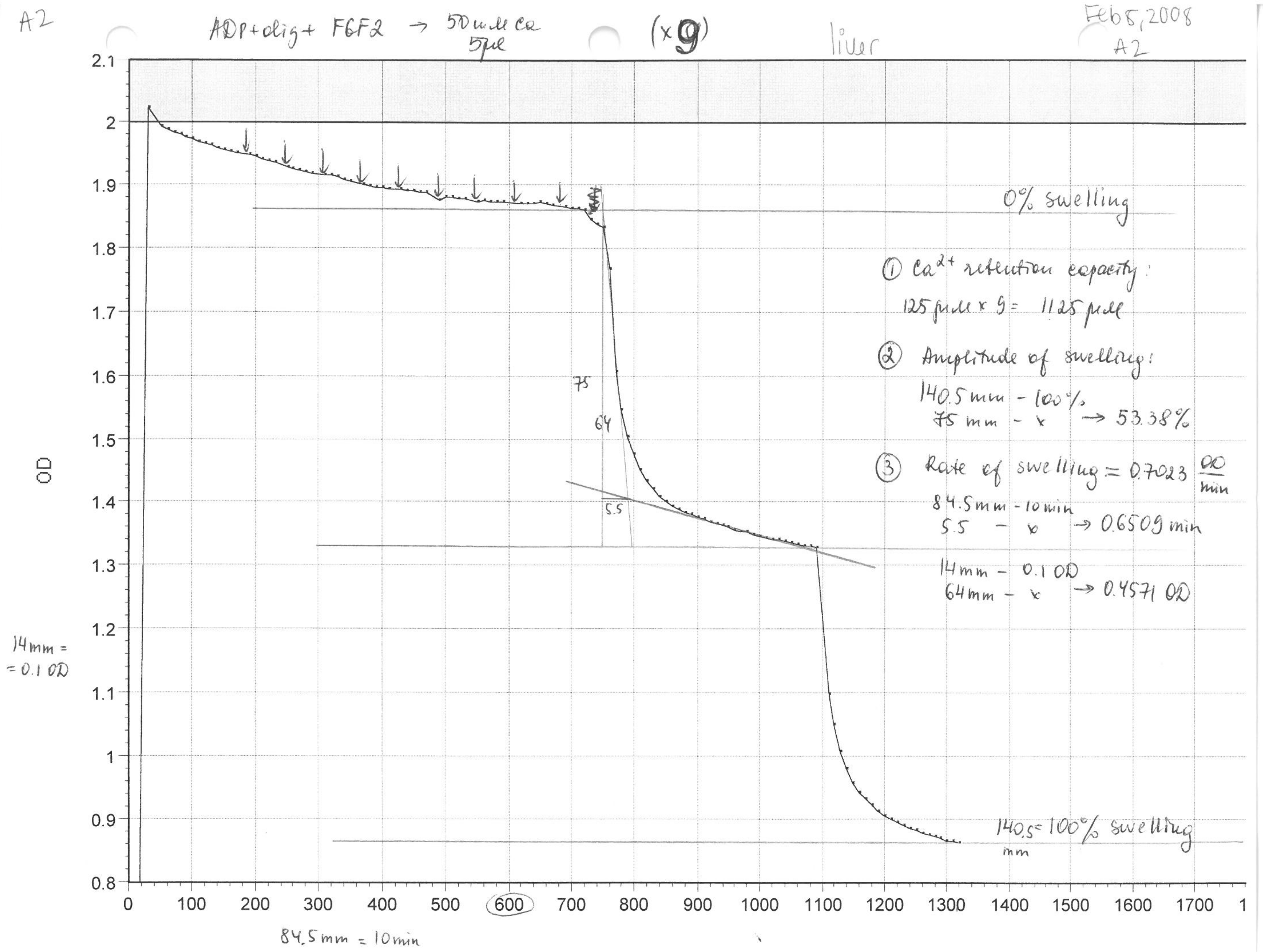

A2
ADP+olig+ FGF2 → 50 uM Ca 5 μl
(x9)
liver
Feb 5, 2008 A2
OD
Time axis: 0, 100, 200, 300, 400, 500, 600, 700, 800, 900, 1000, 1100, 1200, 1300, 1400, 1500, 1600, 1700, 1
OD axis: 0.8, 0.9, 1, 1.1, 1.2, 1.3, 1.4, 1.5, 1.6, 1.7, 1.8, 1.9, 2, 2.1
0% swelling
① Ca2+ retention capacity:
125 pmol x 9 = 1125 pmol
② Amplitude of swelling:
140.5 mm - 100%
75 mm - x → 53.38%
③ Rate of swelling = 0.7023 OD/min
84.5 mm - 10 min
5.5 - x → 0.6509 min
14 mm - 0.1 OD
64 mm - x → 0.4571 OD
75
64
5.5
14 mm = = 0.1 OD
140.5 mm = 100% swelling
84.5 mm = 10 min

LIVER

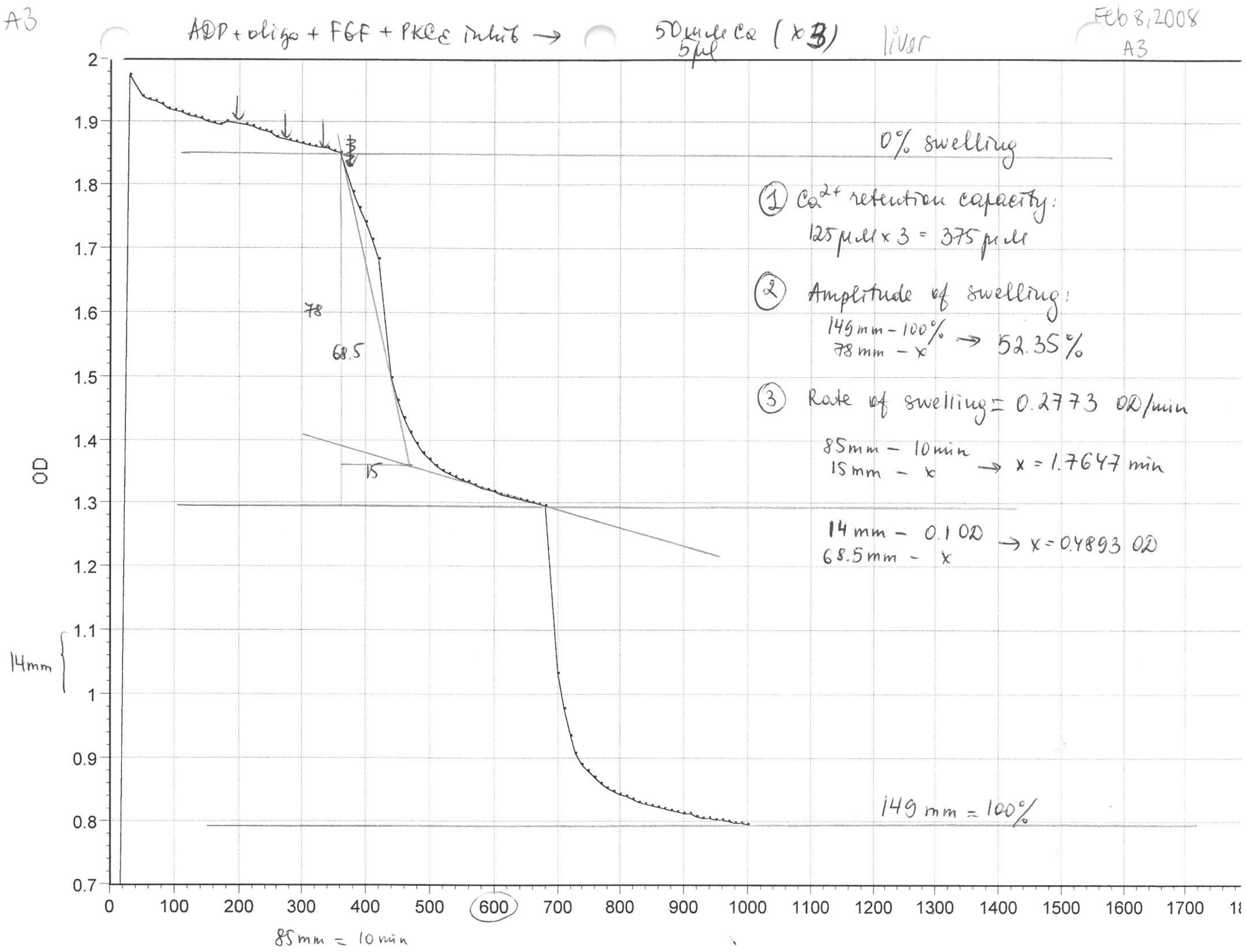

A3
ADP + oligo + FGF + PKCε inhib → 50 μmole Ca (x3) 5μl
liver
Feb 8, 2008
A3
OD
0% swelling
① Ca2+ retention capacity:
125 μM x 3 = 375 μM
② Amplitude of swelling:
149 mm - 100%
78 mm - x → 52.35%
③ Rate of swelling = 0.2773 OD/min
85 mm - 10 min
15 mm - x → x = 1.7647 min
14 mm - 0.1 OD
68.5 mm - x → x = 0.4893 OD
78
68.5
15
14mm
149 mm = 100%
85 mm = 10 min

LIVER

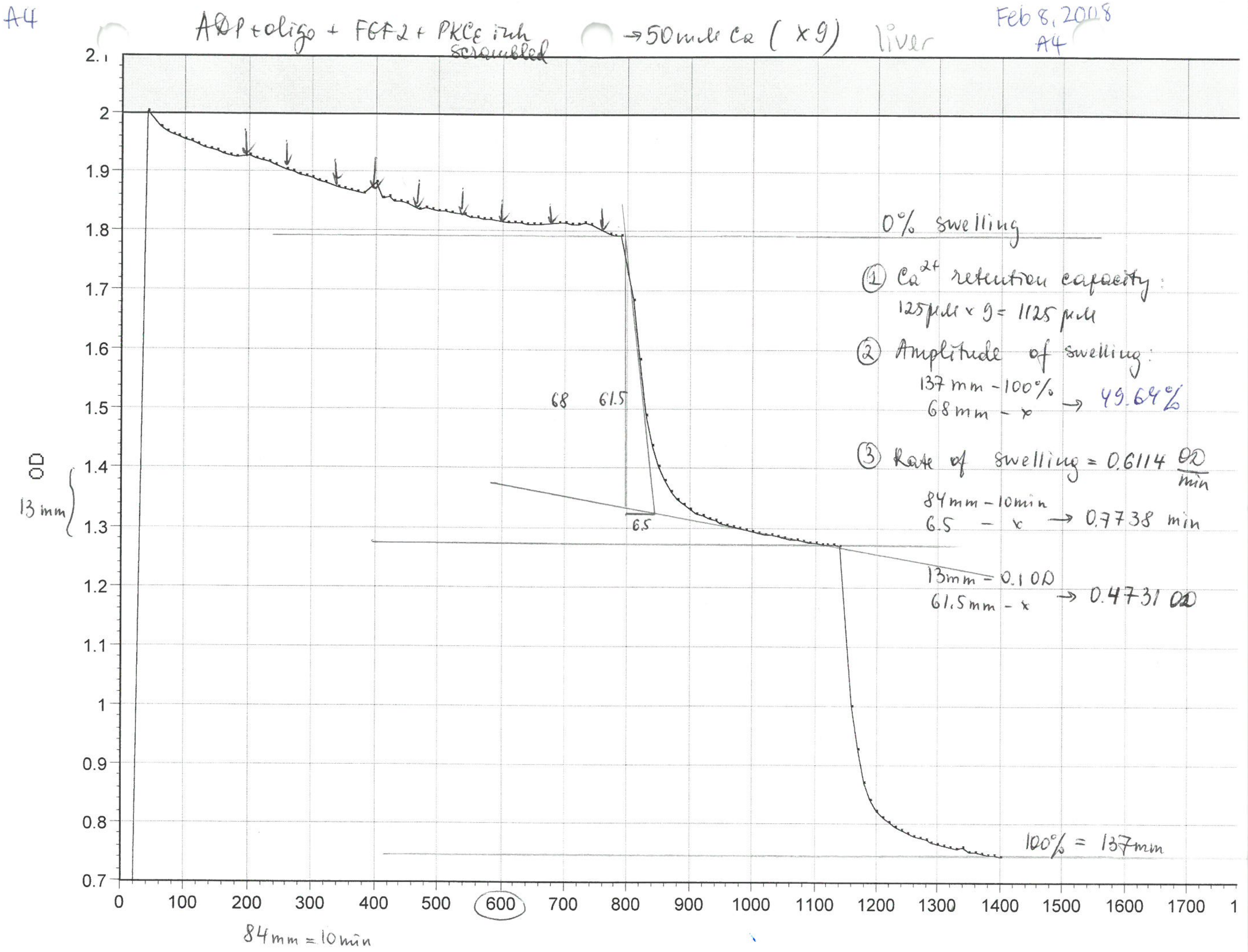
A4
ADP+oligo + FGF2 + PKCε inh scrambled
→50 μM Ca (x 9)
liver
Feb 8, 2008
A4
OD
13 mm
0% swelling
① Ca2+ retention capacity:
125 μM x 9 = 1125 μM
② Amplitude of swelling:
137 mm - 100%
68 mm - x
→ 49.64%
③ Rate of swelling = 0.6114 OD/min
84 mm - 10 min
6.5 - x
→ 0.7738 min
13 mm = 0.1 OD
61.5 mm - x
→ 0.4731 OD
68
61.5
6.5
100% = 137 mm
84 mm = 10 min
2.1
2
1.9
1.8
1.7
1.6
1.5
1.4
1.3
1.2
1.1
1
0.9
0.8
0.7
0
100
200
300
400
500
600
700
800
900
1000
1100
1200
1300
1400
1500
1600
1700

LIVER

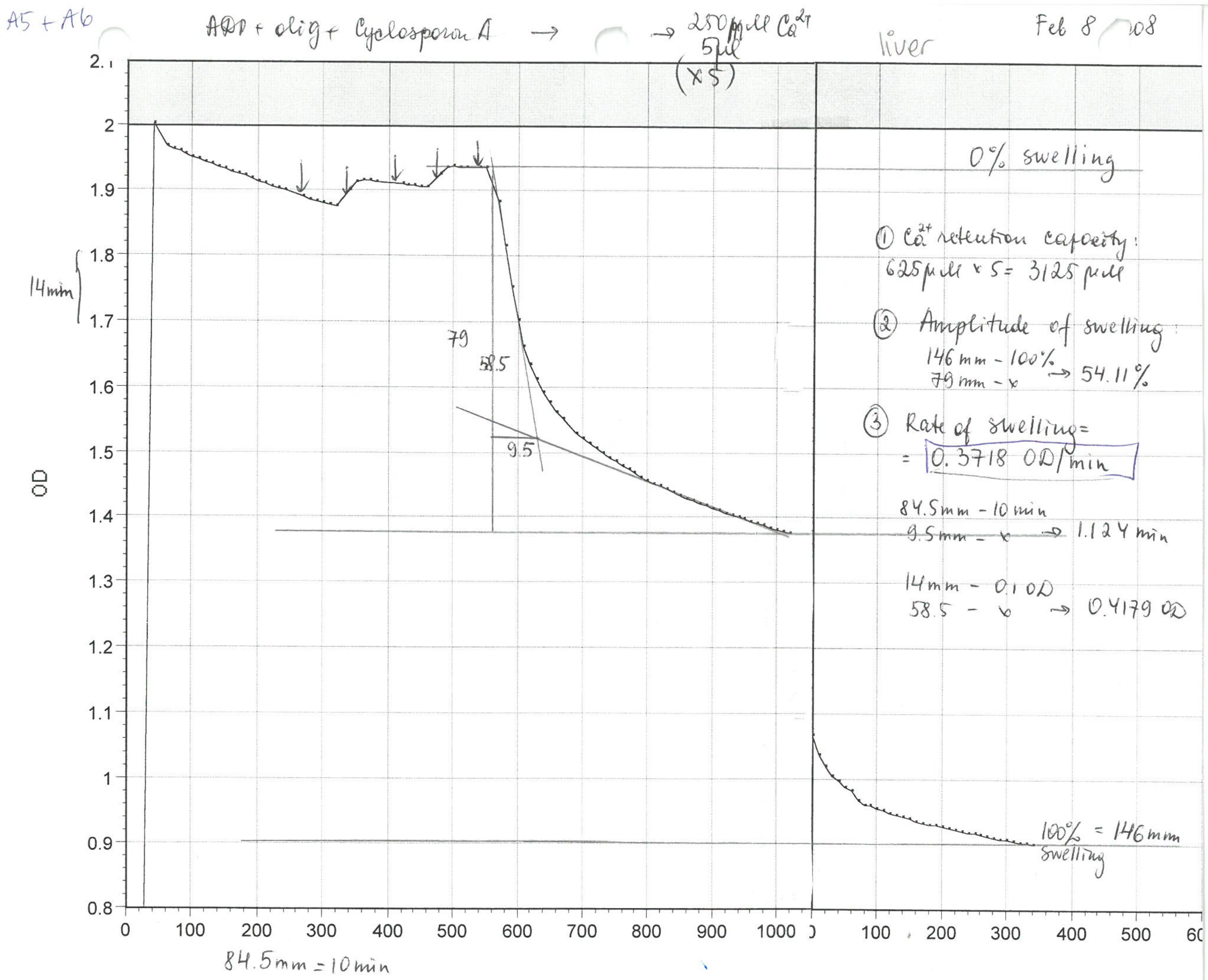

A5 + A6
ADP + olig + Cyclosporin A → → 250 μM Ca2+ 5 μl (x5)
liver
Feb 8 08
OD
14 min
79
58.5
9.5
0% swelling
① Ca2+ retention capacity: 625 μM x 5 = 3125 μM
② Amplitude of swelling: 146 mm - 100%, 79 mm - x → 54.11%
③ Rate of swelling = = 0.3718 OD/min
84.5 mm - 10 min, 9.5 mm - x → 1.124 min
14 mm - 0.1 OD, 58.5 - x → 0.4179 OD
100% = 146 mm swelling
84.5 mm = 10 min
2.1
2
1.9
1.8
1.7
1.6
1.5
1.4
1.3
1.2
1.1
1
0.9
0.8
0
100
200
300
400
500
600
700
800
900
1000

LIVER

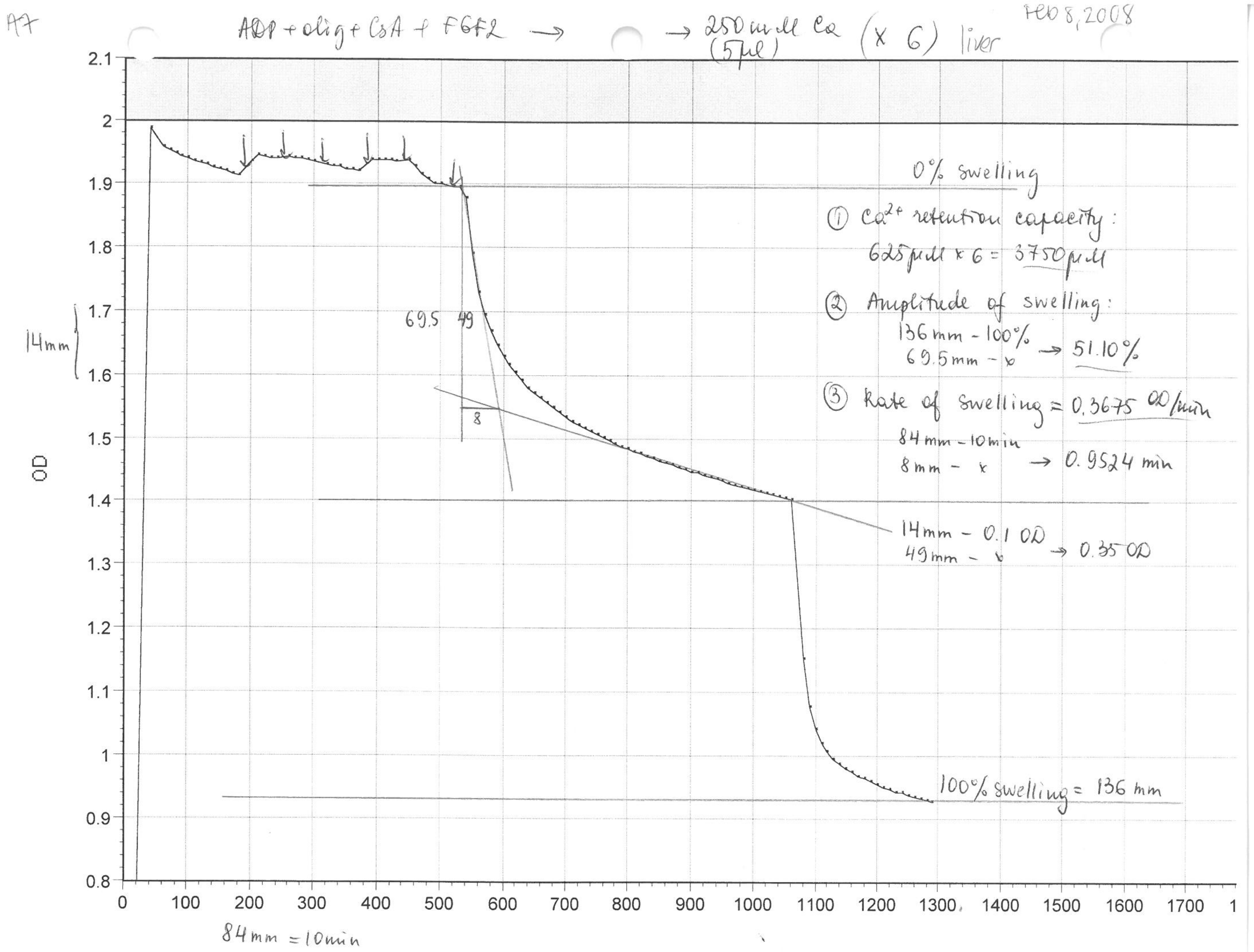
A7
ADP + olig + CsA + FGF2 → → 250 mM Ca (5 µl) (x 6) liver
Feb 8, 2008
OD
0% swelling
① Ca2+ retention capacity: 625 µM x 6 = 3750 µM
② Amplitude of swelling: 136 mm - 100% 69.5 mm - x → 51.10%
③ Rate of swelling = 0.3675 OD/min
84 mm - 10 min 8 mm - x → 0.9524 min
14 mm - 0.1 OD 49 mm - x → 0.35 OD
100% swelling = 136 mm
69.5 49
8
14mm
84mm = 10min

LIVER

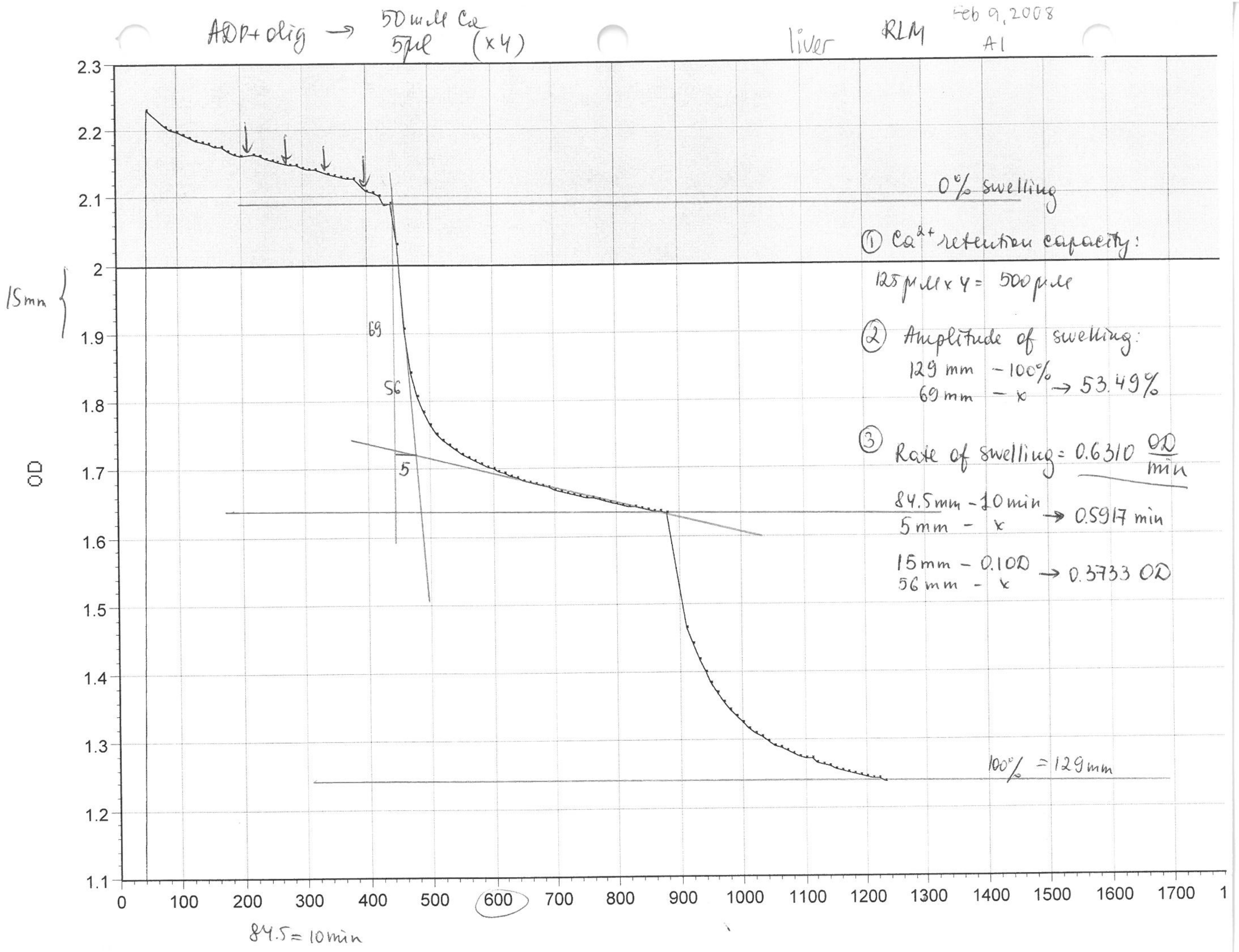
ADP+olig → 50 mM Ca 5μl (x4)
liver
RLM
Feb 9, 2008
A1
2.3
2.2
2.1
2
1.9
1.8
1.7
1.6
1.5
1.4
1.3
1.2
1.1
OD
15mm
0% swelling
① Ca^{2+} retention capacity:
125 μM x 4 = 500 μM
② Amplitude of swelling:
129 mm - 100%
69 mm - x → 53.49%
③ Rate of swelling = 0.6310 OD/min
84.5mm - 10 min
5mm - x → 0.5917 min
15mm - 0.1OD
56mm - x → 0.3733 OD
69
56
5
100% = 129mm
0
100
200
300
400
500
600
700
800
900
1000
1100
1200
1300
1400
1500
1600
1700
84.5 = 10min

LIVER

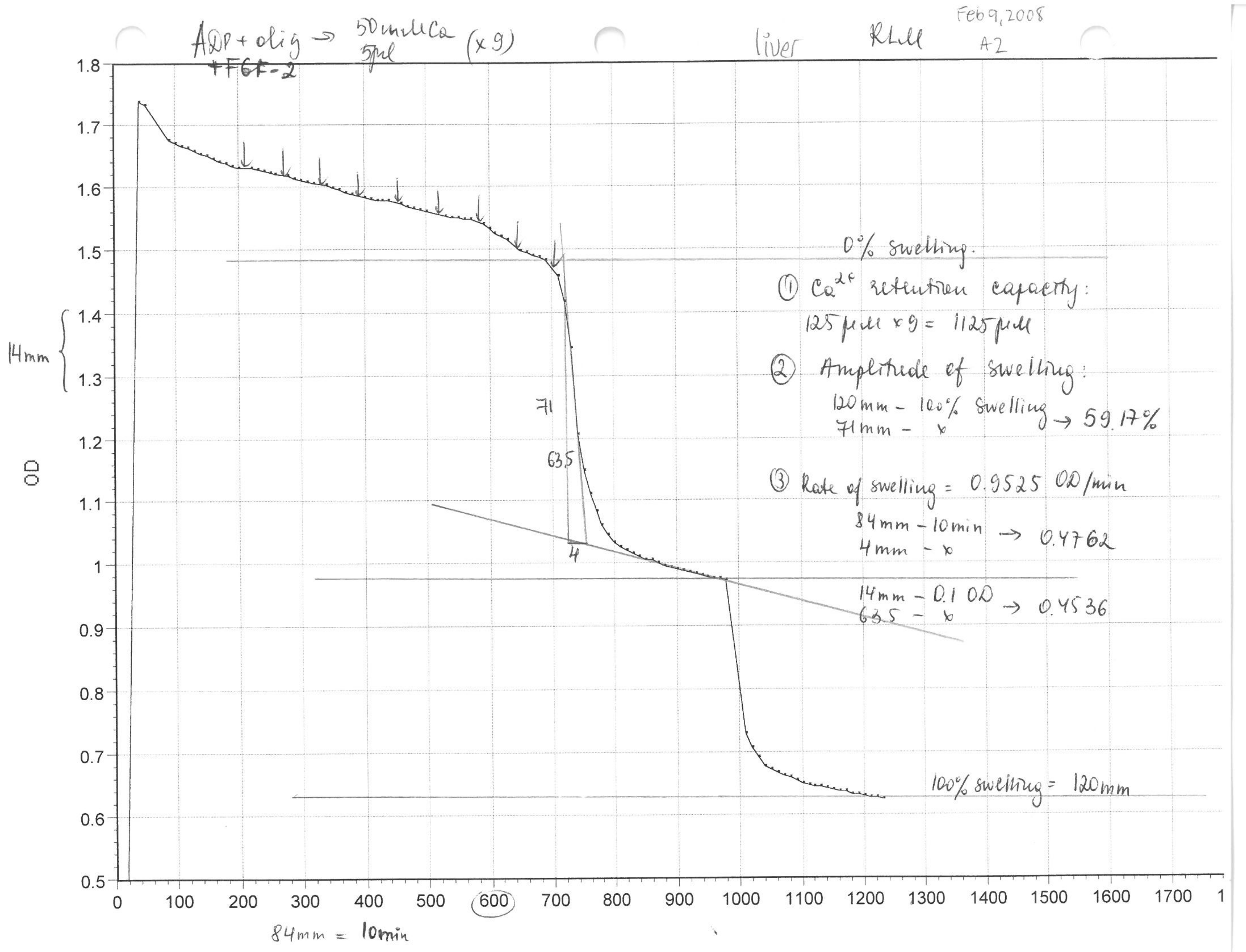
ADP + olig → 50 nmol Ca 5 µl (x 9)
+FGF-2
liver
RLM
Feb 9, 2008
A2
0% swelling.
① Ca^{2+} retention capacity:
125 µM x 9 = 1125 µM
② Amplitude of swelling:
120 mm – 100% swelling
71 mm – x → 59.17%
③ Rate of swelling = 0.9525 OD/min
84 mm – 10 min
4 mm – x → 0.4762
14 mm – 0.1 OD
63.5 – x → 0.4536
100% swelling = 120 mm
14mm
OD
71
63.5
4
84mm = 10min

LIVER

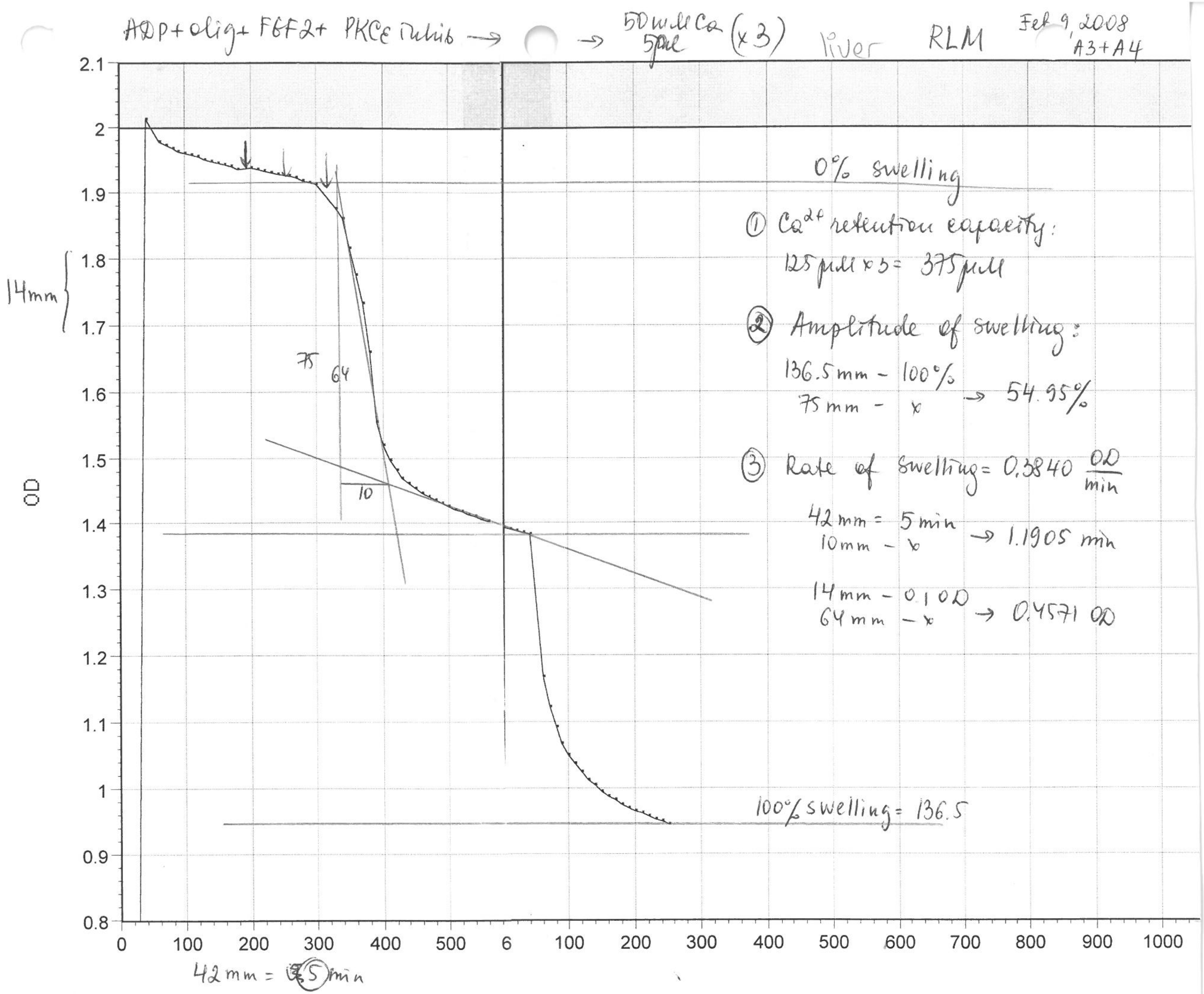

ADP+olig+FGF2+ PKCε inhib → → 50 µM Ca 5 µl (x3)
liver
RLM
Feb 9, 2008
A3+A4
OD
14mm
75
64
10
0% swelling
① Ca2+ retention capacity:
125 µM x 3 = 375 µM
② Amplitude of swelling:
136.5 mm - 100%
75 mm - x → 54.95%
③ Rate of swelling = 0.3840 OD/min
42 mm = 5 min
10 mm - x → 1.1905 min
14 mm - 0.1 OD
64 mm - x → 0.4571 OD
100% swelling = 136.5
42 mm = 5 min

LIVER

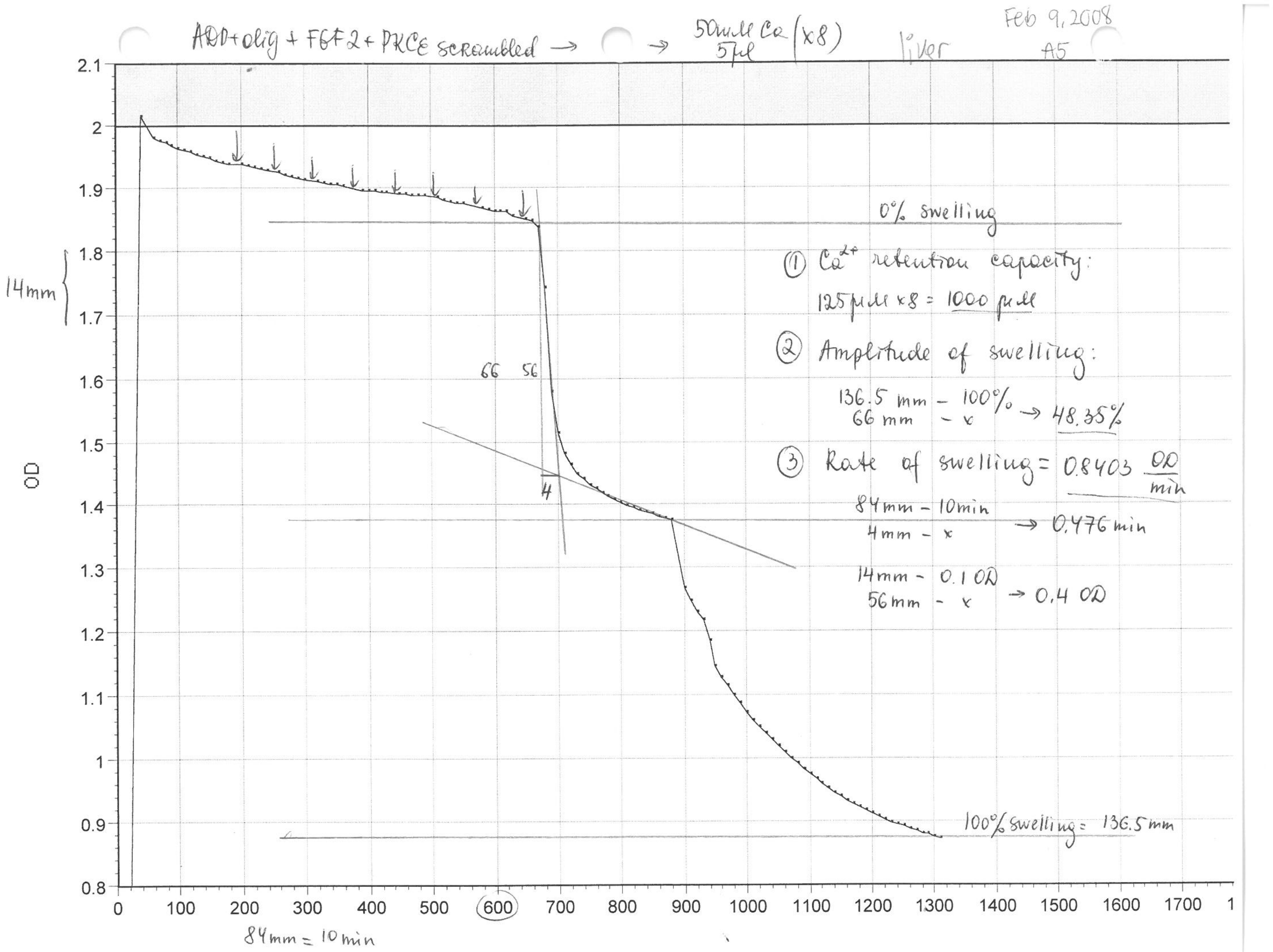

ADP+olig + FGF2 + PKCε scrambled → → 50mM Ca (x8) 5μl
liver
Feb 9, 2008
A5
0% swelling
① Ca2+ retention capacity:
125 μM x 8 = 1000 μM
② Amplitude of swelling:
136.5 mm – 100%
66 mm – x → 48.35%
③ Rate of swelling = 0.8403 OD/min
84 mm – 10 min
4 mm – x → 0.476 min
14 mm – 0.1 OD
56 mm – x → 0.4 OD
100% swelling = 136.5 mm
14mm
66 56
4
OD
84mm = 10 min

LIVER

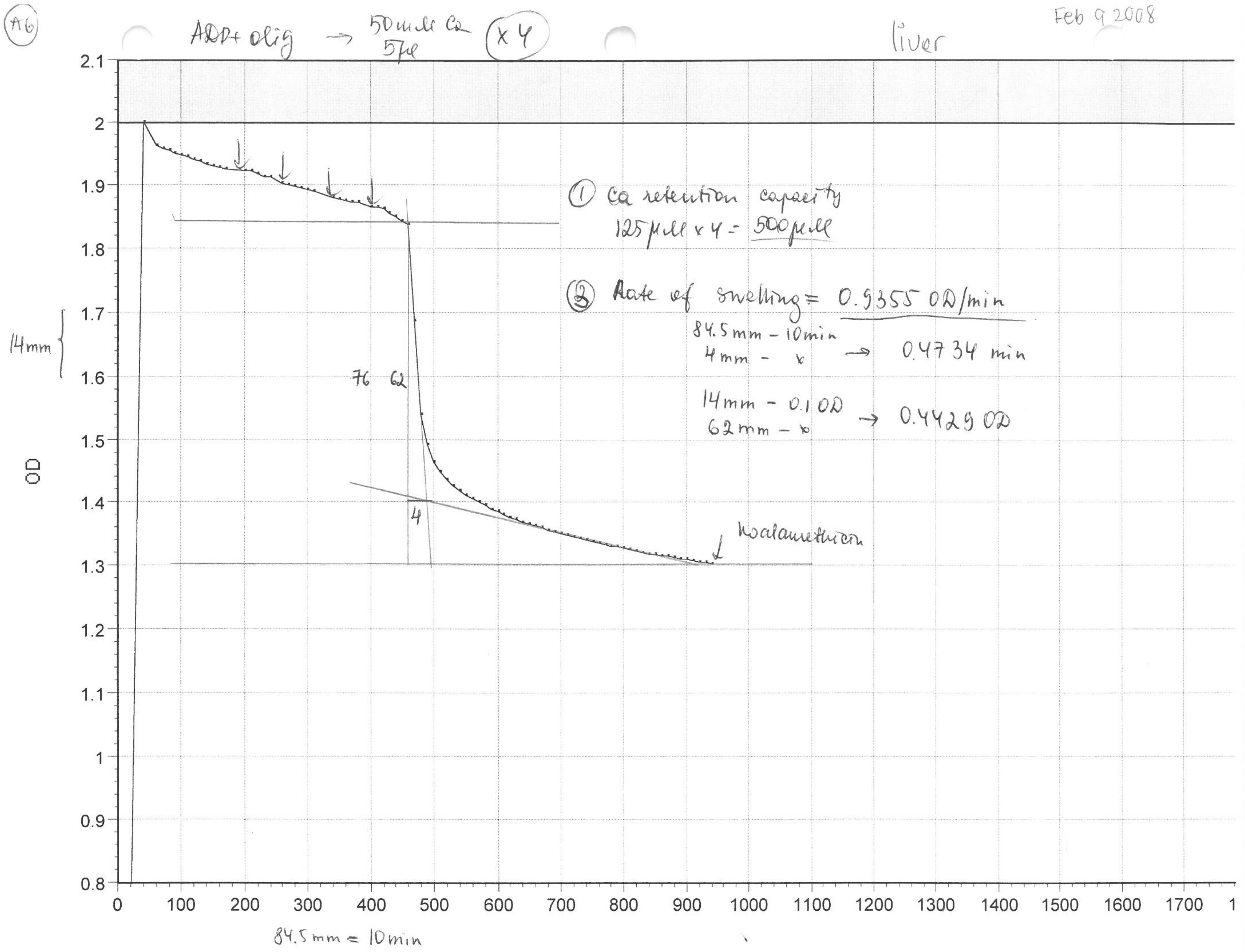
A6
ADD+ olig → 50 mM Ca 5 μl (x 4)
liver
Feb 9 2008
① Ca retention capacity
125 μM x 4 = 500 μM
② Rate of swelling = 0.9355 OD/min
84.5 mm - 10 min
4 mm - x → 0.4734 min
14 mm - 0.1 OD
62 mm - x → 0.4429 OD
76
62
4
14mm
OD
84.5 mm = 10 min
2.1
2
1.9
1.8
1.7
1.6
1.5
1.4
1.3
1.2
1.1
1
0.9
0.8
0
100
200
300
400
500
600
700
800
900
1000
1100
1200
1300
1400
1500
1600
1700

LLIVER

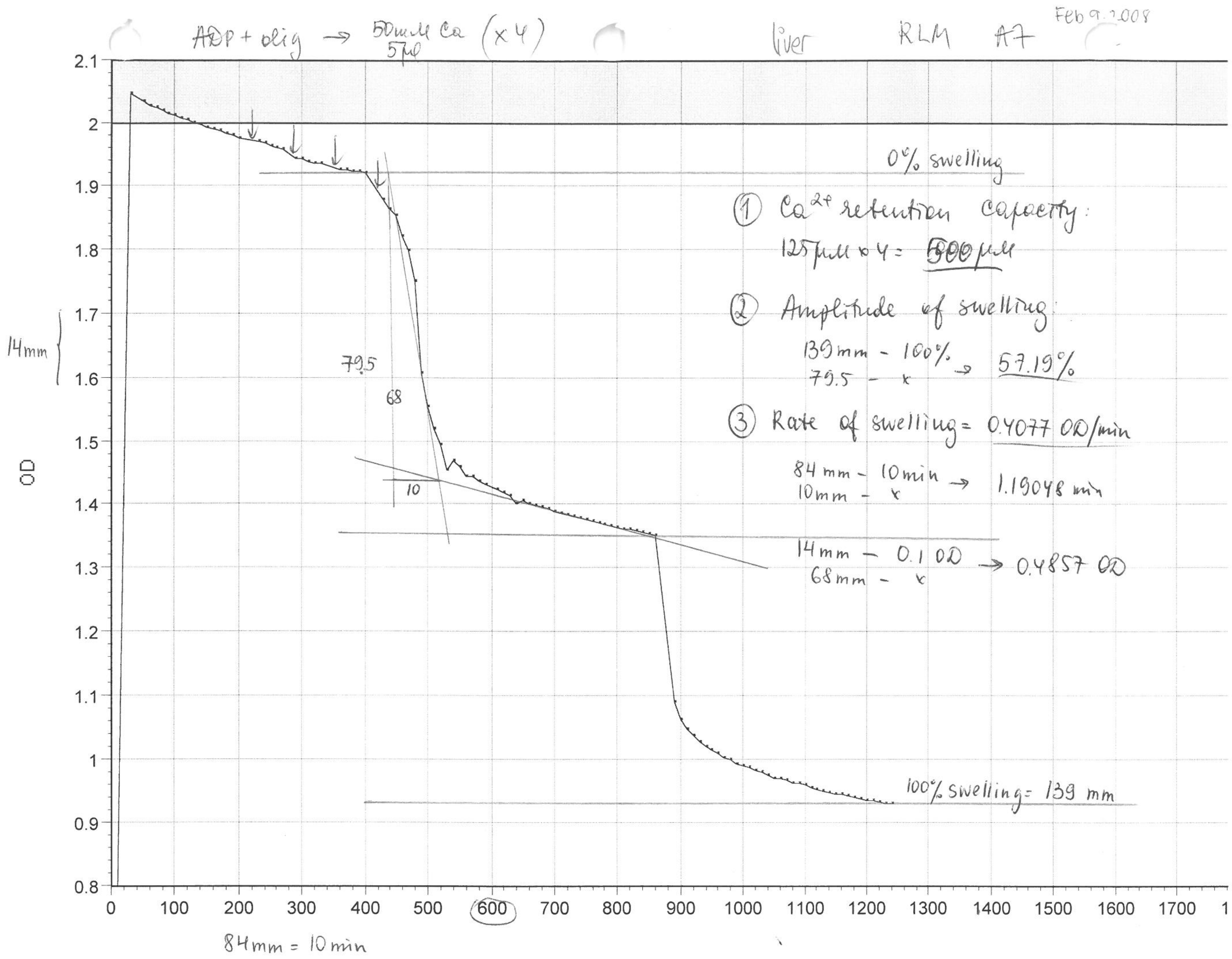
Feb 9 2008
ADP + olig → 50mM Ca (x4)
5μl
liver
RLM
A7
OD
14mm
0% swelling
① Ca2+ retention capacity:
125μM x 4 = 500μM
② Amplitude of swelling:
139mm - 100%
79.5 - x
→ 57.19%
③ Rate of swelling = 0.4077 OD/min
84 mm - 10min
10mm - x
→ 1.19048 min
14mm - 0.1 OD
68mm - x
→ 0.4857 OD
79.5
68
10
100% swelling = 139 mm
84mm = 10 min
2.1
2
1.9
1.8
1.7
1.6
1.5
1.4
1.3
1.2
1.1
1
0.9
0.8
0
100
200
300
400
500
600
700
800
900
1000
1100
1200
1300
1400
1500
1600
1700

Original blots for Fig.3

Fig.3a

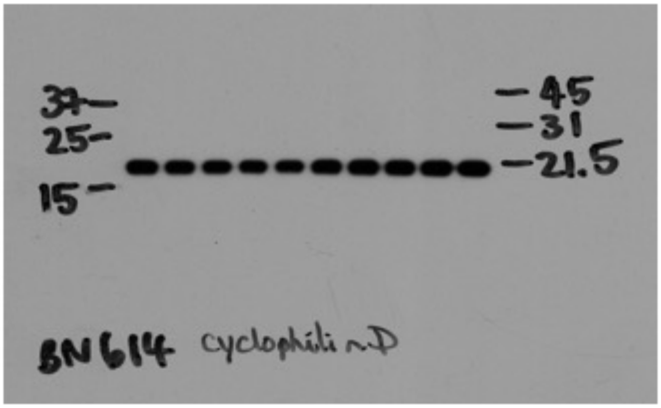

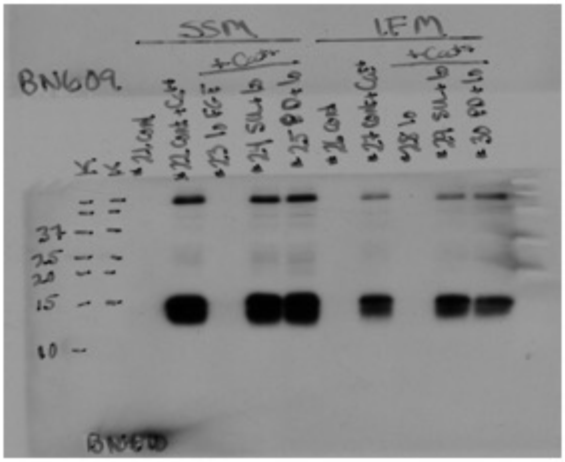

Fig.3b

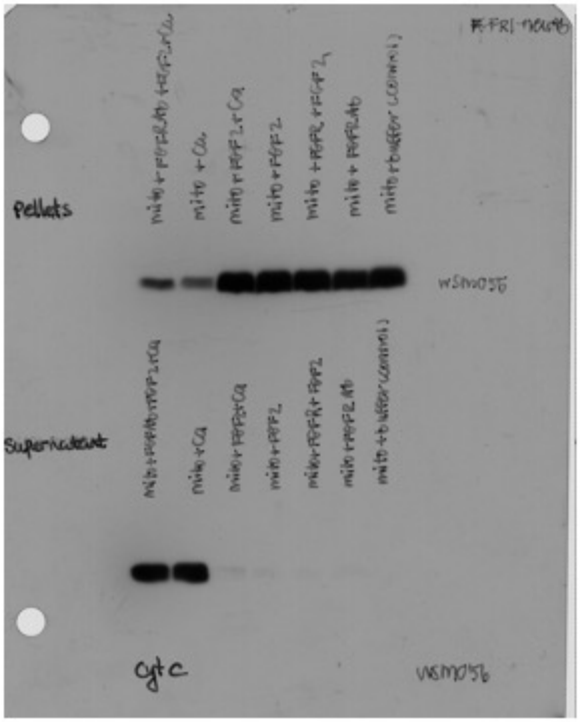

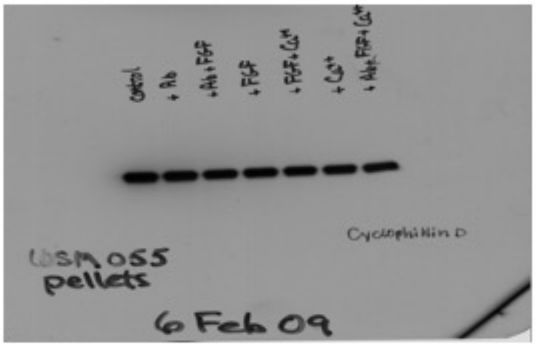

Original blots for Fig.2a

x anti-FGFR1, QED A/B.

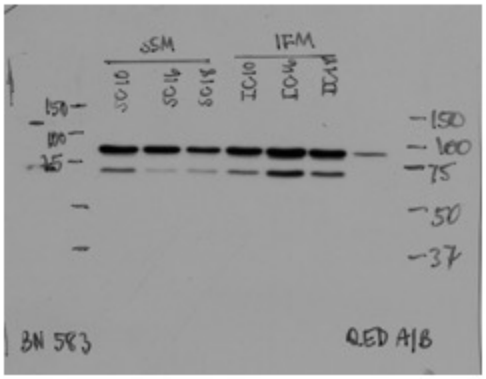

x anti-pY766-FGFR1

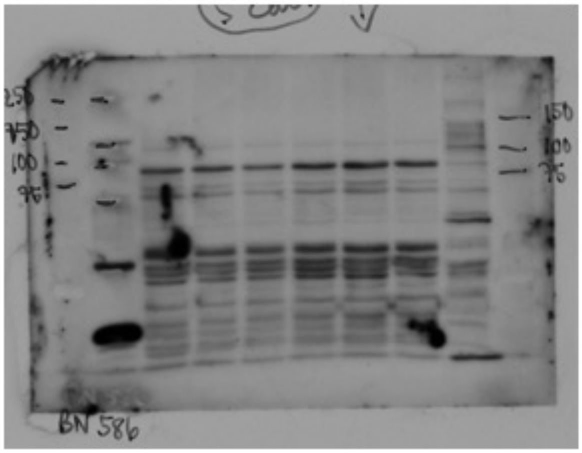

x anti-pY653/654-FGFR1.

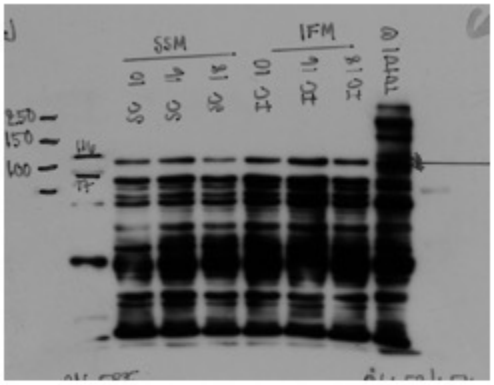

Ponceau S stain

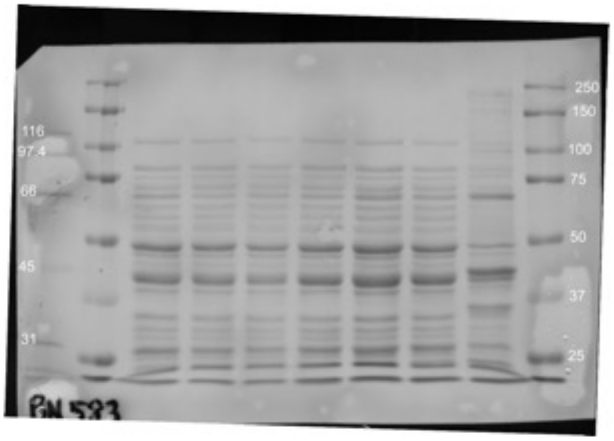

Original images for Fig.2b

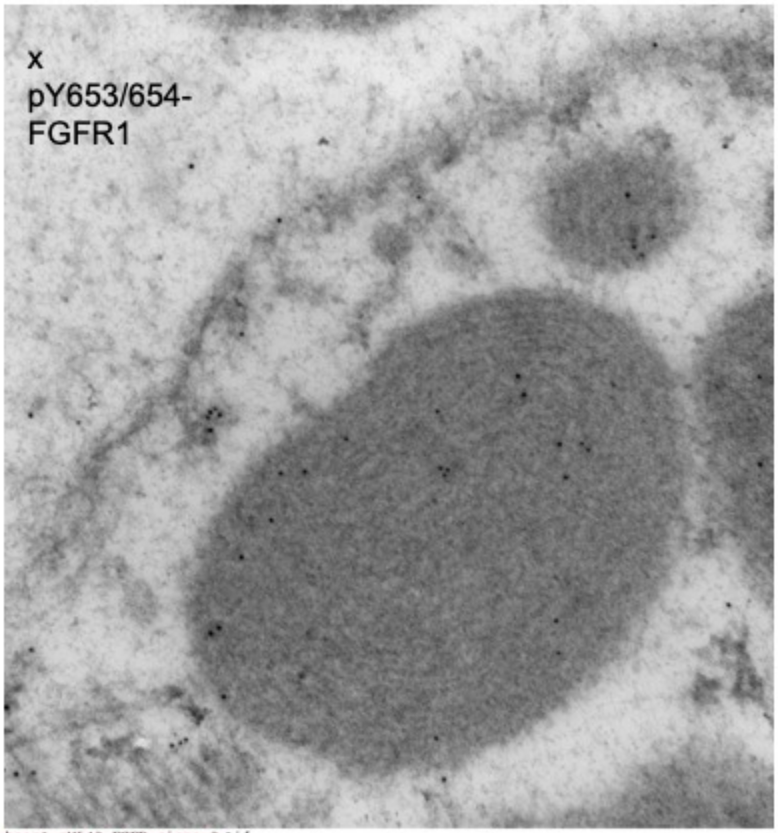

Mislabeled- ~~heart-pY543-FGFR-sigma-1~~.tif
HEART SECTION
Print Mag: 116000x @ 7.0 in
11:57 10/31/08
ito

100 nm
HV=80.0kV
Direct Mag: 100000x
Facility of Medicine EM Unit

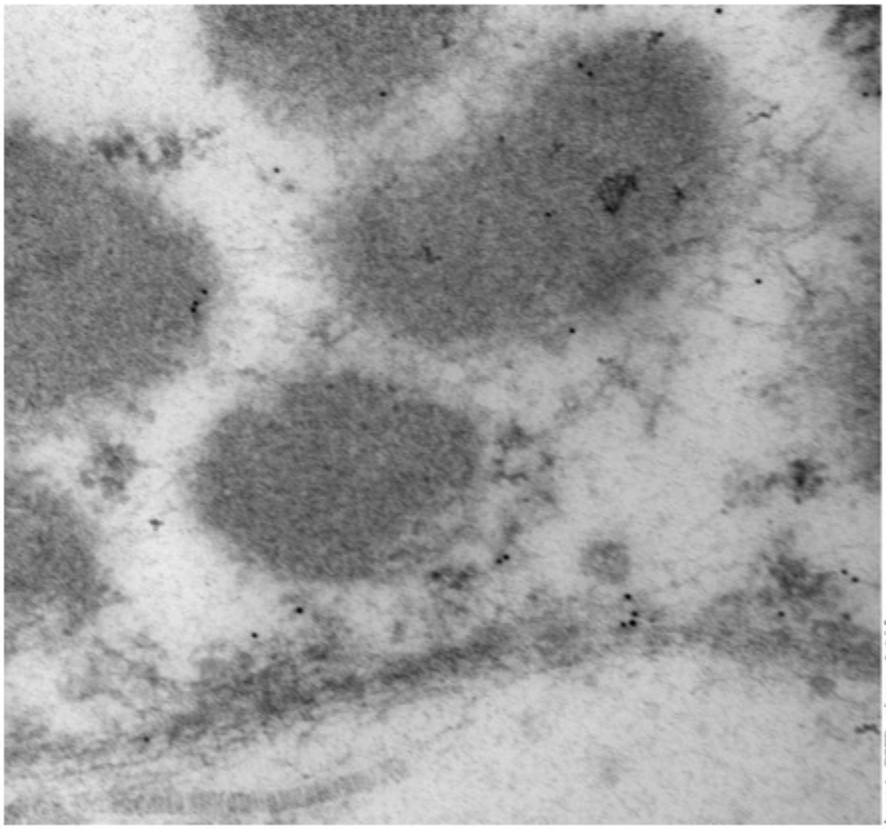

heart-FGFR-sigma-2.tif
HEART SECTION
Print Mag: 140000x @ 7.0 in
11:26 10/31/08
ito

100 nm
HV=80.0kV
Direct Mag: 120000x
Facility of Medicine EM Unit

Original blots for Fig.4

Fig.4a

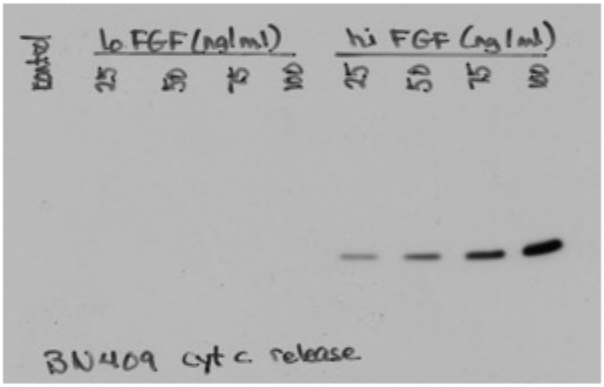

Fig.4b

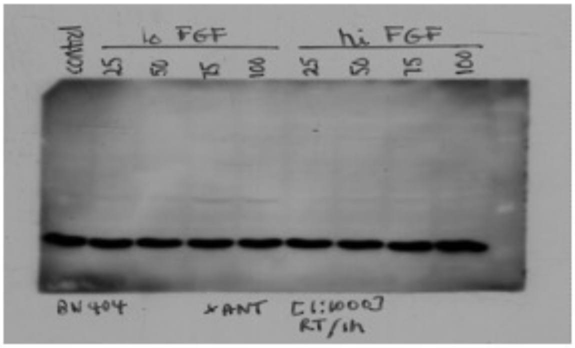

Fig.4c

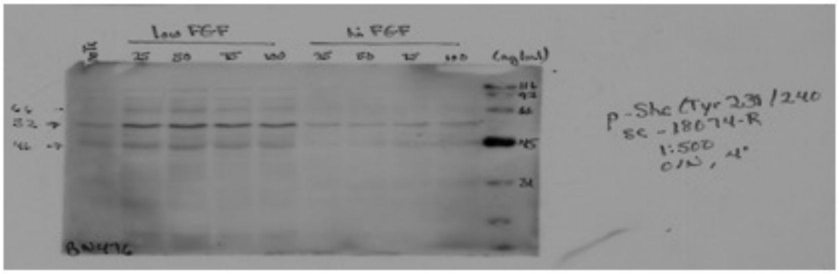

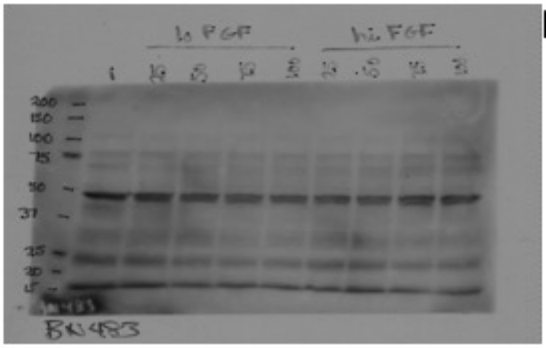

Fig.4d

Fig.4e

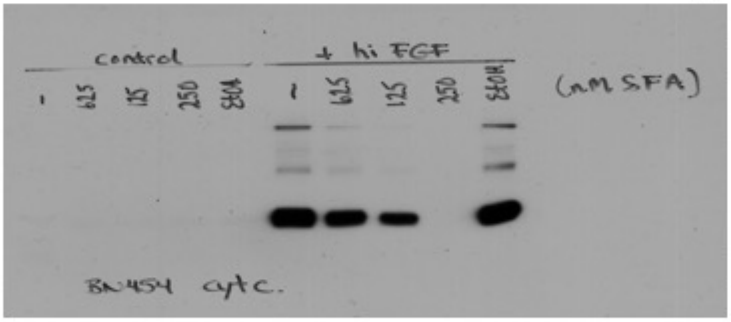

Original blots for Fig.5

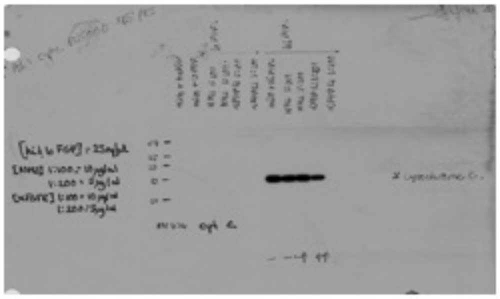

Fig.5a

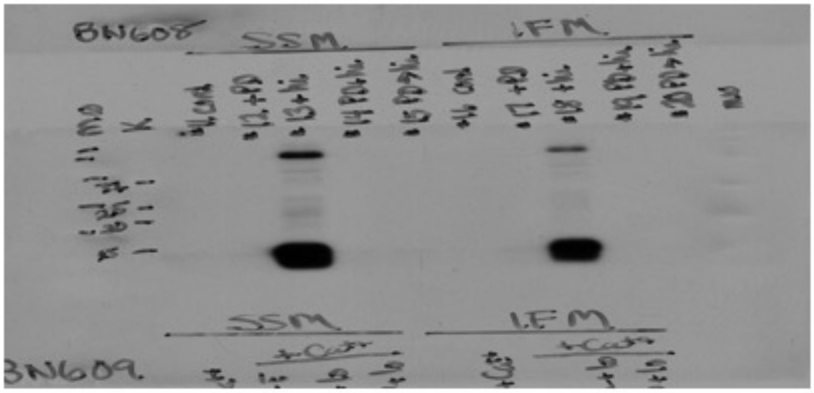

Fig.5b

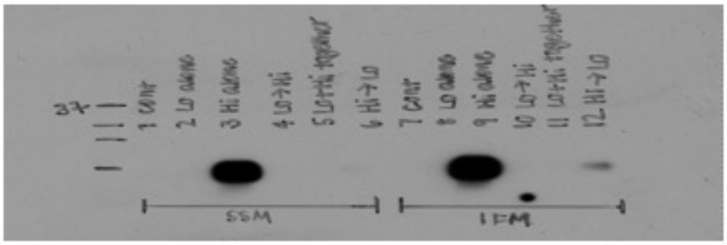

Fig.5c

Fig.5e

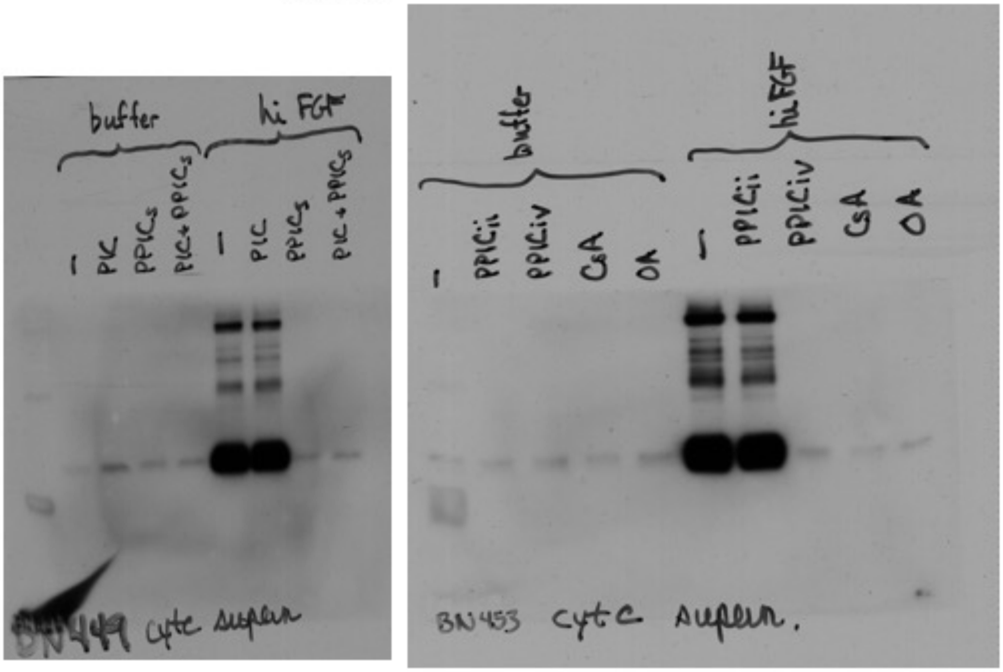

Repeat experiment: SSM and IFM preparations probed with anti-pY653/654-FGFR1

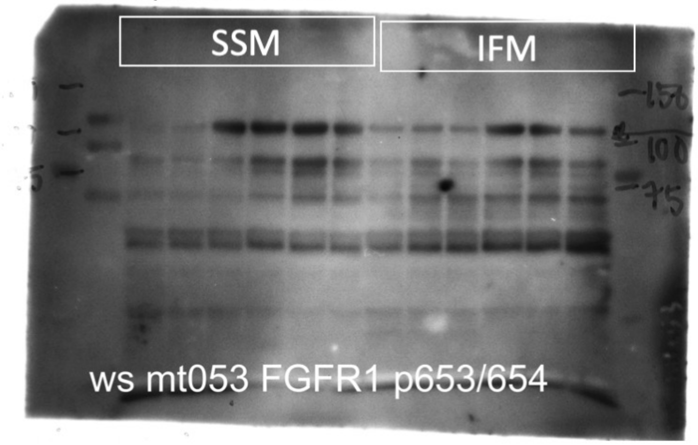

Repeat experiment: SSM and IFM preparations probed with anti-pY653/654-FGFR1

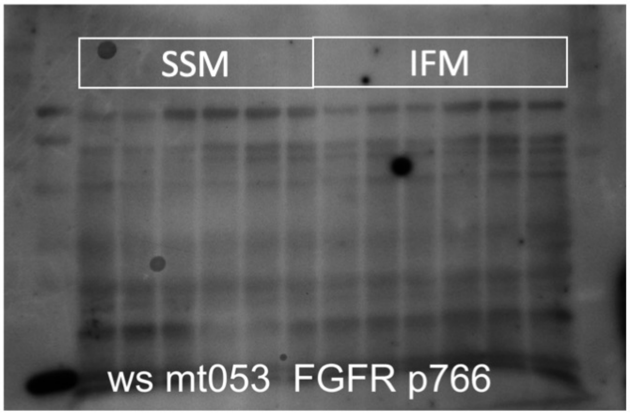

Repeat experiments: Western blots showing supernatant cytochrome c after exposure to Hi-FGF2, and Lo-FGF2

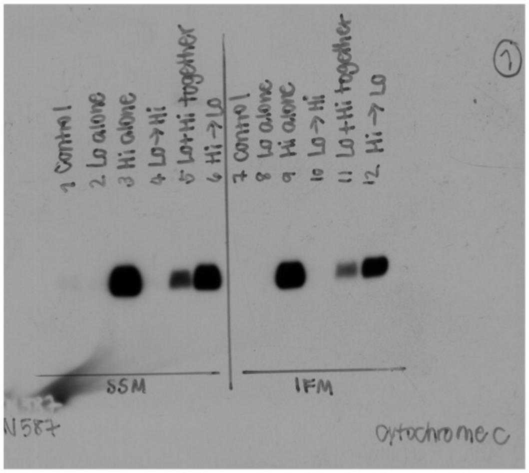

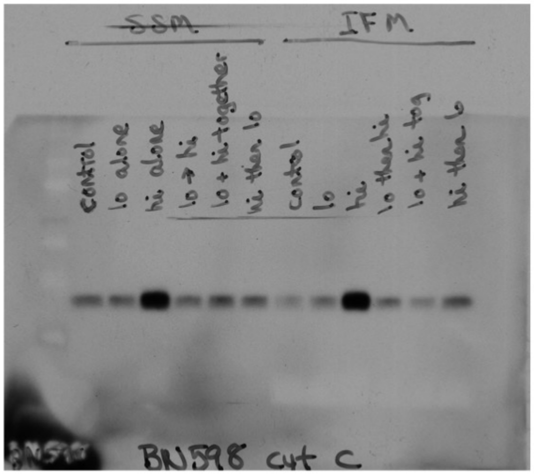

Repeat experiments: Western blots showing supernatant cytochrome c after exposure to Hi-FGF2 in the presence of non-immune antibodies (upper gel) or neutralizing antibodies (lower gel)

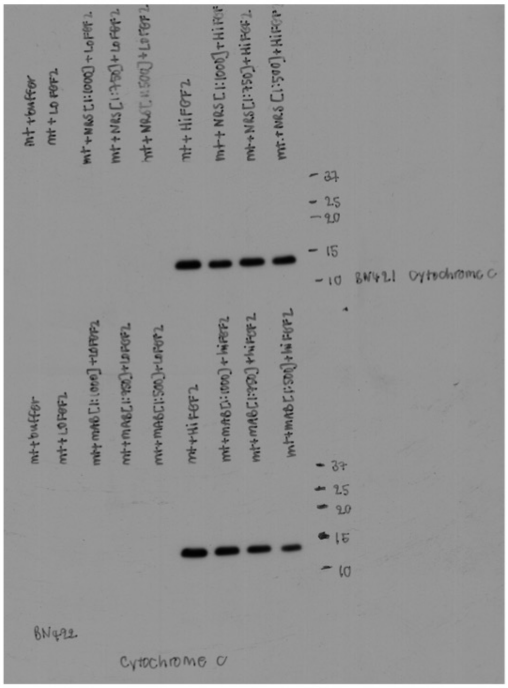

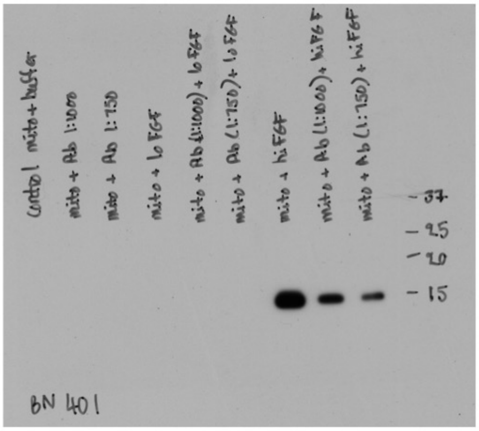

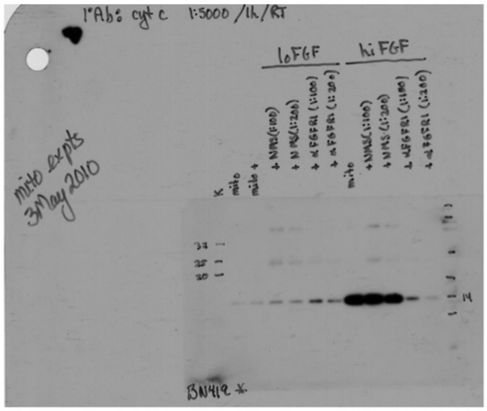

Repeat experiment: SSM and IFM treated with Lo-FGF2 in the presence and absence of FGFR1 inhibitors (SU-, PD-) and subjected to calcium overload: Cyt-C at supernatant

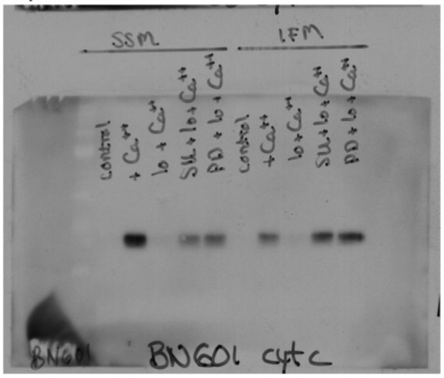

Repeat experiment: SSM treated with Lo-FGF2 in the presence and absence of FGFR1 inhibitor SU-, and subjected to calcium overload: Cyt-C at supernatant and at particulate fraction

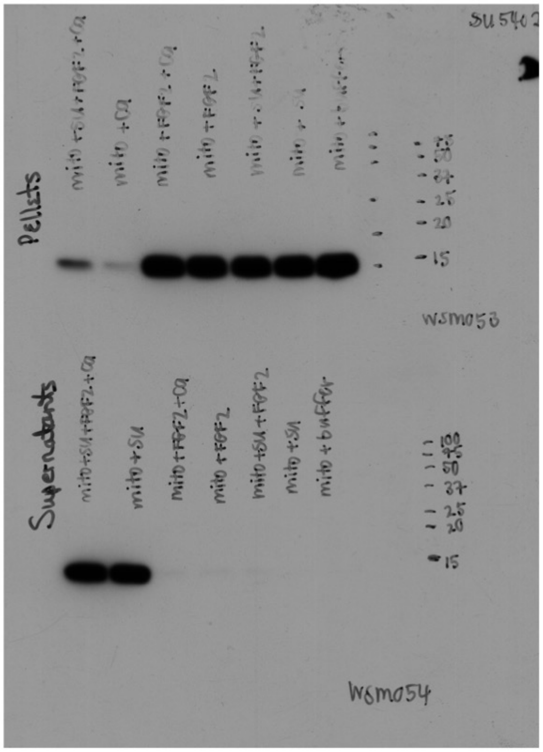

Repeat experiment: SSM treated with Lo-FGF2 in the presence and absence of FGFR1 inhibitor SU-, and subjected to calcium overload: Cyt-C at supernatant and at particulate fraction

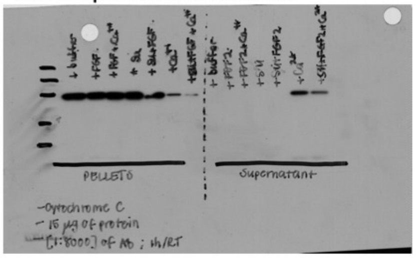

Repeat experiments: Western blots of SSM and IFM treated with Hi-FGF2 in the presence and absence of FGFR1 inhibitor PD-, SU-: Cyt-C at supernatant

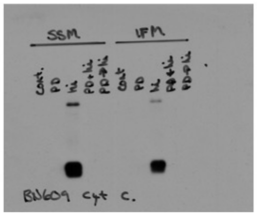

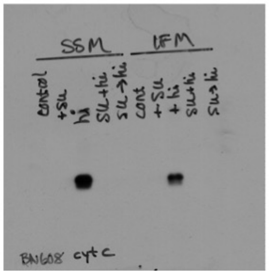

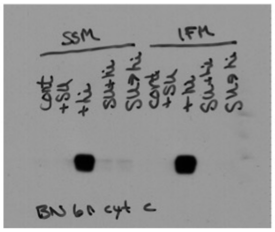

Supplement: Supplementary file 1 [file cells-10-02735-s001.zip › cells-1366596-supplementary.pdf]
